# Guidelines on vascular access for hemodialysis from the Brazilian Society of Angiology and Vascular Surgery

**DOI:** 10.1590/1677-5449.202300522

**Published:** 2023-10-20

**Authors:** Leonardo de Oliveira Harduin, Thiago Almeida Barroso, Julia Bandeira Guerra, Marcio Gomes Filippo, Leonardo Cortizo de Almeida, Guilherme de Castro-Santos, Fabio Augusto Cypreste Oliveira, Douglas Eduardo Tavares Cavalcanti, Ricardo Jayme Procopio, Eduardo Cavalcanti Lima, Matheus Eduardo Soares Pinhati, José Maciel Caldas dos Reis, Barbara D’Agnoluzzo Moreira, Adriano Martins Galhardo, Edwaldo Edner Joviliano, Walter Junior Boim de Araujo, Julio Cesar Peclat de Oliveira

**Affiliations:** 1 Universidade Estadual do Estado do Rio de Janeiro - UERJ, Departamento de Cirurgia Vascular, Niterói, RJ, Brasil.; 2 Hospital Sírio Libanês, Brasília, DF, Brasil.; 3 Hospital Niterói D’or, Departamento de Imagem, Niterói, RJ, Brasil.; 4 Universidade Federal do Rio de Janeiro - UFRJ, Departamento de Cirurgia, Rio de Janeiro, RJ, Brasil.; 5 Hospital Ana Nery, Departamento de Cirurgia Vascular e Endovascular, Salvador, BA, Brasil.; 6 Universidade Federal de Minas Gerais - UFMG, Escola de Medicina, Departamento de Cirurgia, Belo Horizonte, MG, Brasil.; 7 Hospital Santa Helena, Departamento de Cirurgia Vascular, Goiânia, GO, Brasil.; 8 Angiorad Recife, Departamento de Cirurgia Vascular, Recife, PE, Brasil.; 9 Instituto de Medicina Integral Prof. Fernando Figueira - IMIP, Recife, PE, Brasil.; 10 Centro Universitário Metropolitano Da Amazônia - Unifama, Técnica Cirúrgica, Belém, PA, Brasil.; 11 Universidade Federal do Paraná - UFPR, Hospital de Clínicas, Serviço de Cirurgia Vascular, Curitiba, PR, Brasil.; 12 Hospital Brasília Águas Claras, Brasília, DF, Brasil.; 13 Universidade de São Paulo - USP, Faculdade de Medicina de Ribeirão Preto - FMRP, Departamento de Anatomia e Cirurgia, Ribeirão Preto, SP, Brasil.; 14 Universidade Federal do Paraná - UFPR, Hospital de Clínicas, Departamento de Angioradiologia e Cirurgia Endovascular, Curitiba, PR, Brasil.; 15 Sociedade Brasileira de Angiologia e Cirurgia Vascular - SBACV, São Paulo, SP, Brasil.

**Keywords:** kidney dialysis, vascular access, guideline

## Abstract

Chronic kidney disease is a worldwide public health problem, and end-stage renal disease requires dialysis. Most patients requiring renal replacement therapy have to undergo hemodialysis. Therefore, vascular access is extremely important for the dialysis population, directly affecting the quality of life and the morbidity and mortality of this patient population. Since making, managing and salvaging of vascular accesses falls within the purview of the vascular surgeon, developing guideline to help specialists better manage vascular accesses for hemodialysis if of great importance. Thus, the objective of this guideline is to present a set of recommendations to guide decisions involved in the referral, evaluation, choice, surveillance and management of complications of vascular accesses for hemodialysis.

## INTRODUCTION

Chronic kidney disease is a global public health problem that is classified into 5 stages. Renal failure, however, is limited to stages 3 through 5, with glomerular filtration rate below 60 mL/min/1.73 m2 for 3 months or longer, regardless of cause.^1^ Stage 5 chronic kidney disease is characterized by a glomerular filtration rate below 15 mL/min/1.73 m2 and includes two phases: the first one is treated conservatively without dialysis; in the second phase, initiation of renal replacement therapy in the form of dialysis or kidney transplant is required to sustain life. Currently, there are approximately 140,000 patients on dialysis in Brazil, and approximately 90% of them undergo renal replacement therapy by hemodialysis.^2^

Hemodialysis can only be performed with a well-functioning vascular access. The ideal vascular access should allow cannulation using two needles, which can support a minimum blood flow of 300 mL/min through a machine that serves as an artificial kidney, be resistant to infection and thrombosis, and have minimal adverse effects.^1^ Hemodialysis can be performed using a short-term catheter, a long-term catheter, an autologous arteriovenous fistula (AVF), or an arteriovenous graft (AVG). An AVF is the vascular access of choice, as several studies have shown that it is associated with lower rates of postoperative complications, lower maintenance costs, and fewer surgical or endovascular revisions to maintain patency compared to other modes of access.^3-6^ In addition, the use of short- and long-term catheters results in increased morbidity and mortality rates compared to native and prosthetic fistula. The risk of access-related hospitalization, death, and particularly of infection are much higher in patients undergoing hemodialysis with a short- or long-term central venous catheter.^7^ Most patients with stage 5 chronic kidney disease in Brazil requiring dialysis therapy would benefit immensely from having a well-functioning AVF or AVG.

Since making, managing and salvaging vascular accesses falls within the purview of the vascular surgeon, developing guideline to help specialists better manage vascular accesses for hemodialysis is of great importance. Thus, the objective of this guideline is to present a set of recommendations to guide decisions involved in the referral, evaluation, choice, surveillance and management of complications of vascular accesses for hemodialysis.

## METHODS

The work group chosen to compile this guideline consists of 14 vascular surgeons with extensive experience in vascular accesses for hemodialysis and significant work in their respective regional chapters. Initially, each member of the group formulated 10 questions relevant for their usual clinical practice and related to vascular accesses. After eliminating redundant questions, the members chose the 14 most relevant questions. Each question was answered by a member of the group, considering the best scientific evidence available from articles published in English and Portuguese language periodicals.

As reference for its research, the work group used reference databases such as MEDLINE, SciELO Brasil, PubMed, Embase, LILACS, and the Cochrane library. The research included articles published between January 1995 and May 2022. After writing their answers, these were reviewed and discussed by the work group in online meetings, culminating with the final version for each recommendation. The level of evidence for each answer was classified using the *Grading of Recommendations Assessment, Development and Evaluation* (GRADE) scale^8-10^ (Tables 1 and 2). When the evidence was not sufficient to classify an answer using the GRADE scale, the work group's opinion prevailed, and it was classified was “expert opinion.” The questions chosen by the work group were:

**Table 1:** GRADE Scale: Quality of evidence.^8-10^

**A - High**	There is a lot of confidence that the true effect lies close to that of the estimated effect.	**B - Moderate**	There is moderate confidence in the estimated effect. The true effect is likely to be close to the estimated effect, but there is a possibility that it is substantially different.
**C - Low**	There is limited confidence in the estimated effect. The true effect might be substantially different from the estimated effect.	**D - Very low**	There is very little confidence in the estimated effect. The true effect is likely to be substantially different from the estimated effect.

**Table 2:** GRADE Scale: Strength of recommendation.^8-10^

**1. Strong recommendation**	Desirable effects of treatment clearly outweigh the undesirable effects.
**2. Weak recommendation**	There is a smaller difference between desirable effects and adverse effects.

- Is preoperative mapping mandatory before creating an AVF?
- Is there an optimum site for long term access for hemodialysis?
- Is the use of imaging mandatory for long-term catheter implantation for hemodialysis?
- Is a native AVF the first option for vascular access for hemodialysis?
- Can a dysfunctional long-term catheter be salvaged?
- Is removing a long-term catheter in the presence of infection mandatory?
- Is there an optimal minimal vessel diameter for the creation of a vascular access for hemodialysis?
- Is there an optimal maturation period for AVFs?
- Are routine clinical examinations recommended for access surveillance?
- Is there a standard treatment for vascular access-induced ischemia?
- Should one treat AVF or AVG-related asymptomatic stenoses?
- Is there a preferred mode of anesthesia for AVF creation?
- In the presence of infection at AVF or AVG, is deactivation indicated?
- In the presence of an asymptomatic aneurysm, is surgical treatment indicated?

### Questions

#### **Question 1** - Is preoperative mapping mandatory before creating an AVF for hemodialysis?

No. Despite all efforts to identify methods to lower primary failure rates for autogenous AVFs, there is no consensus in existing studies regarding the effectiveness of preoperative vascular mapping (level of evidence 2C).

##### Justification

Functional native AVFs are considered the vascular access of choice for hemodialysis.^11,12^ However, creating a functional AVF in dialysis patients can be challenging, and primary failure rates range from 23 to 46 percent.^13^ Physical examination is traditionally used to identify suitable vessels for an AVF.^14^ Some authors recommend preoperative vascular mapping using a Doppler ultrasound in order to decrease primary failure rates.^15,16^

A systematic review published by Georgiadis et al.^17^ found a lower risk of primary failure among patients in the preoperative mapping group (OR = 0.32, 95% CI 0.17-0.6; p < 0.01). The authors conclude that preoperative mapping should be performed for all patients before AVF creation.^17^ However, in a Cochrane review involving 450 patients, Kosa et al.^18^ conclude that preoperative vascular mapping did not change AVF maturation rates. There was no significant difference: in the number of AVFs created successfully (RR = 1.06, 95% CI 0.95-1.28); in the number of mature AVFs after 6 months (RR = 1.11, 95% CI 0.98-1.25); in the number of AVFs used for hemodialysis (RR = 1.12, 95% CI 0.99-1.28); in the use of preoperative mapping compared to physical examination alone.^18^

Considering the conflicting results, lack of high-quality scientific evidence, the potential delay in creating the access, and the increased cost of mandatory preoperative examinations, especially in public health systems, we have established that for patients with reliable physical examination and low risk of AVF failure, preoperative vascular mapping is not mandatory. It is important to note that for patients at high risk of AVF failure (the elderly, women, peripheral occlusive atherosclerotic disease, coronary artery disease, the obese, children, patients with a history of multiple accesses) or inconclusive physical examination, preoperative mapping is indicated in order to improve the results for AVF creation.^19^

When assessing the central venous zone, a Doppler ultrasound (DUS) may not be a reliable exam. In patients with a history of multiple venous catheters and high likelihood of central occlusive venous disease, diagnostic venography should be considered.^12,20,21^

#### **Question 2** - Is there an optimum site for long term access for hemodialysis?

**Yes.** The choice of implantation site for a long-term central venous catheter (CVC) should be individually assessed, with the aim of optimizing access options, and performed after careful consideration of various factors, such as the need for emergency dialysis, life expectancy, potential for creating a native or prosthetic fistula, likelihood of fistula maturation, expected catheter removal, possibility of kidney transplantation, and patient choice. The following points should be considered when choosing a puncture site for a long-term catheter:

- upper limb before lower limb only if options are equivalent;
- if access via AVF or AVG is expected in the near future, give preference to a tunneled catheter in the opposite extremity to the intended AVF or AVG;
- if there is expectation of a kidney transplant in the near future, give preference to an internal jugular vein tunneled catheter (to salvage the iliac veins);
- the right internal jugular vein is recommended as the first choice for CVC implantation;
- avoid access via the subclavian veins for patients who will undergo AVF creation to the increased risk of central occlusive venous disease;
- some specialists believe that in emergency hemodialysis situations, in some circumstances (early removal of catheter) and when transplantation is not an option, femoral vein catheterization is acceptable (as long as there are no contraindications) until a fistula can be created or a peritoneal dialysis catheter can be used. The use of the femoral vein saves upper body blood vessels for a future fistula.

Contraindications for femoral catheters include femoral or iliac disease, prior surgery or reconstruction, hygiene (such as chronic diarrhea), morbid obesity (body mass index [BMI] greater than 35) and other difficulties in venous access.

When there are reasons for using a catheter and the estimated duration is long (greater than 3 months), but use of a fistula is not expected, the CVC should be positioned at the following sites, in order of preference:

- internal jugular vein;
- external jugular vein;
- femoral vein;
- subclavian vein;
- translumbar (insertion in the inferior vena cava with the tip in the right atrium).
  Note: In the absence of contraindications, previous disease (e.g., central stenosis, pacemaker), insertion of CVC on the right side is preferred over insertion on the left side because of its more linear anatomy. If disease on one side limits the establishment of an AVF, but still allows the catheter to pass, that side should be used, thus saving the opposite side for a future definitive access (level of evidence — expert opinion).

##### Justification

Historically, long-term CVCs are preferably implanted in the following order: internal jugular veins; femoral veins; and subclavian veins. In exceptional circumstances or when traditional puncture sites are unavailable, the external jugular vein, inferior vena cava, suprahepatic vein, renal veins, gonadal veins, popliteal veins, saphenous veins, and cervical, inguinal or pelvic collateral veins.^21-25^ The right internal jugular vein is generally considered the access site of choice due to lower complication rates compared to other puncture sites and to the left internal jugular vein. In a retrospective analysis published by Engstrom et al.^26^ including 409 participants and 532 catheters, the catheters implanted in the left jugular vein were at higher risk of infection-related removal compared to catheters inserted on the right side (0.33 *versus* 0.24 per 100 catheter-days; p = 0.012). Catheters implanted on the left side were also at higher risk of exchange due to dysfunction(0.13 *versus* 0.08 per 100 catheter-days; p = 0.08); however, the difference was not statistically significant. These results were modified based on CVC tip position. For CVCs positioned at the superior vena cava or at the cavoatrial junction, CVC dysfunction and infection rates were higher for implants on the left side. However, for CVCs with tips positioned in the middle of the right atrium, CVC dysfunction and infection rates for the left and right sides were similar.

**Figure 1:**
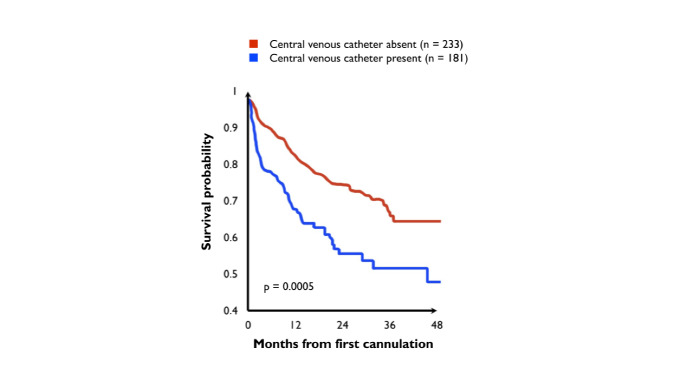
Kaplan-Meier analysis showing relationship between presence of central venous catheter and diminished access survival.^28^

**Figure 2:**
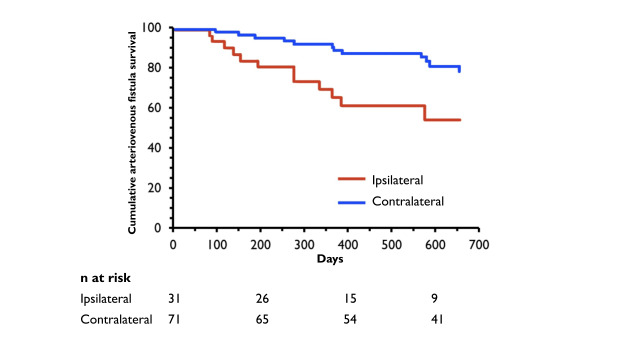
Analysis of survival of arteriovenous fistulae with presence of ipsilateral and contralateral central venous catheter.^29^

Studies have shown inferior arteriovenous fistula survival and maturation rates for patients undergoing dialysis by catheter, especially when it is inserted ipsilaterally to the fistula^19,27-29^ (Figures 1 and 2). Some authors have suggested that a long-term catheter should only be inserted in the subclavian vein of a hemodialysis patient when there are no other puncture sites due to the high risk of central venous stenosis.^23,30,31^ In the literature, the incidence of subclavian vein stenosis associated with catheter implantation ranges from 42 to 50%. In contrast, brachiocephalic vein stenosis associated with internal jugular vein catheter implantation ranges from 0 to 10%.^23,32^ Therefore, when using an arm for fistula creation, one should avoid inserting a catheter in the jugular vein and especially the ipsilateral subclavian vein.

The internal jugular vein is the most frequently chosen vein for CVC implantation due to easy access and lower complication rates.^33-35^ Many authors consider the common femoral vein as the second choice when implantation in jugular veins isn't possible, but the alternative is controversial due to patients’ anatomical and functional characteristics. Catheters inserted in the femoral vein have lower patency rates (44% per month) and higher infection rates (63/1,000 catheter-days)^30,36^ compared to accesses inserted in jugular veins. In a few specific situations, catheter implantation in the femoral vein may be the first choice. In patients requiring emergency dialysis who are not candidates for kidney transplantation and for whom early creation of vascular access for use over 30 to 60 days is expected, there may be some benefit to implanting the access in the femoral vein as opposed to the jugular vein, which is the usual site. A few advantages could justify this strategy as a reasonable strategy. First, implanting an access via the femoral vein would save the superior axis from endothelial damage and elevated risk of central venous stenosis. In addition, the presence of a catheter in their femoral vein may make more patients aware of the need for a fistula. It is important to emphasize that some systematic reviews failed at showing decreased risk of complications, such as thrombosis and catheter-related infections of the jugular vein, compared to accesses implanted in the femoral vein.^32,37,38^ Despite these facts, access via the jugular veins should be considered the first choice, with insertion via the femoral vein requiring caution and used only in specific situations.^1,19,39^

#### **Question 3** - Is the use of imaging mandatory for long-term catheter implantation for hemodialysis?

Yes. Long-term catheter implantation for hemodialysis in central veins should be performed at centers of excellence, by medical professionals, and guided by ultrasound and radioscopy (level of evidence 1B).

Central venipuncture for long-term catheter implantation should be performed under ultrasound guidance, minimizing catheterism-related complication risks (level of evidence 1A).

Radioscopy is the method of choice for adequate implantation and positioning of the tip of long-term catheters for hemodialysis and should be used whenever possible (level of evidence 1B).

If radioscopy is unavailable for the implantation of a long-term catheter for hemodialysis, another diagnosis method should be used after implantation to verify the catheter tip was positioned correctly, with conventional radiography the most frequently used method (level of evidence - expert opinion).

##### Justification

Immediate dysfunction of central venous catheters for hemodialysis (HD-CVCs) can be defined as an average flow below 300 mL/minute or the inability to complete a hemodialysis session due to inadequate flow.^39^ Therefore, successful implantation and correct positioning of HD-CVCs are critical for adequate renal replacement therapy, and imaging methods have a key role in ensuring excellence for both.

HD-CVCs may be inserted using anatomical landmarks or aided by ultrasound imaging. Ultrasound-guided insertion decreases early and late complication rates, in addition to optimizing the experience and satisfaction of dialysis patients.^40,41^ In a Cochrane meta-analysis by Rabindranath et al.^42^ which analyzed 7 randomized controlled trials and included 767 patients who underwent catheter insertion in their jugular or femoral veins, the authors concluded that ultrasound use significantly decreased the risk of catheter placement failure (RR = 0.40, 95% CI 0.3-0.52), the risk of arterial puncture (RR = 0.13, 95% CI 0.04-0.37), and hematomas (RR = 0.22, 95% CI 0.06-0.81) compared to the landmark method. Ultrasound use also significantly decreased catheter placement time and the number of attempts to successful insertion. There was no difference between ultrasound-guided placement and the landmark method for pneumothorax or hemothorax risk (RR = 0.23, 95% CI 0.04-1.38).

Other authors report the use of ultrasound guidance for HD-CVC placement helped significantly decrease early complications, such as hematomas, pneumothorax, and inadvertent arterial puncture, in addition to decreasing late complication rates, such as catheter malpositioning, vascular puncture, and thrombosis. Another significant factor recommending the use of ultrasound for deep venous access is the significant increase in successful HD-CVC insertion rates with ultrasound guidance.^19,30,40,43-49^ Therefore, HD-CVC placement using only the landmark method is restricted to situations where ultrasound use is impossible or unavailable. Correct positioning of the tip of long-term catheters for hemodialysis using radioscopy is currently the most accurate method, and increasingly recommended.^40,42,49-53^

Radioscopy should be used whenever available, both for placement via the jugular veins and the femoral veins, in order to position the tip of the long-term catheter for hemodialysis in the middle of the right atrium (for access via the internal jugular vein) and in central (non-distal) position in the inferior vena cava (for access via the femoral vein), thus preventing complications and inadequate flow.^19,40,42,49,52^ An observational study by Yevzlin et al.^54^ comparing the outcomes of fluoroscopically guided versus traditional placement of hemodialysis catheters found that catheter placement using the traditional method had lower rates of immediate success (OR = 0.12, CI = 0.02-0.71). In the absence of radioscopy, another imaging method should be used after implantation of long-term catheters to assess the correct positioning of the catheter tip, both in the thorax and the abdomen, with teleradiography being the most frequently recommended method.^40,42,49,51^

In short, the use of imaging methods for long-term catheter placement makes the procedure safer and more effective, and should be the method of choice in centers of excellence where these methods and the trained staff to perform them are available.^49,55,56^ The association between ultrasound for venous cannulation and the use of radioscopy to correctly position the long-term catheter significantly decreases major complication rates and early malfunction.^52^

#### **Question 4** - Is a native AVF the first option for vascular access for hemodialysis?

Not always. Choices must be made on an individual basis for each patient, and decisions based on a wide range of factors, such as life expectancy, probability of AVF maturation, patient choice, time to onset of dialysis, comorbidities, frailty scale, time to catheter removal, complication risks, access management plan, and assessment by multidisciplinary team (level of evidence 2C).

##### Justification

Ever since the concept of AVF as permanent vascular access was developed by Brescia, Cimino, Appel and Hurwich in the 1960s, the number of dialysis patients has increased exponentially with progressive technological advancements in renal replacement therapy, leading to a mismatch between demand and the capacity to provide vascular access. This established the ideal conditions for advancements in the use of prosthetic grafts, especially the development of expanded polytetrafluoroethylene (PTFE) by W.L. Gore & Associates in the 1970s, and the use of semi-tunneled catheters (STCs), which also grew significantly during the 1980s. The outcome was a significant increase in prosthetic fistulas and STCs and a decrease in the number of native AVFs, leading to high costs and requiring hospitalizations to manage these patients. This first period in the history of vascular access can be thought of as “graft and catheter first”.^57-59^

The biggest problem with dialysis catheters is infection, which is not a matter of possibility (“if it happens”), but rather timing (“when it happens”). Infection rates range from 2.5 to 5.5 cases/1,000 patient-days, or 0.9 to 2.0 episodes/patient-year, and the risk is 40 percent higher for temporary catheters compared to long-term ones. According to Allon et al.,^60^ the risk of bacteremia is proportional to how long patients depend on the device, reaching 35 percent in 3 months, 54 percent in 6 months, and 79 percent in 12 months in a sample of 472 hemodialysis patients with catheters. An aggravating factor is the immunologic impairment associated with chronic kidney disease, which predisposes patients to sepsis, significantly increasing the risk of death (5 to 9 times). According to estimates, severe complications occur in 10 percent of catheter-related bacteremia (CRB) cases, such as endocarditis, meningitis, septic arthritis, spondylodiscitis, septic shock, and eventually death. The relative risk of death associated with the use of catheters as permanent vascular accesses compared to native AVFs is 1.4 to 3.4 times higher. In addition, hospitalization rates increase, both for sepsis and in general, resulting in a higher annual cost for this patient group (Medicare data indicates annual average costs are USD 20,000 higher compared to fistula patients, with most of that increase coming from hospitalizations). No less severe, with a significant impact on quality of life and the ability to establish an adequate permanent vascular access on the limb, is central stenosis. There is a direct relationship between device type, central vein, and catheter permanence time, and risk of lesions in subclavian veins associated with temporary catheters can be as high as 50 percent. Refractory central venous stenoses may be treated with stent implantation, but outcomes are poor, with annual primary patency estimated at 14 to 25 percent.^3,57-80^

The concept of *fistula first* began with the *Fistula First Breakthrough Initiative* program in 2003, in the U.S., with the primary goal of changing medical practice related to dialysis accesses. The initiative was created based on data from the Dialysis Outcomes and Practice Patterns Study (DOPPS) and clinical performance measurements (CPM) developed by the U.S. government's Centers for Medicare and Medicaid Services. The concept was also developed from consensus guidelines on vascular access published in 1997 by the National Kidney Foundation, in a document titled Kidney Disease Outcomes Quality Initiative (KDOQI) - Clinical Practice Guidelines for Vascular Access. In the 1990s, vascular accesses were the main cause of morbidity and mortality, with dialysis access failure the primary cause of hospitalizations and their complications accounting for approximately 14 percent of the total cost of end-stage chronic kidney disease (USD 1 billion per year at the time). During that period, there was a large amount of prosthetic fistulas and STCs and a small percentage of native AVFs, resulting in a large number of hospitalizations, secondary interventions, and high costs.^81-85^

DOPPS is an international observational prospective study with the goal of analyzing the relationship between various dialysis practices and patient outcomes. From its beginnings in the U.S. in 1996, it now includes multiple countries from almost every continent, resulting in multiple publications, mortality data for over 90,000 patients, and detailed follow-up for over 30,000 patients. An analysis of publications on the subject of dialysis access finds that: (1) native AVFs represent the best option for vascular access, with fewer complications and less need for interventions, and should be considered the access of choice; (2) catheter use is associated with increased mortality risk, increased risk of hospitalization, and worse anemia control; (3) dialysis units with higher rates of native AVFs are dedicated to having them as the access of choice; (4) surgeon experience is a key factor for vascular access management.^86-94^

Among patients referred to native AVF creation, only about 50 to 75 percent are eligible, with 40 to 50 percent eligible for distal fistulas and approximately 25 to 35 percent eligible for proximal fistulas. The problem with native AVFs is primary failure, which may be cause by early thrombosis (≤ 6 weeks) or maturation failure. The maturation process is complex; simply put, it is the consequence from the interplay between neointimal hyperplasia (negative remodeling) and vasodilation (positive remodeling). In the Dialysis Access Consortium multicenter trial, Dember et al.^95^ reported early thrombosis in 25 percent of distal fistulas and 13 percent of proximal fistulas, in addition to failed maturation rates of 64 versus 53 percent within 6 months. The data makes it clear how challenging creating a functional native AVF can be. A natural consequence of standardizing native AVFs as the access of choice in all situations was a significant increase in maturation failure, probably due to the higher number of attempts in marginal veins and patients, fostering a considerable increase in maturation interventions which translate into significant costs and longer dependence on catheters. In 2007, Wasse et al.^96^ reported greater dependence on catheters 90 days after dialysis therapy initiation (60 percent) compared to the previous decade (40 percent), associated with lower conversion to prosthetic fistulas (25 versus 40 percent), implying extended use of catheters as transition access before fistula maturation. In 2013, Lok et al.^97^ published a review of 10 years worth of data from US Renal Data System, from 2000 to 2010, totaling 1,740 accesses, and found failure rates for AVFs were twice as high as those for prosthetic grafts (39.7% versus 18.8%, p < 0.001). A meta-analysis reported an average AVF maturation time of 3.4 months, with unused access abandonment in up to 20 percent of cases. In analysis of the efficiency of that mode of access, Ladak et al.^98^ observed that for patients who underwent AVF creation exclusively, only 57 percent achieved catheter independence, and only 40 percent of hemodialysis is catheter-free.^95-104^

As a consequence of this unexpected scenario, caused by the *fistula first* standardized model of access management, may nephrologists have begun to challenge the actual benefits of the notion that “native AVFs should always be the first options,” leading to a new dialysis access management paradigm known as “fistula not so first, but catheter always last.” The major difference in this new philosophy is a critical analysis of the positive and negative aspects of native AVFs and prosthetic fistulas, putting the latter back at center stage as an adequate access option and seeking to consider the specifics of situations providing ideal conditions for each access type. This can be explained by the outcomes of comparative studies analyzing intention to treat, which found a higher primary failure rate for native AVFs (32 to 40 percent) compared to prosthetic fistulas (12 to 19 percent) and similar secondary patency, despite obvious differences in terms of infection and intervention rates. According to this new paradigm, the primary goal becomes reducing catheter dependence, regardless of whether the permanent access is a native or prosthetic fistula. The criteria for choosing the best access would be: (i) initiation of dialysis treatment; (ii) patient's average life expectancy; (iii) likelihood of primary maturation failure; and (iv) prior maturation failure. There is a clear effort to consider the specifics of the patient-access pair, optimizing the advantages of each access type for each patient situation. When considering the two extremes, we are left with two unassailable situations: (1) native AVF as access of choice for younger patients, with low probability of non-maturation (male, nondiabetic, preemptive access and adequate ultrasound mapping); and (2) prosthetic fistula for elderly nonfrail patients with low life expectancy and high probability of non-maturation (female, diabetic, prior maturation failure of native AVF).^97,105-111^

Finally, it is important to analyze STCs. The elderly have been the subject of extensive analyses and, therefore, a significant number of publications. Considering specificities for this extremely heterogeneous group is only natural. On the one hand, we have nonfrail, nondiabetic elderly patients with good life expectancy; on the other, frail elderly patients with multiple cardiovascular comorbidities and low life expectancy. Within this context, a more detailed analysis of those publications and the data inherent to dialysis catheters is required. In 2020, De Clerck et al.^111^ published a retrospective study correlating access type and mortality. Unlike their peers, they performed a longitudinal analysis over 11 years and managed to obtain data both on the incident vascular access and on the impact of changing vascular access type over time. When analyzing only the group of patients who kept the same access type over the long term, there was no statistically significant difference between fistula and catheter patients. When mortality was compared based on vascular access as a variable that changes over time, there was a 39 percent decrease in the fistula group (OR = 0.61, p = 0.005, 95% CI 0.44-0.87). However, in the multivariate analysis the difference between access and catheter groups there was no statistically significant difference (OR = 0.92, p = 0.722, 95% CI 0.58-1.46), and age, history of heart failure and cancer were the only significant parameters. In 2017, Ravani et al.^112^ analyzed DOPPS data for the period between 1996 and 2011 related to access type, access complications and mortality and concluded that the complications inherent to access types are unable to explain different mortality rates. In 2020, Ko et al.^113^ showed that in among octogenarians starting dialysis with catheters and converting to native AVFs within the first years, mortality was comparable to that of patients who began their treatment with AVFs and outcomes were better than for patients retaining catheters as permanent vascular access.^19,112-115^

In 2020, Lok et al.^19^ published an update to the KDOQI Vascular Access Guideline, highlighting the importance of considering the needs of individual patients in relation to vascular access. We can summarize the current paradigm in dialysis access management as “fistula not so first and catheter not so last”.^19^ Advances in recent decades have taught us that each access type has its benefits and advantages, and the greatest challenge for the multidisciplinary team treating the patient suffering from chronic kidney disease is to develop a management plan to optimize the advantages of each access type in their particular circumstances, taking into account considerable ethnic, social, economic and cultural differences between continents. We can summarize the current philosophy as the optimal access for an optimal patient under optimal circumstances.

#### **Question 5** - Can a dysfunctional long-term catheter be salvaged?

Yes. In case of kinks in the path, these must be resolved with the aid of a rigid guidewire or surgical revision or recreation of the tunnel, with or without exchanging the catheter (level of evidence 1B).

If the catheter tip is malpositioned, reposition or exchange the catheter with the aid of a rigid guidewire. The catheter should be exchanged for a longer one (level of evidence 1B).

Placing the patient in Trendelenburg position and/or using a saline infusion may salvage catheter function in some cases. The maneuver should be attempted especially when an obvious mechanical cause for the dysfunction has not been identified (level of evidence 1C).

In case of late catheter dysfunction, extrinsic thrombosis should be ruled out (level of evidence 1B).

Intrinsic thrombosis should be treated with the infusion of thrombolytic agents in both routes. The procedure should be attempted 1 or 2 times. Alteplase 2 mg in each route for 30 to 60 min is the therapy of choice (level of evidence 1A).

If fibrin sheath is suspected, exchanging the catheter for a rigid guidewire is required (level of evidence 1A).

There is no data to justify or contraindicate rupturing the fibrin sheath with a balloon catheter before replacement with a new catheter. Therefore, this decision should be made at an individual level (level of evidence 2B).

Early recurrence of dysfunction after initially successful infusion of thrombolytics is very often caused by the presence of fibrin sheaths (level of evidence 1B).

Acute catheter-related thrombosis of the superior vena cava/right atrium/central veins—extrinsic thrombosis— should be treated with full anticoagulation. Initial drug therapy should be a continuous infusion of unfractionated heparin (level of evidence 1A).

##### Justification

Hemodialysis catheter dysfunction is found when the catheter does not enable the performance of hemodialysis in the first 60 minutes of a session after at least one attempt to improve the flow. Dysfunction is suspected when flow through the catheter is lower than 300 mL/min.^116^

Additional suspicious findings include lower Kt/V, presence of blood pressure below 250 mmHg and/or venous pressure above 250 mmHg. Access conductance, measured in Qb/Pa, is the ratio between pump flow (Qb) and negative device pressure (Pa). Its normal value is 2 mL/min/mmHg. The need for greater negative pressures to keep the same flow is also a warning sign.^117^

Despite these definitions, KDOQI stresses that many patients, especially those with body mass under 70 kg or those undergoing long-term dialysis, dialyze at flows below 300 mL/min without that becoming an issue. In addition, many catheters present temporary or intermittent dysfunction, but work normally in subsequent sessions. Thus, more objective criteria to define dysfunction are required.^19^

Dysfunction is classified as immediate or late (delayed). Immediate dysfunction happens upon first use after implantation. It is usually caused by poorly positioned catheter tips or kinks in the path. Ideally, the catheter tip should be positioned in the right atrium. When placed in the superior vena cava, it may disrupt adequate blood flow. This is more frequent with obese patients, since the catheter tip can move subcutaneously when patients are in a standing position, and for left central venipuncture. A simple x-ray can rule out kinks and assess the position of the catheter tip; in questionable cases, intravenous contrast injection through the catheter under fluoroscopy enables one to determine the position of the catheter tip and the right atrium. Saline solution injection and blood aspiration are both maneuvers to verify patency.^19^

Late dysfunction is a consequence of fibrin sheath formation, possibly the most frequent cause, or thrombus formation, whether on the catheter tip, the vein where it was placed or in its lumen.

Thromboses were classified as extrinsic when the thrombus was found on the vessel wall and outside the catheter, and as intrinsic when the thrombus occupied the lumen or adhered to the surface.^118^

Extrinsic thrombosis thus represents deep vein thrombosis of the superior vena cava, right atrium, brachiocephalic vein(s)—in short and malpositioned catheters—and/or the inferior vena cava. Clinical presentation varies and can lead to symptoms of superior vena cava syndrome or even pulmonary embolism, with their well-known consequences. There is no specific data on treatment for acute superior vena cava or right atrium thrombosis, which may or may not be catheter-related. In general, they are treated like other deep vein thromboses, and full anticoagulation is indicated. Continuous infusion of unfractionated heparin is the drug of choice because these are patients with severe renal dysfunction, which restricts the use of low-molecular-weight heparin, and planning for brief surgical procedures. Catheter removal is not mandatory, but often necessary, and may be suggested in the presence of catheter-related infection or unsatisfactory clinical evolution despite adequate anticoagulation. The exchange should be made using a rigid guidewire in order to save the pathway for a new catheter. In the presence of infection, parenteral antibiotics should be administered.^119-121^

Intrinsic thrombosis may be caused by fibrin sheath formation, which is the most frequent cause, thrombus on the catheter tip or thrombus in the lumen. It is associated with decreased or absent flow through one or both catheter routes.

Fibrin sheath formation begins in the first 24 hours after catheter implantation in response to the injury to the blood vessel. After a few days, it extends to its entire length and becomes the primary cause of late dysfunction. It usually manifests several days after insertion but can be evident as soon as the first day.

The use of thrombolytic agents has high success rates for recanalization of occluded catheters, of more than 80 percent. In addition, it increases the number of days the catheter can be used before it has to be exchanged. In Brazil, alteplase is the most widely used drug, at a dose of 1 to 2 mg per route. The thrombolytic agent is usually injected in both routes. It remains in the catheter for 30 to 60 minutes before aspiration and one can attempt to reestablish the flow. If the first infusion fails, a second attempt is made, leaving the thrombolytic agent for an additional 30 to 90 minutes, or even until the next hemodialysis session. There is no data to prove the superiority of one particular type of thrombolytic, dosage or administration route.^122^

Recanalization of catheters with fibrin sheaths usually fails after infusion of thrombolytic agents, or present early recurrence after initial success. Fibrin sheath diagnosis is made using angiography, performed using the dysfunctional catheter itself, after withdrawing it a few centimeters out of the skin. In these patients, one alternative is fibrin sheath rupture/disruption using an angioplasty balloon catheter, where a rigid guidewire is passed through the catheter, similar to an exchange using a standard guidewire. After catheter removal, a full balloon catheter angioplasty is performed. Next, a new catheter is placed in the standard manner. However, there is no data to attest the superiority of fibrin sheath rupture over a simple catheter exchange using a guidewire.

In terms of access dysfunction, there is no literature thus far to justify the use of other substances—thrombolytic agent, citrate, among others—besides heparin to decrease catheter dysfunction.^123^

#### **Question 6** - Is removing a long-term catheter in the presence of infection mandatory?

No. Management options for catheter-related infections in long-term catheters are exchanging the catheter for guidewire, exchanging the catheter with the creation of a new tunnel, removal of catheter, and salvage of catheter with systemic antibiotic therapy. The decision between the four options depends on patient hemodynamics, presence of sepsis, persistent bacteremia, the specific microorganism isolated in the cultures, signs of tunnel infection, and vascular access failure.

##### Recommendations

1. When CRB is suspected and before administering empirical antibiotic therapy, collecting two blood samples for peripheral blood cultures is recommended. If the decision is made to salvage the catheter, simultaneous collection of blood samples from the lumen of the tunneled venous catheter (TVC) and from a peripheral vein is also recommended. For diagnosis, the samples should be cultured using a quantitative technique, or calculating the differential time to positivity for the two (level of evidence 1B).
2. Removal of the TVC is recommended in case of complicated local infection (tunnelitis), complicated systemic infection (septic shock, persistent fever or positive blood culture 72 hours after initiation of adequate antibiotic treatment, septic embolizations such as endocarditis, thrombophlebitis or spondylodiscitis), or when the patient has other intravascular prosthetic implants (pacemakers, endografts, valves, etc.) (level of evidence 2B).
3. When CRB is suspected, broad spectrum systemic empirical antibiotic therapy is recommended before microbiological results are available (level of evidence 1A).
4. The initial recommendation is simultaneous systemic antibiotic therapy and sealing TVC lumens with antibiotics in uncomplicated catheter-related bacteremia (level of evidence 2B).
5. After removing an infected TVC, a new one should be placed after establishing adequate antibiotic treatment and obtaining negative control blood cultures. If possible, the new catheter should be placed at a different site from the previous one (level of evidence 2A).
6. TVC removal is recommended for catheter-related bacteremia featuring virulent microorganisms such as *Staphylococcus aureus*, *Pseudomonas spp*., *Candida spp*. or multidrug-resistant microorganisms (level of evidence 1B).

##### Justification

Dialysis catheter-related infection (CRI) is the most frequent and most severe complication for TVCs, associated with high morbidity and mortality.^72^ CRB incidence ranges from 2.5 to 5 episodes per 100 catheter-days, corresponding to an incidence of 0.9 to 2 CRB episodes per year.^60,62,66,124^ In patients with TVCs, risk of bacteremia is 10 times greater than in patients with native AVFs.^125-127^ In the 2013 article by Shingarev et al.,^68^ assessing the natural history of 472 TVCs, median time to TVC-related bacteremia was 163 days, with 35 percent of patients infected within 3 months, 54 percent within 6 months, and 79 percent within 12 months.

The most frequent clinical characteristics of TVC infection include fever or chills, hemodynamic instability, catheter dysfunction, hypothermia, nausea and vomiting, and general malaise.^63,128,129^ Dialysis CRI may lead to severe complications, such as osteomyelitis, endocarditis, thrombophlebitis and death in 5 to 10 percent of patients.^60,130,131^ Severe septic embolizations happen more frequently in infections caused by *S. aureus*, one of the microorganisms most often isolated (10-40%).^132^

There are three types of catheter-related infection:^133,134^

- Uncomplicated local infection. Defined as the presence of inflammatory signs restricted to 2 cm around the exit orifice on the skin, without extending above the catheter cuff. It may or may not be associated with fever and bacteremia and may be accompanied by purulent exudate out of the exit orifice.
- Complicated local infection. Defined as the onset of signs of inflammation extending beyond 2 cm from the exit orifice and the catheter's subcutaneous pathway (tunnelitis). It may or may not be associated with fever and bacteremia and is accompanied by purulent exudate.
- Systemic infection or catheter-related bacteremia. Defined as isolation of the same microorganism in the blood and in the TVC in the absence of other sources of infection. Complicated systemic infection is characterized by septic shock, persistent fever and/or positive blood cultures 48 to 72 hours after initiation of adequate antibiotic therapy or embolic complications (endocarditis, thrombophlebitis or spondylodiscitis).

##### Diagnosis of CRB

The most sensitive clinical manifestations for CRB diagnosis, despite their low specificity, are fever and/or chills,^63,65,135^ while the presence of exudate or inflammatory signs at the TVC exit orifice are more specific, but significantly less sensitive. In most CRB cases, there is no evidence of infection of the entry site.^136^ Other, less frequent clinical manifestations are hemodynamic instability, changes in consciousness level, catheter dysfunction, and signs and symptoms of sepsis. Sometimes, complications from bacteremia (endocarditis, septic arthritis, osteomyelitis or abscesses) may be the first manifestation of CRB.

Clinical suspicion of CRB is warranted when a TVC patient presents with fever, chills and/or any suggestive clinical or hemodynamic changes. The suspicion is stronger if the episode is associated with handling or local inflammatory signs at the insertion site or the subcutaneous tunnel of the catheter. The episode should then be evaluated using the patient's clinical history and a basic physical examination to rule out other possible sources of infection besides the TVC.

Isolated clinical criteria are not sufficient to diagnose CRB, which involves clinical assessment and microbiological confirmation using blood cultures and/or catheter cultures. Reference diagnosis techniques are based on culturing the catheter tip after removal;^137-141^ next, CRB diagnosis is established by positive culture and isolation of the microorganism from the blood culture. An accurate diagnosis is important to avoid unnecessary removal of the TVC and the potential risks associated with placement in a different site. Likewise, one should consider that TVC removal is not always required for adequate diagnosis and treatment.^63,65,142-145^

Quantitative blood cultures obtained simultaneously through the catheter and direct collection from a peripheral vein (ratio of the number of colony-forming units [UFC/mL] from 3:1 to 10:1) are indicative of CRB, with 79 to 94 percent sensitivity and 94 to 100 percent specificity.^146-151^

Despite high specificity, this technique is not a routine method in most microbiology laboratories due to its cost and complexity. Since many hospitals have automatic devices for detection of microbial growth in blood samples, an alternative method to quantitative blood cultures has been proposed, measuring the differential time to positivity from blood cultures collected simultaneously from the TVC and by direct venipuncture. The basis for that technique is the fact that time to positivity for blood samples is directly related to the number of microorganisms initially present in the sample;^152^ therefore, when positivity of blood cultures collected via the TVC occur at least 2 hours before positivity for samples collected from peripheral venipuncture, there is differential time to positivity. Differential time has 94 percent sensitivity and 91 percent specificity for CRB diagnosis in patients with TVCs.^152,153^

If CRB is suspected and before administering antibiotics, venipuncture should be performed to obtain two blood samples from different sites or with 10 to 15 minutes between collections. After TVC removal, proceed to culturing the tip. When there is no indication for immediate removal of the TVC, blood samples are collected simultaneously through all catheter lumens and from a peripheral vein.

According to the KDOQI 2019 guideline,^19^ CRB diagnosis is defined as:

- suggestive clinical manifestations with at least 1 positive blood culture collected from the dialysis circuit or a peripheral vessel and no other apparent source, with semi-quantitative (> 15 UFC/catheter segment, either hub or tip) or quantitative positive culture (> 10^2^ UFC/catheter segment, either hub or tip), where the same organism (species and antibiogram) is isolated from the catheter segment and from a peripheral blood sample (dialysis circuit or vein). The following factors would strengthen the diagnosis: simultaneous quantitative cultures of blood samples with ≥3:1 catheter hub/tip x peripheral [dialysis circuit/vein]); differential time to positivity of at least 2 hours for catheter blood culture versus peripheral blood culture.
  In short, microbiological confirmation of CRB is established when:
- the same microorganism is isolated at the TVC tip and in a peripheral venous blood culture;
- the same microorganism is isolated in at least two blood cultures (one of the TVC lumens, the other from a peripheral vein) and diagnostic criteria for quantitative blood cultures are met or differential time to positivity is calculated.

If the microorganism isolated in a single blood culture is a coagulase-negative staphylococcus, new blood samples are required to rule out contamination. When a TVC is removed due to suspected CRB, the catheter tip should be cultured using quantitative or semi-quantitative techniques. Colonization is established when over 15 UFC/mL (Maki technique) or over 10^2^ UFC/mL (Cleri technique) are quantified during growth.^139-141,154^

##### Treatment of catheter-related infection

The most frequently isolated microorganisms in CRB are gram-positive bacteria. Coagulase-negative staphylococcus, alongside *S. aureus*, represent between 40 and 80 percent of cases, so initial treatment should be effective against these types of microorganisms while waiting for microbiological confirmation.^60,64,142,155^ Infection with *S. aureus* has been associated with high morbidity and mortality.^156-158^

Non-staphylococcal CRB is predominantly caused by enterococci, corynebacteria, and gram-negative bacilli. Infection by gram-negative bacteria have increased in recent years, and can represent as much as 30 to 40 percent in some centers.^60,64^

CRB treatment can consist of systemic antibiotic therapy or, on the other hand, TVC management in terms of removal or salvage. Therefore, once antibiotic treatment begins, one needs to decide among the following options:^134^

- Immediate removal
  - Complicated local infection.
  - Presence of septic shock.
  - Persistent fever or bacteremia 48 to 72 hours after initiation of antibiotics matching the sensitivity of microorganisms present.
  - Evidence of septic embolization (endocarditis, suppurative thrombus, phlebitis, spondylodiscitis, etc.).
  - Isolation of highly virulent pathogens: *S. aureus*, *Pseudomonas spp*., *Candida spp*. or multidrug-resistant microorganisms.

Once the infected TVC is removed, the best alternative is placing a new catheter, if possible in a different anatomic location. Though we currently lack sufficient evidence, we recommend implanting a new TVC once adequate antibiotic therapy has been established and negative control blood cultures have been obtained.

- Sealing catheter lumen with antibiotic solution

In uncomplicated CRB, a conservative treatment may be attempted, keeping the TVC in place. Previous experiences, where the catheter remained in place and systemic antibiotic treatment was administered intravenously (occasionally through the colonized catheter itself), found cure rates ranging from 32 to 74 percent, alongside high risk of recurrence when antibiotics are discontinued.^159-161^

Often in CRB, biofilms are formed that can occupy both the external surface and the intraluminal surface of the TVC.^162^ The microorganisms causing the infection are present in the biolayer on the internal catheter surface, which makes them resistant to antibiotics and explains the difficulty in eradicating infections from TVCs treated with intravenous antibiotics only.^163^

Sealing the lumen with antibiotics associated with concomitant systemic antibiotic therapy may be an alternative treatment strategy to salvage the catheter. Though there are no randomized controlled trials assessing the role of sealing the catheter with antibiotics in treating CRB, observational studies have shown that bacteremia can be eradicated with antibiotic blocks combined with systemic antibiotics compared to exchanging or removing the catheter combined with systemic antibiotics.^60,128,134^

Treatment should be performed simultaneously, preferably using the same antimicrobial agent both systemically and locally. Treatment duration will be the same as for systemic antibiotics (usually 2 to 3 weeks, depending on etiology). Rigorous patient follow-up is required to detect persistent fever, positive blood cultures 48 to 72 hours after initiation of antibiotic treatment corresponding to microbial sensitivity, onset of septic complications or recurrence of CRB. In these cases, TVC removal is indicated.

CRB treatment exclusively with systemic antibiotics, keeping the catheter but not sealing it, is insufficient to eradicate the microorganisms present in the biofilm and resolve most CRB cases, with high rates of recurrence.^163^

- Exchange of infected CVC with guidewire

Late removal of an infected TVC (when there is no indication for immediate removal or it was not possible to remove it at the moment) and exchanging for a new catheter using a guidewire is considered an acceptable alternative.

Exchange by guidewire has produced similar outcomes compared to immediate removal in several nonrandomized trials.^64,164-166^ The strategy should only be considered if the symptoms disappear quickly. Therefore, we recommend exchanging the catheter at least 48 to 72 hours after initiation of antibiotic therapy when the patient remains clinically stable and there is no evidence of subcutaneous tunnel infection.

When, after clinical improvement following initiation of antibiotic therapy, the catheter is exchanged using a guidewire, and later when blood culture positivity is confirmed, it seems prudent to collect additional samples for blood cultures in order to verify that bacteremia has resolved. If that has not occurred, the new catheter should also be removed.

In patients characterized by vascular access failure, the strategy of exchanging with a guidewire also seems acceptable, since creating a new access may be a complicated task.

CRI is a frequent condition in the natural history of TVCs, and their removal should be considered on a case-by-case basis, depending on the presence of sepsis, persistent bacteremia, virulence of the specific microorganism isolated in the cultures, signs of tunnel infection, and vascular access failure. Immediate removal of the TVC, without the application of rigorous criteria, may result in permanent loss of the access site and promote vascular access failure.

#### **Question 7**- Is there an optimal minimal vessel diameter for the creation of a vascular access for hemodialysis?

No. Despite countless reports on attempts at identifying the minimal acceptable vessel diameter for access creation, there is no consensus in existing studies. Current evidence does not enable us to recommend a minimal vessel diameter for AVFs (level of evidence 2C).

##### Justification

Autogenous AVFs for hemodialysis are preferred to other modes of vascular access, since they are associated with improved long-term primary patency rates and lower infection rates over synthetic grafts or catheters.^1,167^ AVFs have lower morbidity and mortality rates because they require less intervention over other types of access. In situations where predicting adequate venous flow using only clinical examinations is not possible, the use of arteries and veins below recommended vessels may have contributed to higher early failure rates for autogenous accesses.^167,168^ Each upper extremity has at least four potential sites for the creation of conventional access, using the cephalic or basilic veins in the forearm or upper arm, in one or multiple stages.^168^ When based only on physical examinations, many fistulas (28%-53%) never adequately mature and cannot be used in hemodialysis sessions.^169^

The combination of detailed physical examinations associated with preoperative duplex ultrasound provides valuable information that enable surgeons to choose the best combination of arterial and venous flow to successfully create an AVF.^167^ In addition, they are readily available, noninvasive and low cost and have high sensitivity to assess the quality of components involved in the creation of vascular accesses. In assessing the arterial system, segmental blood pressure and Doppler waveforms are observed, while venous vessels require exact measurements of vein diameter and determining the presence of sclerosis/stenosis of the superficial veins of the upper extremities. Arteries require diameters of at least 2 mm and blood flow of over 500 mL/min to enable adequate dialysis. Veins require at least 2.5 mm for a fistula and 4 mm for a synthetic graft.^1,167-169^

Therefore, throughout the years, the diameters of the blood vessels involved in the creation of autogenous accesses have been the subject of discussion. Considering the diameter and quality of the blood vessels involved before performing the vascular access procedure is thought to be reasonable. There is no consensus, even among the various guidelines.

KDOQI, for instance, admits that though there is no minimum-diameter threshold to create an AVF, arteries and veins of < 2 mm in diameter should undergo careful evaluation for feasibility and quality to create a functioning AVF.^19^ Likewise, it suggests evaluating multiple characteristics of vessel quality for AVF creation (size, distensibility, flow and wall thickness).^19^ The multidisciplinary group from the Spanish Society of Vascular Surgery recommends that arteries < 1.5 mm and veins < 1.6 mm in diameter be considered of dubious feasibility for access creation.^39^ Finally, the European consensus group emphasizes the need to consider an alternative site for AVF creation whenever ultrasound measurement of the inner radial arterial diameter is less than 2.0 mm and/or the cephalic venous diameter is less than 2.0 mm.^1^

The previously suggested venous diameter of 2.5 mm and arterial diameter of 2 mm were not validated by consistent studies over the years.^19,39^ Therefore, the threshold included in the KDOQI clinical practice guideline for vascular access considers few trials and reports, limited to retrospective studies from a single center assessing vessel diameter, and asks questions regarding the timing (immediate before surgery), distensibility with tourniquet, operator skills (technician versus surgeon), and location (radiocephalic versus brachiocephalic).^19^ The variability in reported parameters limits the clinical evidence necessary to make any recommendations on minimal venous and arterial lumen size.^19^

Some studies produce clinical evidence which, though limited, meet guideline review criteria and may provide relevant information, summarized below:

In 2001, Allon et al.^170^ conducted a 17-month study using routine preoperative ultrasound evaluation of upper limb arteries and veins to plan the arteriovenous fistula procedure. The types of access created and their long-term outcomes were compared to institutional historical controls placed on the basis of physical examination alone. Minimum vein diameter of > 2.5 mm and arterial diameter of > 2.0 mm for AVF creation and vein diameter > 4.0 mm for prosthetic fistula creation were used as parameters in the study. In general, compared to historical controls, the study found an increased AVF creation rate, from 34 to 64 percent, with higher rates of improvement for women and diabetes patients. The general increase in AVF usability for dialysis in the historical cohort was not statistically significant (46 to 54%; p = 0,34). However, there was a substantial increase in forearm AVF usability, though not a statistically significant one (34 to 54%; p = 0,06).^170^

In 2007, Parmar et al.^171^ assessed the impact of routine radial arterial duplex for imaging radial artery before AVF formation.^171^ Their purpose was to investigate the relationship between radial artery diameter and AVF patency. They performed duplex sonography before AVF formation 1 day, 1 week, 4 weeks and 12 weeks post AVF formation. Patients were divided into 2 groups: group 1, 11 patients with radial artery diameter < 1.5 mm; and group 2, 10 patients with radial artery internal diameter > 1.5 mm. In group 1, 5 patients (45%) showed immediate thrombosis of AVF. All patients in group 2 had patent AVF at 12 weeks. There was a high failure rate of AVF with radial artery < 1.5 mm. They concluded that in the presence of small radial arteries, primary access AVF in the upper arm should be considered.^171^

In 2013, Nica et al.^172^ reported that patient vessel diameter is an important factor when deciding eligibility for fistulas. They conducted a survey of international surgeons, using hypothetical patient scenarios, to assess possible perceived barriers and absolute contraindications to access creation. A total of 134 surgeons completed the survey. Increased comorbidities and previous failed access were deterrents to AVF creation as was vessel size. Overall, 70 percent of surgeons reported the need for minimum vein diameters of 2 to 3 mm for vascular access creation. They found that U.S. and European surgeons were more likely than Canadian surgeons to allow AVF creation in cephalic veins with only 1.5 to 1.9 mm in diameter. Likewise, European surgeons were more likely than their American and Canadian peers to use basilic veins with only 2 to 2.5 mm in diameter. They concluded that significant variability exists in the surgical preoperative assessment of patients, and the eligibility criteria used for fistula creation, as well as that understanding surgeons’ preferences can aid in establishing clearer standardization for access eligibility.^172^

**Figure 3:**
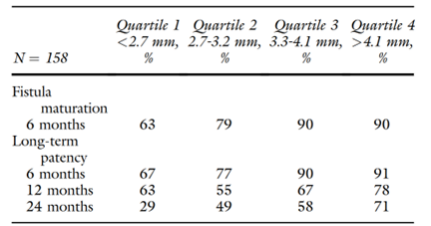
Maturation rates and primary patency according to vein diameter.^173^

More recently, Dageforde et al.^173^ conducted a cohort study where brachiobasilic or brachiocephalic AVF accesses were divided in quartiles by vein diameter: vein diameter in quartile 1 was < 2.7 mm, and > 4.1 mm in quartile 4.^173^ Patients with minimum vein diameter ≥ 3,3 mm had a higher maturation rate than those with vein diameter < 2.7 mm (90 versus 63 percent) and < 3.2 mm (90% versus 79%). Patients with minimum vein diameter < 2.7 mm had a non-maturation rate of approximately 40 percent. Multivariate Cox regression analysis found that for a 1 mm increase in vein diameter there was a 45 percent decrease in the risk of non-maturation and a 36 percent decrease in the risk of primary patency loss. Primary patency rates by vein diameter within 6, 12 and 24 months were 67, 63 and 29 percent for veins < 2.7 mm and 90, 67 and 58 percent for veins ≥ 3.3 mm (Figure 3). The authors concluded that smaller-diameter veins are associated with higher likelihood of non-maturation and loss of primary patency. However, in the absence of the clinical evidence required for turning it into a guideline, a minimum vein diameter for fistula creation could not be recommended.^173^

Therefore, over the years and based on expert opinions, minimum vein diameter > 2.5 mm and arterial diameter > 2.0 mm for AVF creation and vein diameter > 4.0 mm for vascular access graft creation were implemented and considered safe to ensure AVF maturation.^1,167-169,172-174^

#### **Question 8** - Is there an optimal maturation period for AVFs?

Yes. The optimal period ranges from 4 to 12 weeks, and most mature native AVFs can be punctured after 6 to 8 weeks (level of evidence 2C).

##### Justification

AVF maturation depends on adequate blood flow in order to prevent thrombosis and make hemodialysis viable. It requires increasing both arterial and venous blood flow. Average basal blood flow in the brachial artery is 31 mL/min, and it needs to increase from 10 to 20 times,^175^ accompanied by progressive vein dilation. Studies show that these adaptations begin immediately after completion of the anastomosis: within 10 minutes, radial arterial flow can increase from 20.9 mL/min to 174 mL/min.^176^ The vein dilates more quickly, becoming clinically evident.^84^ Preoperative vein and artery diameter and pulsatility index also influence maturation time and rates. Brachiocephalic fistulas are more likely to mature than radiocephalic fistulas.^177^

But what, exactly, is a properly mature fistula? There is no universal definition.^178^ The literature provides at least three different definitions of AVF maturation:

- Ultrasound maturation: criteria established by Doppler ultrasound. They are widely known as the “rule of 6s”: 600 mL/min flow, vein diameter of 6 mm and depth of 6 mm below the skin.^84^
- Clinical maturation: empirically, the capacity for AVF cannulation with 2 needles, with adequate blood flow for hemodialysis, for a period of 30 consecutive days.^179^
- Anatomic pathology maturation: the process of *arterialization* of the efferent vein. The vein becomes progressively larger, and the walls thicken, due to the progressive increase in blood flow.^180^

The definition of maturation is extremely important, especially when attempting to answer the question at hand, about optimal maturation times. For instance, a fistula may be echographically “mature,” but only usable months later. That bias makes it difficult to standardize correct maturation times.

Traditionally, female patients have lower maturation rates than male ones. However, some studies indicate only the need for longer maturation time: the same fistula may take 22 additional days to mature in women compared to men with no specific cause.^181^ Cannulation time also differs significantly among countries. The DOPPS study showed that 74 percent of AVFs were punctured within 30 days in Japan, 50 percent in Europe, and only 2 percent in the U.S. Within 2 months, those same numbers increased to 98 (Japan), 79 (Europe) and 36 percent (U.S.).^91^ If successful cannulation is the main sign of adequate maturation, we can make mistakes about maturation times.

International guidelines on the creation of vascular access for hemodialysis recommend:^84^

- preoperative evaluation with physical examination and Doppler ultrasound to assess vessel caliber and patency;
- optimized surgical technique;
- postoperative assessment within 1-2 weeks to prevent complications such as infection and assess AVF patency (palpable thrill);
- new assessment within 6 weeks for possible approval for puncture;
- attempted punctures within 8 to 12 weeks;
- unsuccessful cannulation after 12 weeks—request Doppler ultrasound to assess possible causes of maturation failure and schedule intervention.

**Figure 4:**
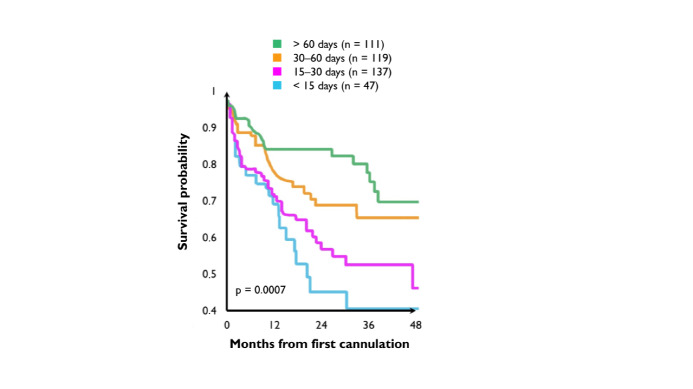
Kaplan-Meier analysis showing relationship between early cannulation and diminished access survival.^28^

Most vascular adaptations after completion of anastomosis, necessary to promote maturation, happen within 4 weeks^182^ Early attempts at cannulation, within approximately 14 days, double the li kelihood of access failure.^90,178^ In their 2004 study, Ravani et al.^28^ state that access cannulation less than 30 days after creation is associated with a statistically significant risk of decreased fistula survival (HR = 1.94, 95% CI 1.34-2.82) (Figure 4).^28^ Approximately 60 percent of native AVFs fail to mature.^95^ The advent of minimally invasive surgery has increased maturation rates: approximately one third of patients may undergo some type of intervention to facilitate maturation.^183^

Early fistula failure is frequently secondary to anatomical injuries that may exist at any point in the circuit. Arterial inflow injuries (4-8 percent), with occlusive atherosclerotic disease, may cause failure, especially in elderly and diabetic patients. Problems in the anastomosis (ranging from 4 to 64 percent) and the venous swing point (very frequent, 25 to 64 percent) are considered acquired conditions. Venous outflow injuries (high frequency, 33 to 59 percent) or central venous stenosis (3 to 9 percent) usually stem from prior punctures for temporary accesses. Non-maturing fistulas may have more than one associated injury.^184^ Assessment of fistulas presenting with maturation difficulties should be supplemented with Doppler ultrasound. This noninvasive examination is considerably superior to a simple physical examination in terms of selecting patients requiring additional procedures to promote cannulation.^185^

Patient-related factors may also hinder maturation, such as prior accesses, basal blood pressure levels, and, during dialysis, Black or Hispanic ethnicity.^186^ There are conflicting studies for diabetic patients, evidencing the complex relation between the disease and maturation rates. Elevated glycosylated hemoglobin levels are associated with AVF failure.^187^ Lower bioavailability of non-maturation and higher prevalence of severe atherosclerosis may be predictors of non-maturation.^188^ Obesity, with BMI above 29.5, is an independent risk factor for failure.^189^ Elderly patients, women, and patients with associated coronary artery disease also have higher rates of non-maturation.^190^

An extensive Cochrane library review found no benefit from any type of physical exercise for AVF maturation rates.^191^ However, some physical exercise is a relatively innocuous measure, and it helps keep patients engaged with the process of access creation. The use of antiplatelet medication may decrease thrombosis rates but does not increase maturation rates. Currently, the recommendation is that patients taking these drugs for other reasons should continue taking them.^192^

In short, despite the magnitude of the problem of vascular access for chronic kidney disease patients, there is a lack of effective clinical, demographic and biological markers to predict the exact maturation time of a native AVF.

#### **Question 9** - Are routine clinical examinations recommended for access surveillance?

Yes. At the moment, monitoring by routine clinical examination is strongly recommended. The use of other methods should be secondary^84,193^ (level of recommendation 1B).

##### Justification

The fundamental principal of routine surveillance of vascular accesses for hemodialysis is the identification and correction of potential stenoses with the goal of optimizing dialysis quality and minimizing the risk of access loss. Routine monitoring via physical examinations should preferably be performed by a knowledgeable professional in order to detect clinical signs of fistula flow dysfunction. The literature strongly recommends it, but with moderate quality of evidence.^194-199^ Clinical surveillance may be supplemented by regular laboratory tests at dialysis clinics, Kt/V analysis, cannulation difficulty, prolonged bleeding time, signs of recirculation, and flow measurement, among others. All of these options may help identify access dysfunction, but no scientific evidence supports the superiority of these methods over clinical examination.

Clinical examination performed by a knowledgeable professional, with experience in vascular access for hemodialysis, has high rates of sensitivity and specificity for the identification of stenoses in native fistulas and AVGs.^197-199^ A prospective study conducted by Asif et al.^197^ assessed the effectiveness of physical examinations in detecting stenoses compared to a fistulogram, considered the gold standard for stenosis detection. The sensitivity and specificity rates of physical examination were 92 and 86 percent, respectively, for outflow injuries, and 85 and 71 percent for occlusion of inflow segments.^197^ A prospective analysis by Campos et al.^200^ from 2008 assessing the effectiveness of physical examinations in detecting stenoses compared to Doppler ultrasound found rates of sensitivity, specificity, positive predictive value, and negative predictive value of 96, 76, 86, and 93 percent, respectively. The results from these studies showed that clinical examination, when performed by a knowledgeable professional with experience in vascular accesses, may be safely used to identify dysfunctions in vascular accesses for hemodialysis, supporting its use in routine practice.

There is not standard periodicity for clinical examinations.^201^ Some authors recommend vascular access surveillance based on monthly flow measurement for AVGs and every 3 months for AVFs.^196,198^ However, at the moment there is no evidence to recommend routine surveillance of vascular access based on flow measurement and pressure monitoring; the latter methods supplementing clinical examination and fail to improve access patency rates when performed exclusively.^198,202^

Routine Doppler ultrasound associated with clinical monitoring found no benefit over clinical examination alone in decreasing rates of vascular access loss.^194,203-209^ The results found by Tonelli et al.^210^ in their 2008 systematic review make it clear that the use of Doppler ultrasound in surveillance programs did not decrease rates of thrombosis and access loss for AVGs. For native AVFs, there may be some benefit in decreasing thrombosis rates; however, there was no decrease in the risk of vascular access loss for native AVFs.^210^ Based on these data, one cannot recommend the use of Doppler ultrasound in active surveillance of AVFs for hemodialysis, and imaging examinations should be considered in the presence of clinical signs of dysfunction.^194,199,205^

#### **Question 10** - Is there a standard treatment for vascular access-induced ischemia?

No. Treatment depends on a thorough clinical assessment and imaging examinations to determine the severity of the ischemia, the patient's clinical condition, fistula type, access functionality and quality, presence and location of potential arterial occlusions, patient's vascular anatomy, and fistula flow volume (level of evidence — expert opinion).

##### Justification

After the creation of a fistula, major local and systemic hemodynamic changes occur due to the connection between two distal vascular beds with different resistances (distal arterial bed and venous bed) to the same arterial inflow.^211,212^ As a result, most of the arterial flow is shunted to the venous bed,^213^ where resistance is lower, creating a pathological shunt that may lead to decreased flow or even reverse flow in arteries distal to the anastomosis.^211,212,214-217^ The ischemic signs and symptoms caused by these alterations is known as vascular access steal syndrome (VASS). The most important ones are limb pain at rest or during hemodialysis sessions, neurological abnormalities (paresis and paresthesia), and digital ulcerations and gangrene. ^213,215,217,218^ This is a dramatic situation, since it usually affects the upper limbs, which have significant functional versatility and are key for countless daily activities, and can even lead to amputation.^211^

The changes are most significant for fistulas created using the brachial artery at elbow level,^213-215,217-222^ and can lead to clinically significant VASS in 1 to 10 percent of fistulas.^211-215,217-220,222,223^

Diagnosis is based on clinical history and physical examination.^213,215,222^ Supplementary examinations can aid therapeutic planning more than diagnosis, considering the high incidence of anastomosis abnormalities,^215,217^ such as reverse flow distal to the anastomosis, found in 73.3 percent of radiocephalic AVFs and 90.9 percent of straight AVGs.^216^ The most frequently used tests are color Doppler ultrasound, arteriography, digital oximetry, digital photoplethysmography, digital blood pressure assessment, and invasive blood pressure measurements.

The best treatment strategy is always the identification of patients at high risk for VASS and the adequate choice of vascular access type.^212,222^ The primary risk factors identified in the literature are: age > 60, female gender, presence of peripheral or coronary artery disease diabetes mellitus, clopidogrel use, native brachial artery AVF, straight AVG on arm (using brachial artery).^212,215,220-222^

The first stage for therapeutic management of the disease is classifying its severity (Table 3).

**Table 3:** Clinical classification of severity of arteriovenous fistula steal syndrome.

**Stage**	**Signs and symptoms**	**Management**
I	Pallor / coldness / painless cyanosis	Clinical
IIa	Tolerable pain during exercise or hemodialysis	Clinical
IIb	Intolerable pain during exercise or hemodialysis	Surgical
III	Rest pain or motor deficit	Surgical
IVa	Limited tissue loss	Surgical
IVb	Extensive tissue loss	Surgical (amputation)

In stages I and IIa, the disease requires clinical treatment, including warming up the limb, exercising to improve collateral circulation, analgesia, and avoiding injuries to the limb.

At stage IVb, treatment is amputation of the nonviable limb.

In stages IIb, III and IVa, treatment can range from ligation of fistula or revascularization of the limb. Keep in mind that the priorities are saving the patient's life, followed by saving their limb, and finally saving the vascular access. Ligation of vascular access is the gold standard for resolving VASS, but it does not salvage the access,^212,220,221^ requiring the arduous process of obtaining a new permanent vascular access for hemodialysis to start over again. In patients with high surgical risk, low life expectancy or dysfunctional and low-quality fistulas, the best course of action is ligation of fistula and creation of new access (AVF, AVG, or catheter). The same strategy is valid for elderly patients and obtaining a new uncomplicated vascular access is a viable strategy, where ligation and creation of a new fistula does not compromise survival.

After verifying the indication for surgical revascularization of the limb, the next step is to perform imaging examinations to assess the anatomy and hemodynamics of the limb and the fistula, enabling proper surgical planning. In general, color Doppler ultrasound and angiography are indicated.

**Figure 5:**
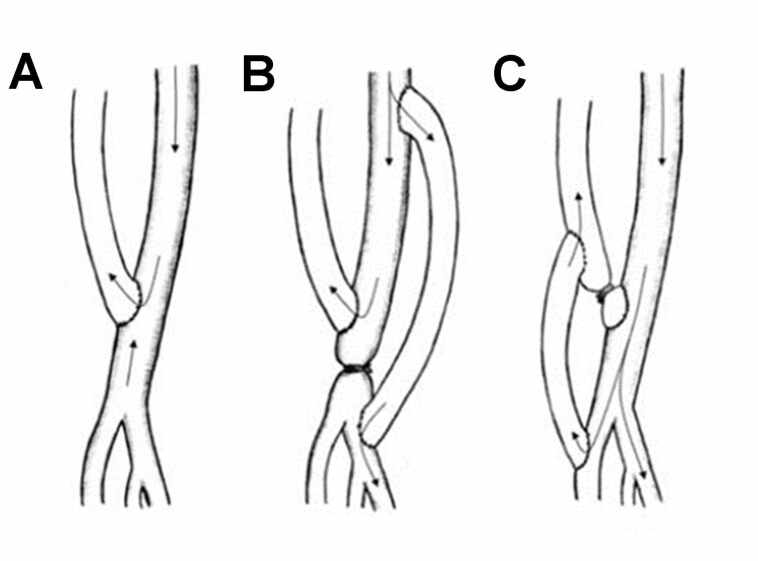
**A**) Schematic representation of brachiocephalic arteriovenous fistula. Adapted from Minion et al.^219^**B**) Schematic representation of distal revascularization interval ligation, consisting of ligation of the distal brachial artery to the anastomosis, with interruption of the reverse flow, and distal revascularization with bypass, placing the proximal anastomosis 5 cm above the arteriovenous anastomosis, thus avoiding a low blood pressure zone. Adapted from Minion et al.^219^**C**) Schematic representation of revision surgery using distal inflow consisting of proximal radial artery bypass (approximately 2 to 3 cm from its source) to the arteriovenous fistula vein, with ligation of the same juxta-anastomotic arteriovenous fistula. Adapted from Minion et al.^219^

**Figure 6:**
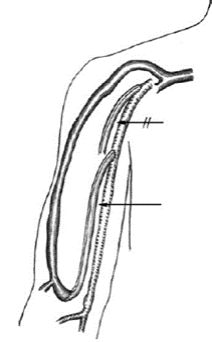
Schematic representation of proximalization of the arterial inflow surgery, consisting of converting the arterial supply of the arteriovenous fistula to the proximal artery with greater diameter and higher flow (axillary), leading to a lower arterial pressure drop distal to the anastomosis, associated with greater blood flow restriction from the use of a 4 or 5 mm expanded polytetrafluoroethylene graft. Adapted from Zanow et al.^220^

The next step in therapeutic planning for VASS is to rule hemodynamically significant stenoses proximal or distal to the anastomosis, since they are easily treated using an endovascular technique, with good results in terms of resolving symptoms.^211,213^ After ruling out injuries, treatment is based on surgical interventions. The primary options are: fistula ligation, banding, distal revascularization with interval ligation (DRIL; see Figures 5A and 5B),^217^ revision using distal inflow (RUDI; see Figures 5A and 5C),^219^ proximalization of arterial inflow (PAI; Figure 6),^220^ and arterial ligation distal to the anastomosis.

If access salvage is used, the choice of technique will depend on the fistula having high output or not, with thresholds set at flow > 800 mL/min for native AVFs or > 1000 mL/min for AVGs.^212,213,220^

For high output fistulas, the recommended techniques are those associated with flow restriction, such as banding and the RUDI technique.^213,219^ Banding was the first treatment described for vascular access salvage. It consists of decreasing the caliber of the vein or graft, with increased venous resistance and subsequent decrease in flow at the fistula. However, it has unpredictable results and high rates of access loss,^213,217,220,221^ extremely dependent on the degree of flow limitation.^222^ This form of treatment is currently reserved for fistulas with flow > 2000 mL/min.^212^ The RUDI technique is discussed less often in the literature, with good outcomes for VASS resolution, especially for access with flow > 1500 mL/min,^212^ but is also associated with some rate of access loss due to the use of a smaller-caliber donor artery (proximal radial artery).^218,219^

For fistulas with normal output, however, the recommended techniques are those that redirect flow, such as the PAI and DRIL procedures.^213^ The DRIL procedure has excellent results both for VASS resolution and for access salvage; however, it requires the ligation of a patent artery with good flow, making the distal limb perfusion dependent on the bypass.^219,220,222^ The PAI procedure has slightly inferior results to DRIL in resolving VASS, but high rates of access salvage, without the risk of compromising the axillary arterial axis in case of graft occlusion.^212^

Arterial ligation distal to the anastomosis yields good results both for resolution of VASS and for access salvage but is only indicated for radiocephalic AVFs.^212^

Ruling out differential diagnosis of ischemic monomelic neuropathy, characterized by immediate postoperative onset of high-intensity pain associated with paralysis, but with distal pulse present. Treatment for the condition consists of immediate surgical ligation of the arteriovenous access.

#### **Question 11** - Should one treat AVF or AVG-related asymptomatic stenoses?

No. Treatment of AVF-related stenosis should only be performed in the presence of clinical dysfunction or in case of documented inadequate dialysis/decrease in KtV. At the moment, there is no scientific evidence to support improved patency rates and decreased thrombosis rates after preemptive angioplasty of an asymptomatic fistula. The recommendation is valid for native AVFs, AVGs and the central venous system (level of recommendation 2B).

##### Justification

In most hemodialysis programs, different triage methods are routinely used for early identification of hemodynamically significant access stenosis.^7^ These triage methods consist of physical examination (monitoring) and surveillance-based strategies. Clinical monitoring includes assessing fremitus, murmurs, time to hemostasis after removal of needle, and limb assessment. Hemodialysis parameters, such as pump speed and transmembrane pressure, and dialysis adequacy rates (Kt/V or urea reduction ratio) are also part of the monitoring. Surveillance includes sequential measurements including intra-access flow tracking, recirculation analysis, dynamic or static venous pressure, blood pressure or duplex ultrasound imaging. According to recommendations from KDOQI 2006, preemptive angioplasties of vascular access-related stenoses should be performed in order to improve access patency rates and decrease thrombosis rates.^7^ However, more recent evidence has shown conflicting results when considering the outcome of improved access survival after preemptive angioplasty. Currently, few papers with high quality of evidence have assessed the results of treating fistulas with asymptomatic stenoses.^1^

An observational analysis by Chan et al.^224^ comparing preemptive angioplasty to clinical follow-up found no statistically significant results for the following outcomes: vascular access primary survival, secondary patency rates, and thrombosis rates. Rates of primary survival at 12 months were 53.7 per 100 access-years for the preemptive treatment group and 49.6 per 100 access-years for the control group (HR = 1.02, 95% CI 0.96-1.08). Subanalysis by fistula type (native or PTFE) also found no statistical difference between groups.^224^

In another prospective study from 2004, 64 patients were randomly assigned to the preemptive angioplasty or the clinical follow-up group to analyze AVG survival. Survival rates and time to access abandonment were similar across both groups. The authors reported lower graft thrombosis rates in the preemptive treatment group (72% versus 43%) (p = 0.04).^225^

On the other hand, a clinical trial by Tessitore et al.,^226^ from 2004, comparing primary and secondary patency rates for patients who underwent preemptive angioplasty compared to those whose stenosis was only treated in cases of native AVF dysfunction, found higher primary patency rates in the group treated preemptively (p = 0.021). There was statistical difference between groups in terms of secondary patency rates (p = 0.059).^226^

A systematic review by Ravani et al.,^227^ including 14 clinical trials (n = 1.390), found that preemptive treatment of AVF-related stenoses generally do not extend access survival. The analysis showed that preemptive interventions for native AVFs seem to improve primary patency rates (RR = 0.50, 95% CI 0.29-0.86) and lower the likelihood of thrombotic events (RR = 0.50, 95% CI 0.35-0.71). However, the meta-analysis found no increase in primary patency rates after prophylactic interventions in AVGs (RR = 0.87, 95% CI 0.69-1.11), as well no decrease in thrombotic events (RR = 0.95, 95% CI 0.80-1.12). There is moderate-quality evidence that preemptive interventions would probably not significantly decrease potentially preventable access failures, regardless of type.

In AVFs, technical surveillance and preemptive correction seem to have a more significant effect, but interpreting the relative and absolute effects obtained during this review requires caution. It is important to stress that the results were strongly influenced by three small studies performed at a single center.^226,228,229^ In addition, it is estimated that preemptive correction of 100 stenoses may, on average, prevent the loss of 5 fistulas as well thrombosis in 20 accesses—however, this virtual improvement is associated with an additional increase numbering 23.4 procedures, which may increase the risk of adverse events, health system costs, and mortality. In general, quality of evidence was low, most studies had high risk of bias, and the number of studies was small, with few participants and a high likelihood of false positives.

Regarding the central venous zone (subclavian vein, internal jugular vein, brachiocephalic vein, and superior vena cava), clinical manifestations ranged from asymptomatic conditions and few clinical repercussions to severe venous hypertension accompanied by skin lesions, ulcers, and inadequate dialysis. Approximately 15 to 20 percent of hemodialysis patients have some sign or symptom of central venous stenosis, in most cases associated with previous CVC use.^230-233^

The currently available evidence recommends not performing a central venous angioplasty in asymptomatic patients due to the risk of worsening the stenosis and rapid progression to symptomatic occlusion.^1,12,19,234-236^ A retrospective study of hemodialysis patients with asymptomatic central venous stenosis by Levit et al.^236^ showed that patients undergoing angioplasty had a higher likelihood of stenosis progression and worsening symptoms. In the clinical follow-up group, no patient progressed to symptomatic disease, while symptoms worsened for 8 percent of intervention group patients.^236^ In 2012, Renaud et al.^237^ retrospectively compared 103 patients with asymptomatic (n = 53) and symptomatic (n - 50) venous stenoses. Patients who did not present symptoms were followed up clinically, while the symptomatic patients group underwent balloon or stent angioplasty. Primary patency rates at 12 months (assessed as onset/return of symptoms) were significantly higher for the group that did not undergo interventions (77 versus 55 percent) (p = 0.002).^237^ Ehrie et al.^238^ and Chang et al.^239^ describe similar findings and suggest the clinical evolution of patients undergoing angioplasty seems to be more aggressive than that of patients submitted to clinical follow-up, but no intervention. Most likely, the endothelial injury caused by angioplasty leads to neointimal hyperplasia and more severe restenosis than the original injuries, which explains why symptoms worsen.

Therefore, considering the currently available evidence, we do not recommend preemptive intervention for AVGs with no sign of dysfunction and asymptomatic central venous stenoses with the goal of increasing vascular access survival time. In native AVFs, there seems to be some benefit to preemptive angioplasty. However, this potential improvement in access survival may be associated with an increased rate of complications, infection, and mortality. In addition, the data come from studies characterized by high risk of bias, low quality of evidence, and most patients from the same center, leading the group to recommend interventions only for fistulas presenting clinical signs of dysfunction.

#### **Question 12** - Is there a preferred mode of anesthesia for AVF creation?

Yes. Brachial plexus block has advantages compared to local anesthesia. There are randomized controlled trials and meta-analyses showing greater short-term patency when patients undergo brachial plexus block compared to local anesthesia.^240-244^ The benefit is greater for fistulas below the elbow^245,246^ (level of recommendation 1A).

##### Justification

Most arteriovenous fistulas and prosthetic grafts can be easily created using local anesthesia with lidocaine or bupivacaine without a vasoconstrictor. Ropivacaine has intrinsic vasoconstrictive properties and may cause vasoconstriction and hinder access to constricted vessels. Brachial plexus block causes vasodilation and may aid the management of blood vessels in the case of accesses for hemodialysis. There was an increase in venous and arterial diameters and in arterial flows in limbs submitted to brachial plexus block.^247-249^ However, this form of anesthesia requires a trained anesthesia team and is not universally available. Some studies have found higher rates of distal fistulas and lower use of prosthetics when regional block is used.^245,247,250^

In a randomized prospective study with 50 patients published by Yildirim et al.,^251^ 25 subjects underwent stellate ganglion block and 25 others made up the control group. The stellate ganglion block had better maturation rates. There was no statistical difference in terms of patency.^251^ In a meta-analysis on the subject by Cerneviciute et al.,^244^ 4 prospective and randomized controlled trials fit the inclusion criteria. In a 2011 study of 60 patients, 30 in each group, Meena et al.^241^ found higher patency and venous flow in patients submitted to brachial plexus block compared to local anesthesia. In a 2011 study of 60 patients, Sahin et al.^242^ found greater fistula flow and higher patency in the group submitted to brachial plexus block. In a 2011 study of 40 patients, Lo Monte et al.^240^ found greater vein diameter and lower vascular resistance in patients submitted to brachial plexus block. In a randomized trial from 2016 comparing local anesthesia to brachial plexus block, with 63 patients, for each arm, Aitken et al.^243^ found higher patency rate in 3 months (84 versus 62 percent) in the plexus block group. There was also a higher number of patients with radiocephalic fistulas in the plexus block group (77 versus 48 percent).^243^

However, important outcomes, such as long-term patency, and vascular effects from local anesthetics in the brachial plexus, were not widely studied. There is no robust evidence for fistulas above the elbow or for AVGs.

#### **Question 13** - In the presence of infection at AVF or AVG, is deactivation indicated?

No. If the patient is hemodynamically stable, with no infection on the anastomosis, and no life-threatening active bleed, and responds to conservative antibiotic therapy and adjunctive surgical procedures, attempting to salvage the infected vascular access is possible (level of evidence — expert opinion).

##### Justification

The infection rate for native AVFs is usually lower (from 2 to 4 percent) compared to AVGs.^102,190^ The incidence of infection in AVGs ranges from 1.6 to 35 percent, and is responsible for up to 35 percent of losses for this type of vascular access. PTFE, the most common material for prosthetic fistulas, is porous, facilitating the formation of biofilms, which in turn enables the proliferation of germs resistant to the body's innate defenses and to antibiotics.^252-259^ In terms of timing, infection peaks within 4 weeks of access creation, with an ascending curve over time afterwards.^260^

In upper limb fistulas, the microorganisms *Staphylococcus aureus* and *Streptococcus epidermidis* are the most frequent, while gram-negative bacteria the most common in the lower limbs. Polymicrobial flora and fungal infections are also possible.^253,261-266^

After fistula infection events, prevention and health education measures related to personal hygiene and antisepsis measures among health professionals are key, since both antisepsis and poor personal hygiene are risk factors for infection.^263^ Other known risk factors are: diabetes mellitus, hypoalbuminemia, advanced age, cannulation difficulty, hematomas after puncture, increased bleeding after needle removal, HIV infection, infections in other sites, increased number of surgical revisions, obesity, thrombotic and previously abandoned prostheses, and buttonhole cannulation.^267-270^

In their 2012 assessment of the use of covered stents to treat pseudoaneurysms in prosthetic arteriovenous hemodialysis access grafts, Kim et al.^271^ found a higher incidence of covered stent-related infections compared to bare metal and covered stents deployed within the graft for other reasons (42 versus 18 percent). The deployment site also seems to interfere in infection rates, with higher rates for stents deployed intragraft compared to other sites, such as at the venous anastomosis or outflow vein (26.6 versus 6.9 percent).^271^

Clinical diagnosis of infection should be based on findings from physical examination and patient history. The patient may present with pain, hyperemia, and local induration, similar to cellulitis, may progress to purulent secretion with or without abscess formation, pseudoaneurysms, and ulcerations, and may also suffer from hemorrhagic syndrome, with erosion and massive bleeding. In many episodes, a sentinel bleed precedes full rupture. In extreme cases, the condition may progress to sepsis and death.^254-256,265,272-274^

There are attempts to categorize the degree of infection and associated treatment. Standardization would make scientific research on the subject easier and enable us to compare data from various teams from around the world.^260^

**Figure 7:**
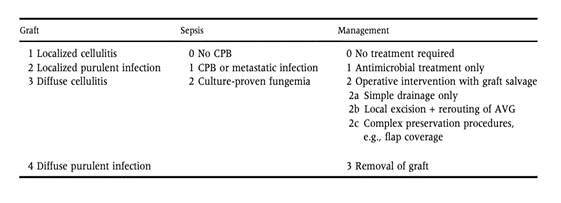
Categorization of arteriovenous fistula graft infection. Adapted from Kingsmore et al.^260^ CPB, positive culture; AVG, arteriovenous graft.

In that case, the patient's natural history would be summarized as the combination of letters and numbers from the classification system. For example, a patient with localized cellulitis, no culture-proven bacteremia, and receiving antimicrobial treatment only would be described as G1S0M1 (Figure 7).^260^

Imaging examinations, such as soft tissue ultrasound associated with Doppler ultrasound, could help diagnose venous or graft integrity, ruling out degeneration to pseudoaneurysms, or diagnose well-demarcated abscesses. The appearance of tissue infiltration around the fistula, often seen in B mode, is an alternative to assess the extent of infection, ruling out, for example, involvement of the anastomosis. Exams such as scintigraphy with labelled leukocytes or positron emission tomography may be used to diagnose infections in previously abandoned grafts, or in patients with mild and unspecific infection symptoms and no obvious local signs.^254-256,265,272-274^

Treatment should be initiated early, with the use of broad-spectrum antibiotics after collecting samples for culturing, usually for 6 weeks. Management should be tailored to the patient's clinical condition, considering severity of infection and the possibility of creating a new access after resolving the infectious process. Isolated use of antibiotics may be effective in treating limited and localized infections (not always possible in prosthetic graft infections), enabling salvage of the access. In these cases, the fistula should not be used before the infection is fully resolved; if the infection is restricted to small segments of the fistula, the access may be usable. Adjunctive surgical procedures including drainage and debridement are important to salvage the access, especially for native AVFs. For autologous accesses, when response to adjunctive treatment is poor, the infected vein segment may be resected, followed by reconstruction with interposition of the graft in an uninfected non-tunnelized pathway, simultaneously or at a later time. In grafts, if the patient is hemodynamically stable, with good systemic and local response to the initiation of antibiotic treatment, and if only one segment of the prosthesis has been affected by the infection, segmental explantation of the PTFE graft with in situ reconstruction with cryopreserved graft or extra-anatomical reconstruction with new prosthesis may be attempted, simultaneously or consecutively. In the same clinical conditions, but with greater involvement of the prosthetic graft, with salvage of the anastomosis, subtotal explantation of the prosthesis followed by reconstruction with interposition of the graft in an uninfected non-tunnelized pathway, simultaneously or at a later time, is required. On the other hand, if the patient presents with severe hemorrhagic syndrome, signs of severe infection or anastomosis infection, total explantation of the prosthesis followed by arterial vascular reconstruction is required. In some situations, for patients with indication for full graft explantation, but no involvement of the anastomosis, with that segment properly placed, a segment of the prosthesis may be salvaged as a “patch,” avoiding the need for complicated arterial reconstructions, risk of neurological injuries, and even arterial ligation.^255,256,261,275-280^ It is important to highlight that in cases of prosthesis infection involving the anastomosis and the brachial artery, dissection of that segment is associated with high risk of median nerve injuries. In these cases, to avoid extensive tissue loss with high risk of neurological injuries, brachial artery ligation is an option. Graft excision associated with ligation of the vessel at the level of the cubital fold is usually effective and well tolerated by patients, does not result in critical ischemia of the limb, and avoids complicated arterial reconstructions in infected regions.^278^

With the advent of covered stents, in hemodynamically unstable patients with severe hemorrhagic syndrome, deployment of this type of device as a bypass procedure is possible, with a definitive approach at a later date, once the patient's condition improves. Segmental and subtotal approaches for infected grafts need to be determined on a case-by-cases basis, since infection recurrence rates are 1.6 percent for full explantation, 19 percent for subtotal explantation, and 29 percent for partial explantation.^266,281,282^

#### **Question 14** - In the presence of an asymptomatic aneurysm, is surgical treatment indicated?

No. Asymptomatic aneurysms related to native or prosthetic fistulas can be treated conservatively with regular clinical surveillance, local treatment, guidance to avoid cannulation of aneurysmal segments, and patient education regarding possible complications (level of evidence — expert opinion).

##### Justification

**Figure 8:**
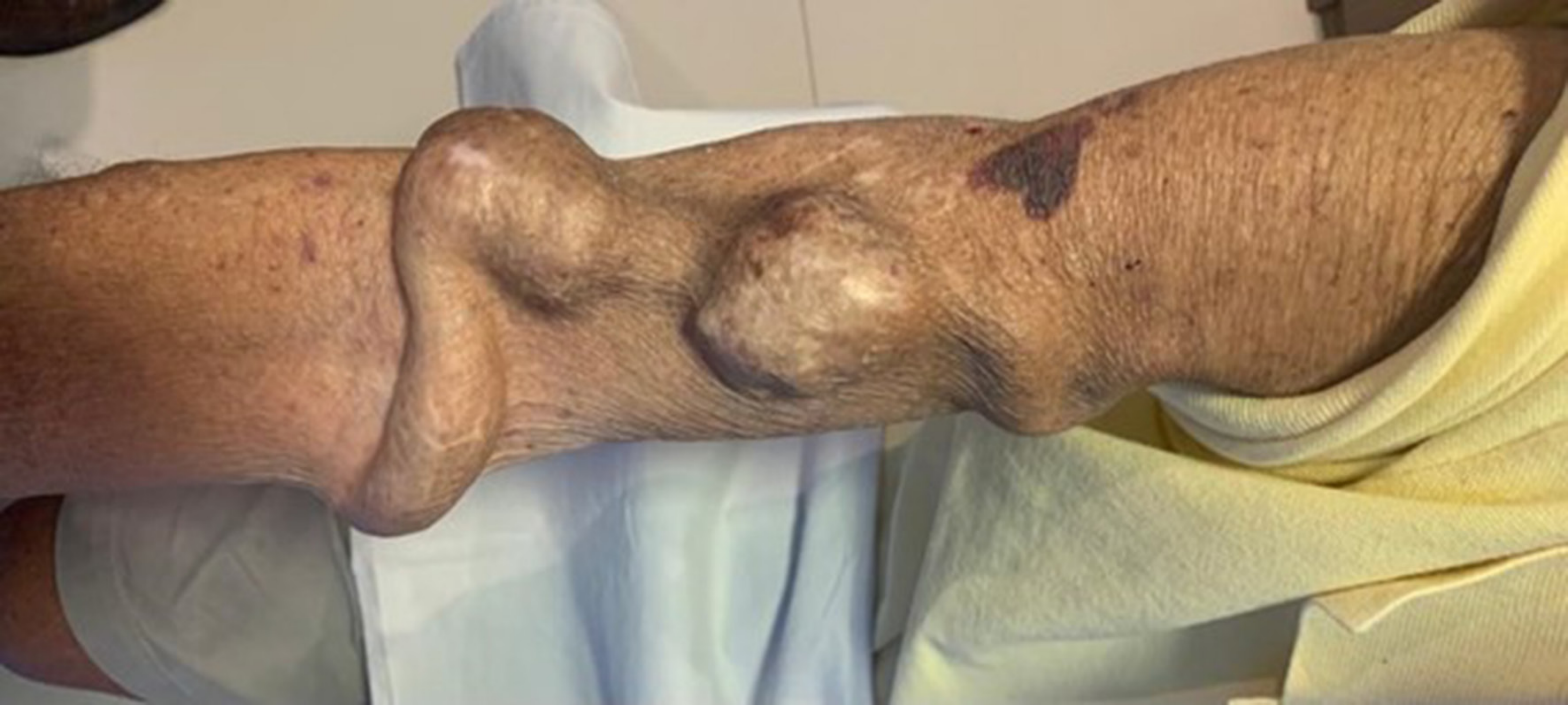
Asymptomatic true aneurysm.

Vasodilation after a fistula creation is a natural consequence of the hemodynamic and structural changes in arterial and venous circulation due to increased flow and vascular remodeling. The formation of true aneurysmal dilations and pseudoaneurysms are potentially severe complications that can occur in the presence of native and prosthetic fistulas. True aneurysms are those where, by definition, dilation involves all layers of the vessel, while in pseudoaneurysms there are discontinuities in the vessel wall and its coating structure is due to the formation of an extraluminal wall.^283^ True aneurysms are usually associated with hyperflow or the presence of stenoses, while pseudoaneurysms are usually located in puncture sites or anastomotic areas. The classical definition of an aneurysm is when a vessel is at least 50% greater than its normal size. However, there is no absolute value that defines when an AVF is aneurysmal. It should be stressed that if one were to follow the definition of an aneurysm to the letter, a mature AVF would have to be considered an aneurysmal vein. In an attempt to standardize the definition of an AVF with aneurysmal dilation, Balaz & Bjorck^284^ suggested that an AVF should be considered aneurysmal when its diameter is greater than 18 mm or approximately 3 time the diameter of the mature vein. There are other classifications based on absolute vessel diameter (> 20-30 mm), increased caliber compared to the adjacent segment (dilation to 2-3 times the proximal or distal diameter), the sum of longitudinal and transverse diameter, or vessel volume calculations.^285-289^ Finally, other authors recommend the term be understood more widely and define it as an “abnormal” dilation.^290^ Due to the wide variety of definitions, incidence rates may range from 5 to 60 percent of dialysis patients.^284,291^ The natural history of access-related aneurysms is little known, primarily because of the variety of existing classifications, high mortality rate, and high rates of vascular access loss associated with the routine of dialysis patients. There are several explanations for the formation of AVF aneurysms. Vasodilation after AVFs creation is a physiological response, necessary for proper AVF function. In some cases, the dilation may become pathological, leading to aneurysms with no plausible justification. Increased pressure within the circuit due to the presence of a stenosis, genetic predisposition, hyperflow and repeat cannulations are risk factors associated with the development of aneurysms.^287,288,290,292^ Most aneurysms are asymptomatic (Figure 8). However, the dilation may be accompanied by pain, skin lesions, ulcerations, aesthetic inconveniences, thrombus formation, cannulation difficulty, inadequate dialysis, congestive heart failure (in cases associated with hyperflow), and bleeding, which may jeopardize patients’ lives.

**Figure 9:**
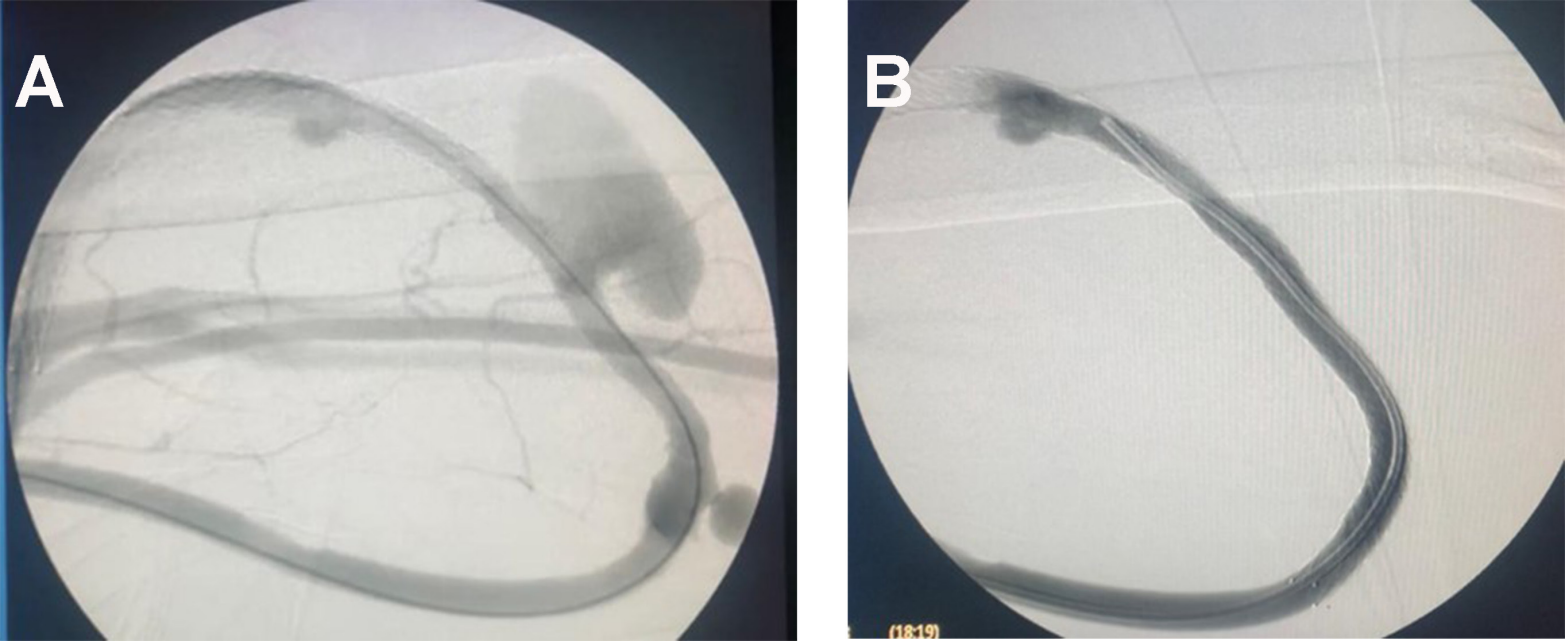
**A**) Ulcerated true aneurysm in brachiocephalic fistula associated with hyperflow and stenosis of the arch of the cephalic vein. **B**) Intraoperative appearance during aneurysmorrhaphy and reduction of hyperflow. **C**) Postoperative appearance.

**Figure 10:**
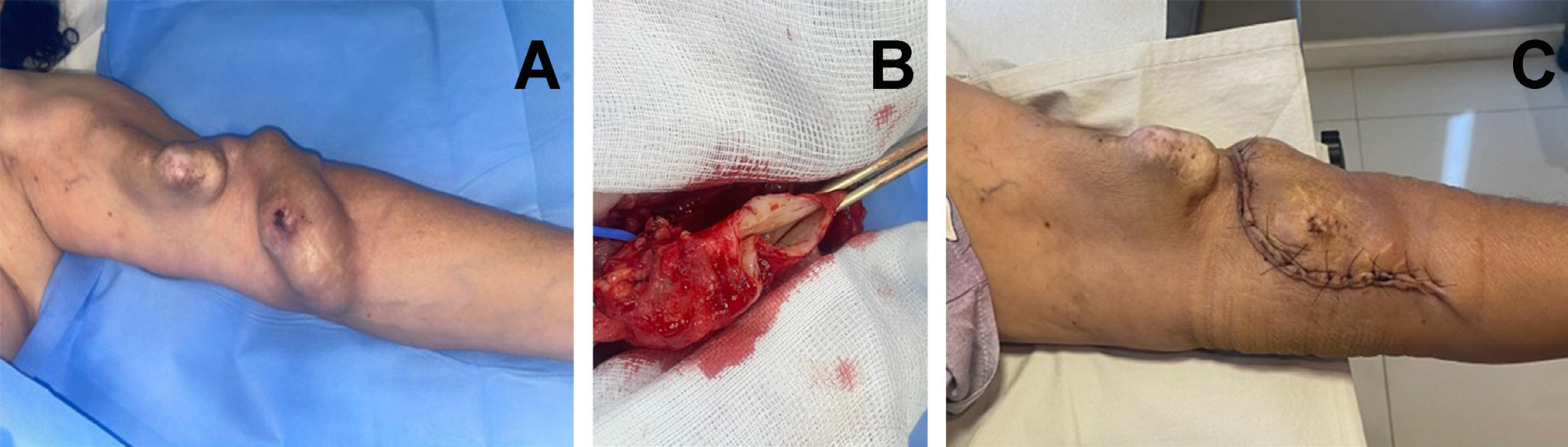
**A**) Voluminous pseudoaneurysm at expanded polytetrafluoroethylene graft puncture site. **B**) Appearance after correction with covered stent.

Diagnosis is primarily clinical, and Doppler ultrasound should be used to measure aneurysm diameter, flow analysis, and presence of associated thromboses or stenoses. Currently, the quality of existing evidence on treatment for AVF aneurysms is low, and standardizing treatment recommendations is not possible. However, the dilation is usually benign, remaining stable and asymptomatic over the long term.^290^ Therefore, treatment of aneurysms is not indicated for asymptomatic patients, while avoiding the cannulation of dilated segments is recommended. Patients should be taught about the importance of avoiding cannulation of aneurysmal segments, the importance of regular physical examinations for aneurysm surveillance, possible associated complications, risk of bleeding in case of ulcerations, and how to act in case of rupture.^1,19,39,293^ Surgical treatment is indicated in the presence of clinical symptoms, such as bleeding, ulcerations, skin lesions, pain, cannulation difficulty, unacceptable appearance, thrombosis or hyperflow (Figures 9A, 9B, and 9C). Aneurysm diameter alone is not indicative of surgical treatment. Pseudoaneurysms in cannulation areas are more concerning, since these cases are known to include vessel wall discontinuities that may be associated with higher risk of rupture. Since there is no clinical evidence to confirm the higher risk of pseudoaneurysm rupture in AVF cannulation areas, the recommendation stands to only treat symptomatic patients or cases of rapid growth. Lazarides et al.^294^ recommend a surgical approach to AVGs presenting pseudoaneurysms exceeding 2 times the graft diameter. In general, pseudoaneurysms located in anastomosis segments are treated with surgical correction, since most of the time they are associated with infection. There are several treatment options to correct AVF-related aneurysms and, as mentioned previously, no works comparing the outcomes for existing techniques. Treatment options include: aneurysm resection with graft interposition or end-to-end anastomosis, partial resection, aneurysmorrhaphy, covered stent implantation or ligation of vascular access^284,285,290,295-302^ (Figures 10A and 10B). It is important to highlight that whenever possible, we should consider salvaging the access, taking into account the possibility of other sites for AVF creation and patient life expectancy. During surgical treatment, possible stenoses or hyperflow associated with the development of the aneurysm should be corrected.
