# Supplementary material for: Guidelines on vascular access for hemodialysis from the Brazilian Society of Angiology and Vascular Surgery
Source: J Vasc Bras. 2023 Oct 20;22:e20230052. doi: 10.1590/1677-5449.202300522 (PMC10648056; doi:10.1590/1677-5449.202300522)
Supplement: Translation

# Translation: Diretrizes sobre acesso vascular para hemodiálise da Sociedade Brasileira de Angiologia e de Cirurgia Vascular

**DOI:** 10.1590/1677-5449.202300521

**Authors:** Leonardo de Oliveira Harduin, Julia Bandeira Guerra, Marcio Gomes Filippo, Leonardo Cortizo de Almeida, Guilherme de Castro-Santos, Fabio Augusto Cypreste Oliveira, Douglas Eduardo Tavares Cavalcanti, Ricardo Jayme Procopio, Eduardo Cavalcanti Lima, Matheus Eduardo Soares Pinhati, José Maciel Caldas dos Reis, Barbara D’Agnoluzzo Moreira, Adriano Martins Galhardo, Thiago Almeida Barroso, Edwaldo Edner Joviliano, Walter Junior Boim de Araujo, Julio Cesar Peclat de Oliveira

**Affiliations:** 1 Universidade Estadual do Estado do Rio de Janeiro – UERJ, Departamento de Cirurgia Vascular, Niterói, RJ, Brasil.; 2 Centro Clínico LIVCARE, Hospital Niterói D’or, Departamento de Imagem, Niterói, RJ, Brasil.; 3 Universidade Federal do Rio de Janeiro – UFRJ, Departamento de Cirurgia, Rio de Janeiro, RJ, Brasil.; 4 Hospital Ana Nery, Departamento de Cirurgia Vascular e Endovascular, Salvador, BA, Brasil.; 5 Universidade Federal de Minas Gerais – UFMG, Faculdade de Medicina, Departamento de Cirurgia, Belo Horizonte, MG, Brasil.; 6 Hospital Santa Helena, Programa de Residência Médica MEC em Cirurgia Vascular, Goiânia, GO, Brasil.; 7 Angiorad Recife, Departamento de Cirurgia Vascular, Recife, PE, Brasil.; 8 Universidade Federal de Minas Gerais – UFMG, Hospital das Clínicas, Cirurgia Endovascular, Belo Horizonte, MG, Brasil.; 9 Residência Médica em Cirurgia Vascular, Hospital Barão de Lucena, Recife, PE, Brasil.; 10 Residência Médica em Cirurgia Vascular, Instituto de Medicina Integral Prof. Fernando Figueira – IMIP, Recife, PE, Brasil.; 11 Universidade Federal de Minas Gerais – UFMG, Curso de Medicina, Belo Horizonte, MG, Brasil.; 12 Centro Universitário Metropolitano da Amazônia – Unifamaz, Habilidades Cirúrgicas, Belém, PA, Brasil.; 13 Universidade Federal do Paraná – UFPR, Hospital de Clínicas, Serviço de Cirurgia Vascular, Curitiba, PR, Brasil.; 14 Grupo DASA, Hospital Santa Marta, Brasília, DF, Brasil.; 14 Grupo DASA, Hospital Brasília Águas Claras, Brasília, DF, Brasil.; 16 Hospital Sírio Libanês, Brasília, DF, Brasil.; 17 Universidade de São Paulo – USP, Faculdade de Medicina de Ribeirão Preto – FMRP, Departamento de Cirurgia e Anatomia, Ribeirão Preto, SP, Brasil.; 18 Universidade Federal do Paraná – UFPR, Hospital de Clínicas, Residência Médica de Angiorradiologia e Cirurgia Endovascular, Curitiba, PR, Brasil.; 19 Sociedade Brasileira de Angiologia e de Cirurgia Vascular – SBACV, São Paulo, SP, Brasil.

**Keywords:** diálise renal, acesso vascular, diretriz

## Abstract

A doença renal crônica é um problema de saúde pública global e em seu estágio terminal está associada à necessidade de terapia dialítica. A grande maioria dos pacientes que necessitam realizar a terapia renal substitutiva, a fazem através da hemodiálise. Portanto, o acesso vascular é de extrema importância para a população dialítica, implicando diretamente na qualidade de vida e na morbimortalidade deste grupo de pacientes. Sendo a confecção, gerenciamento e resgate dos acessos vasculares uma das áreas de atuação do cirurgião vascular, é de grande importância a elaboração de uma diretriz que oriente o especialista no manejo mais adequado do acesso vascular para hemodiálise. Assim, o objetivo desta diretriz é apresentar um conjunto de recomendações para guiar as decisões na referenciação, avaliação, escolha, vigilância e gestão das complicações do acesso vascular para hemodiálise.

## INTRODUÇÃO

A doença renal crônica é um problema de saúde pública global, e é classificada em 5 estágios. A insuficiência renal, no entanto, é restrita aos estágios 3-5, com uma taxa de filtração glomerular abaixo de 60 mL/min/1,73 m^2^ por 3 meses ou mais, independentemente da causa^1^. A doença renal crônica estágio 5 é caracterizada pela taxa de filtração glomerular abaixo de 15 mL/min/1,73 m^2^ e inclui duas fases: a primeira é tratada conservadoramente, sem diálise; na segunda fase, a iniciação da terapia renal substitutiva na forma de diálise ou transplante renal é requerida para sustentar a vida. Atualmente, existem cerca de 140 mil pacientes em diálise no Brasil, e aproximadamente 90% desses pacientes realizam a terapia renal substitutiva através da hemodiálise^2^.

A hemodiálise somente pode ser realizada com um acesso vascular que apresente bom funcionamento. O acesso vascular ideal deve permitir a canulação com o uso de duas agulhas que sustentem um fluxo sanguíneo mínimo de 300 mL/min por meio de uma máquina que funciona como um rim artificial, deve ser resistente à infecção e trombose e apresentar o mínimo de efeitos adversos^1^. A hemodiálise pode ser realizada através de um cateter de curta permanência, de um cateter de longa permanência, de uma fístula arteriovenosa (FAV) com veia autóloga e de uma FAV com enxerto protético. A FAV é considerada o acesso vascular de escolha, e diversos estudos demonstraram que apresenta menores taxas de complicações pós-operatórias, custos de manutenção mais baixos e baixas taxas de revisões cirúrgicas ou endovasculares para a manutenção da perviedade, quando comparada a outras modalidades de acesso^3-6^. Além disso, o uso dos cateteres de curta e longa permanência resulta em altas taxas de morbidade e mortalidade quando comparadas com as FAVs nativas e com prótese. O risco de hospitalização, morte e, particularmente, de infecção relacionado ao acesso vascular para hemodiálise é muito superior nos pacientes que estão realizando a hemodiálise através de um cateter de curta ou longa permanência^7^. A maioria dos pacientes com doença renal crônica estágio 5 no Brasil que necessita de terapia dialítica se beneficiaria imensamente de uma FAV que apresente bom funcionamento para sustentar a vida.

Sendo a confecção, gerenciamento e resgate dos acessos vasculares uma das áreas de atuação do cirurgião vascular, é de grande importância a elaboração de uma diretriz que oriente o especialista no manejo mais adequado do acesso vascular para hemodiálise. Assim, o objetivo desta diretriz é apresentar um conjunto de recomendações para guiar as decisões na referenciação, avaliação, escolha, vigilância e gestão das complicações do acesso vascular para hemodiálise.

## MÉTODOS

O grupo de trabalho escolhido para a realização desta diretriz é composto por 14 cirurgiões vasculares com vasta experiência em acesso vascular para hemodiálise e grande atuação nas suas respectivas regionais. Inicialmente, cada membro da equipe formulou 10 perguntas relevantes para a prática clínica habitual, relacionadas ao acesso vascular. Após a eliminação das perguntas repetidas, foram escolhidas pelos membros as 14 perguntas mais relevantes. Cada pergunta foi respondida por um membro da equipe, considerando a melhor evidência científica de acordo com artigos publicados em língua inglesa e portuguesa.

O grupo utilizou como referência para as suas pesquisas bases de dados bibliográficas incluindo MEDLINE, SciELO Brasil, PubMed, Embase, LILACS e biblioteca Cochrane. Foram incluídos artigos publicados entre janeiro de 1995 a maio de 2022. Após a formulação das respostas, essas foram revisadas e discutidas pelo grupo de trabalho através de reuniões virtuais que culminaram com a elaboração final de cada recomendação. Os níveis de evidência de cada resposta foram classificados de acordo com o *Grading of Recommendations Assessment, Development and Evaluation* (GRADE)^8-10^ (Tabelas 1 e 2). Quando não houve evidência suficiente para a realização da graduação das respostas, de acordo com a escala GRADE, prevaleceu a opinião do grupo, que foi designada como “opinião de *expert*”. As perguntas selecionadas pelo grupo de trabalho foram:

**Tabela 1:** Escala GRADE – Qualidade da evidência^8-10^.

**A - Alta**	Há forte confiança de que o efeito verdadeiro se aproxima do efeito estimado.	**B - Moderada**	Há moderada confiança na estimativa do efeito. O verdadeiro efeito está próximo daquele estimado, mas existe possibilidade de ser substancialmente diferente
**C - Baixa**	A confiança na estimativa do efeito é limitada. O verdadeiro efeito pode ser substancialmente diferente daquele estimado.	**D - Muito Baixa**	Há pouca confiança na estimativa de efeito. O verdadeiro efeito provavelmente é substancialmente diferente do estimado.

**Tabela 2:** Escala GRADE – Nível de recomendação^8-10^.

1. Recomendação forte	Os efeitos benéficos do tratamento claramente superam os efeitos maléficos.
2. Recomendação fraca	Há menor diferença entre efeitos desejáveis e efeitos adversos.

- É mandatório o mapeamento pré-operatório para a criação de FAVs?
- Existe sítio ideal de punção para acesso de longa permanência para hemodiálise?
- É mandatório o uso de imagem no implante de cateter de longa permanência para hemodiálise?
- A FAV nativa é a primeira opção para acesso vascular para hemodiálise?
- É possível recuperar um cateter de longa permanência com disfunção?
- É mandatória a retirada do cateter de longa permanência na presença de infecção?
- Existe um diâmetro mínimo ideal de vasos para a confecção do acesso vascular para hemodiálise?
- Existe um período ideal para a maturação das FAVs?
- O exame clínico de rotina é recomendado para a vigilância do acesso?
- Existe um tratamento padronizado para a isquemia induzida pelo acesso vascular?
- Devemos tratar as estenoses assintomáticas relacionadas às FAVs?
- Existe uma modalidade preferencial de anestesia na criação das FAVs?
- Na presença de infecção da FAV, está indicada a desativação do acesso?
- Na presença de aneurisma assintomático, está indicado o tratamento cirúrgico?

### Perguntas

#### Pergunta 1 – É mandatório o mapeamento pré-operatório para criação de FAVs para hemodiálise?

Não. Apesar de todos os esforços em identificar métodos que reduzam as taxas de falência primária das FAVs autógenas, não há consenso entre os estudos existentes quanto à eficácia do mapeamento vascular pré-operatório (nível de evidência 2C).

##### Justificativa

As FAVs nativas funcionais são consideradas o acesso vascular de escolha para hemodiálise^11,12^. Entretanto, alcançar uma FAV funcional em pacientes dialíticos é um desafio, com taxas de falência primária variando entre 23-46%^13^. O exame físico tem sido tradicionalmente utilizado para identificação dos vasos apropriados para confecção da FAV^14^. Alguns autores recomendam a realização do mapeamento vascular pré-operatório com eco-Doppler com o objetivo de reduzir as taxas de falência primária^15,16^.

Uma revisão sistemática publicada por Georgiadis et al.^17^ encontrou uma diminuição no risco de falência primária no grupo de pacientes submetidos ao mapeamento pré-operatório (OR = 0.32, 95% CI 0.17-0.6; p < 0,01). Os autores concluíram que o mapeamento pré-operatório deve ser realizado em todos os pacientes antes da confecção de uma FAV. Entretanto, em uma revisão sistemática Cochrane envolvendo 450 pacientes, Kosa et al.^18^ concluíram que o mapeamento vascular pré-operatório não alterou as taxas de maturação das FAVs. Não houve diferença significativa: no número de FAVs criadas com sucesso (RR = 1.06, 95% CI 0.95-1.28); no número de FAVs maturadas em 6 meses (RR = 1.11, 95% CI 0.98-1.25); no número de FAVs que foram utilizadas para hemodiálise (RR = 1.12, 95% CI 0.99-1.28); entre o uso de mapeamento pré-operatório comparado com exame físico isolado^18^.

Considerando os resultados conflitantes, a falta de evidência científica de qualidade, a possibilidade de atraso na confecção do acesso e o aumento do custo com a realização mandatória de exame pré-operatório, principalmente na esfera pública dos serviços de saúde, definimos que, nos pacientes com exame físico confiável e com baixo risco de falência de FAV, o mapeamento vascular pré-operatório não é mandatório. Importante considerar que, nos pacientes com alto risco de falência de FAV (idosos, mulheres, doença arterial obstrutiva periférica, doença arterial coronariana, obesos, crianças, pacientes com histórico de múltiplos acessos) ou com exame físico inconclusivo, há indicação para a realização do mapeamento pré-operatório, de forma a melhorar o resultado na confecção de uma FAV^19^.

Na avaliação do território venoso central, a ultrassonografia (USG) com Doppler pode não ser um exame confiável. Nos pacientes com histórico de múltiplos cateteres venosos e com alta probabilidade de doença venosa obstrutiva central, a flebografia diagnóstica deve ser considerada^12,20,21^.

#### Pergunta 2 – Existe sítio ideal de punção para acesso de longa permanência para hemodiálise?

**Sim**. A escolha do sítio para o implante do cateter venoso central (CVC) de longa permanência deve ser individualizada, considerando otimizar as opções de acesso, e realizada após análise cuidadosa de diversos fatores, como necessidade de diálise em caráter emergencial, expectativa de vida, perspectiva de confecção de FAV nativa ou com prótese, probabilidade de maturação da fístula e previsão de retirada do cateter, possibilidade de transplante renal e escolhas do paciente. Devemos considerar os seguintes pontos durante a escolha do sítio de punção de um cateter de longa permanência:

- extremidade superior antes da extremidade inferior somente se as opções forem equivalentes;
- se houver previsão de um acesso por uma FAV em um futuro próximo, considerar preferencialmente um cateter tunelizado na extremidade oposta à FAV pretendida;
- se há previsão de transplante em um futuro próximo, considerar preferencialmente um cateter tunelizado na veia jugular interna (preservar as veias ilíacas);
- como primeira opção para o implante de um CVC, recomenda-se a punção da veia jugular interna direita;
- evitar o acesso através das veias subclávias nos pacientes que realizarão uma FAV, devido ao risco aumentado de doença oclusiva venosa central;
- alguns especialistas sustentam que, em situações de início urgente de hemodiálise, em determinadas circunstâncias (retirada precoce do cateter) e quando o transplante não for uma opção, o uso de um CVC na veia femoral é aceitável (a não ser que haja contraindicação) até que uma FAV seja criada ou cateter de diálise peritoneal utilizado. O uso da veia femoral preserva os vasos da extremidade superior para futura criação de uma fístula.

As contraindicações para cateter femoral incluem doença femoral ou ilíaca, reconstrução/cirurgia prévia, razões higiênicas (como diarreia crônica), obesidade mórbida (índice de massa corporal [IMC] maior que 35) ou outras dificuldades de acesso venoso.

Quando há razões para o uso de cateter e a duração estimada é prolongada (maior que 3 meses), sem antecipação do uso de uma FAV, o CVC deve ser posicionado nos seguintes sítios na ordem de preferência:

- veia jugular interna;
- veia jugular externa;
- veia femoral;
- veia subclávia;
- translombar (inserção na veia cava inferior com ponta no átrio direito).

Nota: na ausência de contraindicações, doença prévia (por exemplo, estenose central, marca-passo), a inserção do CVC do lado direito é preferencial em relação ao lado esquerdo devido a uma anatomia mais retilínea. Se um lado tem doença que limita a criação de uma FAV, mas permite a passagem de cateter, esse lado deve ser usado, preservando o outro para criação de um acesso definitivo (nível de evidência – opinião de *expert*).

##### Justificativa

Os CVCs de longa permanência historicamente vêm sendo implantados preferencialmente na seguinte ordem: veias jugulares internas; veias femorais; e veias subclávias. Em situações de exceção ou impossibilidade de punção em sítios tradicionais, podem ser utilizadas a veia jugular externa, veia cava inferior, veias supra-hepáticas, veias renais, veias gonadais, veias poplíteas, veias safenas e veias colaterais cervicais, inguinais ou pélvicas^21-25^. A veia jugular interna direita é considerada na maioria das vezes como o acesso de escolha, devido às menores taxas de complicações quando comparadas com os outros sítios de punção e com a veia jugular interna esquerda. Em uma análise retrospectiva publicada por Engstrom et al.^26^, em 2013 (409 participantes e 532 cateteres), os cateteres implantados na veia jugular esquerda apresentaram maior risco de remoção relacionada à infecção quando comparados com os cateteres inseridos do lado direito (0.33 *versus* 0.24 por 100 cateter-dias; p = 0.012). Os cateteres implantados no lado esquerdo também apresentaram maior chance de troca devido à disfunção (0.13 *versus* 0.08 por 100 cateter-dias; p = 0.08); entretanto, essa diferença não foi significativamente estatística. Esses resultados foram modificados baseados na posição da ponta do CVC. Para CVC posicionados na veia cava superior ou junção cavo-atrial, a disfunção e infecção do CVC foram maiores para os implantes do lado esquerdo. Porém, para CVC com pontas posicionadas no meio do átrio direito, a disfunção e infecção do CVC foram similares entre o lado esquerdo e direito.

Estudos têm demonstrado resultados inferiores relacionados à sobrevida e taxa de maturação das fístulas arteriovenosas nos pacientes que estão realizando a diálise através de um cateter, principalmente quando este encontra-se inserido em posição ipsilateral à fístula^19,27-29^ (Figuras 1 e 2). Alguns autores sugerem que a inserção de um cateter de longa permanência na veia subclávia de um paciente com indicação de hemodiálise somente deve ser realizada em casos em que não existam outros sítios de punção, devido ao alto risco de estenose venosa central^23,30,31^. A incidência de estenose da veia subclávia relacionada à colocação de um cateter varia na literatura entre 42 e 50%. Em contraste, a estenose da veia inominada associada ao uso de cateter na veia jugular interna varia entre 0-10%^23,32^. Sendo assim, quando utilizarmos um braço para a confecção de uma fístula, devemos evitar a inserção de um cateter na veia jugular e principalmente na veia subclávia ipsilateral.

**Figura 1:** Análise de Kaplan-Meier demonstrando a relação entre a presença de um cateter venoso central e a diminuição da sobrevida do acesso^28^.

**Figura 2:** Análise de sobrevidas das fístulas arteriovenosas com a presença de cateter venoso central ipsilateral e contralateral^29^.

A veia jugular interna é a veia mais frequentemente escolhida para colocação de um CVC, devido a seu fácil acesso e menor taxas de complicações^33-35^. Como segunda opção e sujeito à controvérsia devido às características anatômicas e funcionais do paciente, muitos autores consideram a veia femoral comum como uma alternativa, quando não é possível a colocação em veias jugulares. Os cateteres inseridos na veia femoral apresentam uma taxa de perviedade primária menor (44% ao mês) e uma maior taxa de infecção (63/1.000 dias de cateter)^30,36^ quando comparados com os acessos inseridos nas veias jugulares. Em algumas situações específicas, podemos considerar o implante do cateter na veia femoral como primeira opção. Nos pacientes com necessidade de iniciar a terapia dialítica em situações de emergência, que não são candidatos ao transplante renal e que têm a expectativa de confecção precoce do acesso com utilização em 30-60 dias, pode haver algum benefício em realizar o implante do acesso na veia femoral ao invés da veia jugular como realizado rotineiramente. Algumas vantagens poderiam justificar essa estratégia como uma opção razoável. Primeiro, o implante de um acesso através da veia femoral pouparia o eixo superior de danos endoteliais e risco elevado de estenose venosa central. Além disso, a presença de um cateter na veia femoral pode fazer com que os pacientes se conscientizem com mais frequência da necessidade da realização de uma fístula. É importante ressaltar que algumas revisões sistemáticas falharam ao tentar demonstrar o menor risco de complicações, como tromboses e infecções relacionados aos cateteres de veia jugular, quando comparados com os acessos implantados na veia femoral^32,37,38^. Apesar disso, o acesso através das veias jugulares deve ser considerado como primeira escolha, devendo a inserção pela veia femoral ser utilizada com precaução e em situações específicas^1,19,39^.

#### Pergunta 3 – É mandatório o uso de imagem no implante de cateter de longa permanência para hemodiálise?

Sim. O implante de cateter de longa permanência para hemodiálise em veias centrais deve ser realizado em centros de referências, por profissionais médicos e guiado por ultrassonografia e radioscopia (nível de evidência 1B).

A punção das veias centrais para o implante de cateter de longa permanência deve ser realizada sob orientação ultrassonográfica, minimizando os riscos de complicações relacionadas ao cateterismo (nível de evidência 1A).

A radioscopia é o método de eleição para o implante e posicionamento adequado da ponta do cateter de longa permanência para hemodiálise e deve ser realizado sempre que possível (nível de evidência 1B).

Na ausência de radioscopia disponível para o implante de cateter de longa permanência para hemodiálise, outro método diagnóstico deve ser realizado após o seu implante para averiguação do correto posicionamento da ponta do mesmo, sendo a radiografia convencional o método mais comumente realizado (nível de evidência – opinião de *expert*).

##### Justificativa

A disfunção imediata dos cateteres venosos centrais para hemodiálise (CVCHs) pode ser definida pela presença de fluxo médio menor que 300 mL/minuto ou a incapacidade de realizar uma sessão de hemodiálise por fluxo inadequado^39^. Dessa forma, o sucesso no implante dos CVCHs e o seu correto posicionamento são fundamentais para uma terapia renal substitutiva adequada, tendo o uso de métodos de imagem papel fundamental para atingir a excelência em ambos os pontos.

A inserção dos CVCHs pode ser realizada mediante a utilização dos pontos anatômicos de referência ou com auxílio de ecografia. A sua inserção guiada por ultrassom reduz as taxas complicações precoces e tardias, além de otimizar a experiência e a satisfação dos pacientes dialíticos^40,41^. Em uma metanálise Cochrane publicada por Rabindranath et al.^42^, em 2011, com a análise de 7 ensaios clínicos randomizados e inclusão de 767 pacientes submetidos à inserção de cateteres em veias jugular e femoral, os autores concluíram que a utilização do ultrassom reduziu significativamente o índice de falhas no implante do cateter (RR = 0.40, 95% CI 0.3-0.52), o risco de punção arterial (RR = 0.13, 95% CI 0.04-0.37) e a formação de hematomas (RR = 0.22, 95% CI 0.06-0.81), quando comparado com a inserção auxiliada somente pelos pontos anatômicos. O uso da ecografia também reduziu significativamente o tempo para a inserção do cateter e o número de tentativas até a inserção bem-sucedida. Não houve diferença entre a inserção com o auxílio do ultrassom ou com a utilização das referências anatômicas em relação ao risco de pneumotórax ou hemotórax (RR = 0.23, 95% CI 0.04-1.38).

Outros autores relataram que o uso da ultrassonografia para a inserção dos CVCHs foi capaz de reduzir significativamente complicações precoces, como hematoma, pneumotórax e punção arterial inadvertida, além de reduzir as taxas de complicações tardias, como mau posicionamento do cateter, perfuração vascular e trombose. Outro fator importante na indicação do uso da ultrassonografia em todos os acessos venosos profundos é o aumento significativo na taxa de sucesso na inserção do CVCH com o seu auxílio^19,30,40,43-49^. Dessa forma, a inserção dos CVCHs baseada somente em pontos anatômicos fica restrita à impossibilidade ou ausência de disponibilidade de ultrassonografia. O correto posicionamento da ponta dos cateteres de longa permanência para hemodiálise (CLPH) através da radioscopia representa atualmente o método mais preciso, que vem sendo cada vez mais recomendado^40,42,49-53^.

Tanto na inserção pelas veias jugulares como pelas veias femorais, a radioscopia deve ser realizada sempre que disponível, de forma a posicionar a ponta do CLPH no meio do átrio direito no caso do acesso pela veia jugular interna, e na veia cava inferior em posição central (não distal) em caso de acesso pela veia femoral, evitando assim complicações e fluxo inadequado^19,40,42,49,52^. Um estudo observacional publicado por Yevzlin et al.^54^, em 2007, comparando os resultados da inserção de cateteres de longa permanência com o auxílio de radioscopia *versus* a técnica convencional sem a radioscopia, evidenciou que os cateteres inseridos pela técnica tradicional apresentam diminuição das taxas de sucesso imediato (OR = 0.12, CI = 0.02-0.71). Na ausência de disponibilidade de radioscopia, outro método de imagem deve ser realizado após o implante dos CLPHs para avaliação do posicionamento adequado da ponta do mesmo, tanto no tórax como no abdome, sendo a telerradiografia a mais comumente recomendada^40,42,49,51^.

Em resumo, a utilização de métodos de imagem para a inserção de CLPH torna o procedimento mais seguro e eficaz, e deve ser a opção em serviços de referência com disponibilidade dos mesmos e por profissionais treinados^49,55,56^. A associação de ultrassonografia para canulação venosa e a radioscopia para o correto posicionamento do CLPH reduzem de forma significativa as taxas de complicações maiores e mau funcionamento precoce^52^.

#### Pergunta 4 – A FAV nativa é a primeira opção para acesso vascular para hemodiálise?

Nem sempre. A escolha do acesso deve ser individualizada para cada paciente, e a decisão deve ser baseada em diversos fatores, como expectativa de vida, probabilidade de maturação da FAV, escolhas do paciente, tempo para o início da terapia dialítica, comorbidades, escala de fragilidade, tempo de retirada do cateter, riscos de complicações, plano de gerenciamento do acesso e análise de equipe multidisciplinar (nível de evidência 2C).

##### Justificativa

Desde o desenvolvimento do conceito de FAV como acesso vascular definitivo por Brescia, Cimino, Appel e Hurwich na década de 60, houve um aumento exponencial da quantidade de pacientes dialíticos com o avanço tecnológico progressivo da terapia renal substitutiva, gerando uma desproporção entre demanda e possibilidade de oferta de acesso vascular. Tal situação gerou condições ideais para o avanço de uso do enxerto protético, destacando-se o desenvolvimento do politetrafluoroetileno (PTFE) expandido pela W.L. Gore & Associates na década de 70, e utilização de cateteres semitunelizáveis, que também tiveram um importante crescimento na década de 80. O resultado final foi um aumento expressivo de fístulas protéticas e cateteres semitunelizáveis e redução da quantidade de FAVs nativas, promovendo um cenário de alto custo e necessidade de hospitalizações para gerenciamento desses pacientes. Poderíamos considerar esse primeiro período do acesso vascular como “graft and catheter first”^57-59^.

O grande problema em relação aos cateteres de diálise é a infecção, não se tratando de uma questão de possibilidade (“se vai acontecer”), mas de temporalidade (“quando vai acontecer”). A taxa de infecção varia de 2.5 a 5.5 casos/1.000 paciente-dias, ou 0.9 a 2.0 episódios/paciente-ano, destacando-se um risco 40% maior dos cateteres temporários quando comparados aos de longa permanência. Conforme publicado por Allon et al.^60^, o risco de bacteremia é proporcional ao tempo de dependência do dispositivo, sendo de 35% em 3 meses, 54% em 6 meses e 79% em 12 meses, em uma amostra de 472 pacientes em hemodiálise com cateter. Um agravante é o comprometimento imunológico associado à doença renal crônica que predispõe a ocorrência de sepse, aumentando consideravelmente o risco de morte (5 a 9 vezes). Estima-se que em 10% dos casos de bacteremia relacionada a cateter (BRC), há ocorrência de complicações graves, tais como endocardite, meningite, artrite séptica, espondilodiscite, choque séptico e, eventualmente, morte. O risco relativo de morte associado ao uso do cateter como acesso vascular permanente quando comparado com as FAVs nativas aumenta 1,4 a 3,4 vezes. Além disso, há associação de aumento da taxa de hospitalização não apenas por sepse, mas aumento de hospitalização em geral, resultando em custo anual maior nesse grupo de pacientes (dados da Medicare apontam para um aumento de custo anual médio de U$ 20.000 quando comparados a pacientes com fístula, sendo o maior componente desse custo as hospitalizações). Outra complicação não menos importante, com grande impacto na qualidade de vida e interferência na obtenção de um acesso vascular permanente adequado no membro, é a estenose central. Sabe-se que há relação direta entre o tipo de dispositivo, veia central e tempo de permanência do cateter, podendo chegar a 50% o risco de lesão em veia subclávia associada a cateteres temporários. Estenoses venosas centrais refratárias podem ser tratadas com implante de stents, mas os resultados são ruins, com perviedade primária anual estimada em 14-25%^3,57-80^.

O conceito *fistula first* teve início com o programa *Fistula First Breakthrough Initiative* em 2003, nos EUA, com objetivo principal de modificar a prática médica referente aos acessos de diálise. Sua criação teve como base dados oriundos do Dialysis Outcomes and Practice Patterns Study (DOPPS) e das medidas de performance clínica (CPM, de *clinical performance measurements*) elaboradas pelo Centers for Medicare and Medicaid Services, órgão de repasse governamental americano. O conceito também foi desenvolvido a partir das diretrizes do consenso de acesso vascular publicado em 1997 pela National Kidney Foundation, em documento denominado Kidney Disease Outcomes Quality Initiative (KDOQI) – Clinical Practice Guidelines for Vascular Access. Na década de 90, o acesso vascular representava a principal causa de morbidade e mortalidade, sendo a falência de acesso de diálise a principal causa de hospitalizações, e suas complicações representando cerca de 14% do custo total da doença renal crônica em estágio terminal (U$ 1 bilhão/ano na época). No período, se observava uma grande quantidade de fístulas protéticas e cateteres semitunelizados (CST) e um pequeno percentual de FAVs nativas, resultando em grande número de hospitalizações, intervenções secundárias e custos elevados^81-85^.

O DOPPS é um estudo prospectivo observacional internacional com objetivo de analisar a relação de diferentes práticas de tratamento de diálise e resultados nos pacientes. Iniciado nos EUA em 1996, conta hoje com a participação de inúmeros países de quase todos os continentes, resultando em múltiplas publicações, coleta de dados de mortalidade de mais de 90.000 pacientes e acompanhamento detalhado de mais de 30.000 pacientes. Quando se analisa as publicações relacionadas ao acesso de diálise, destacam-se: (1) a FAV nativa representa a melhor opção de acesso vascular com menos complicações e necessidade de intervenção, devendo ser considerada a primeira opção de acesso; (2) uso de cateteres está associado com aumento do risco de mortalidade, aumento do risco de hospitalização e pior controle da anemia; (3) unidades de diálise com maior prevalência de FAVs nativas se dedicam a esse tipo de acesso como primeira opção; (4) a experiência do cirurgião é um fator essencial dentro do manejo do acesso vascular^86-94^.

Entre os pacientes encaminhados para confecção de FAV nativa, apenas cerca de 50-75% são elegíveis, sendo 40-50% de elegibilidade de fístulas distais e cerca de 25-35% de fístulas proximais. O problema das FAVs nativas é a falha primária, que pode ocorrer por trombose precoce (≤ 6 semanas) ou falha de maturação. O processo de maturação é complexo, e, de maneira simplificada, ocorre através de um equilíbrio entre o desenvolvimento da hiperplasia neointimal (remodelamento negativo) e vasodilatação (remodelamento positivo). Em estudo multicêntrico do Dialysis Access Consortium, Dember et al.^95^, em 2008, relataram trombose precoce em 25% das fístulas distais e 13% das fístulas proximais, além de falha de maturação de 64% versus 53% em 6 meses. Esse dado deixa claro o grande desafio de obtenção de uma FAV nativa funcional. Uma consequência natural de padronizar a FAV nativa sempre como primeira opção foi um aumento expressivo de falha de maturação, provavelmente pelo aumento de tentativas em veias e pacientes limítrofes, promovendo aumento substancial de intervenções para maturação, que acabaram resultando em custo expressivo e maior tempo de dependência de cateteres. Wasse et al.^96^ publicaram em 2007 uma maior dependência de cateteres em 90 dias após incidência da hemodiálise (60%) quando comparada a uma década antes (40%), associada a uma menor conversão para fístulas protéticas (25% versus 40%), implicando na permanência prolongada dos cateteres como acesso de transição até a maturação da fístula. Lok et al.^97^, em 2013, publicaram uma revisão de 10 anos proveniente do US Renal Data System compreendido de 2000-2010 e totalizando 1.740 acessos, demonstrando o dobro de falências primárias nas FAVs em relação aos enxertos protéticos (39,7% versus 18,8%, p < 0,001). Uma metanálise reportou um tempo médio de maturação das FAVs de 3,4 meses, com abandono desses acessos sem utilização em até 20% dos casos. Analisando a eficiência dessa modalidade de acesso, Ladak et al.^98^ em 2019 observaram que, dos pacientes submetidos exclusivamente à confecção de FAV, apenas 57% levam à independência do cateter, e apenas 40% do tempo em hemodiálise é livre de cateteres^95-104^.

Como consequência desse cenário particular e inesperado decorrente do modelo padronizado de gestão do acesso *fistula first*, muitos nefrologistas passaram a questionar o real benefício do argumento “FAV nativa deve ser sempre a primeira opção”, gerando um novo paradigma na gestão do acesso de diálise, denominado “fistula not so first, but catheter always last”. O grande diferencial dessa nova filosofia foi realizar uma análise crítica dos pontos positivos e negativos da FAV nativa e da fístula protética, reposicionando esta última no cenário principal como opção adequada de acesso e buscando particularizar situações que gerassem as condições ideais para cada tipo de acesso. Isso se deve aos resultados de estudos comparativos com análise do tipo intenção de tratar, onde se observou taxa de falência primária das FAVs nativas (32-40%) superior às fístulas protéticas (12-19%) e perviedade secundárias semelhantes, apesar de óbvias diferenças em termos de infecção e taxa de intervenção. De acordo com esse novo paradigma, o objetivo principal passa a ser reduzir dependência de cateter, independentemente se o acesso permanente é uma FAV nativa ou protética. Os critérios de escolha do melhor acesso seriam: (i) início de terapia hemodialítica; (ii) expectativa média de vida do paciente; (iii) probabilidade de falha primária de maturação; (iv) ocorrência prévia de falha de maturação. Observa-se claramente um esforço de particularização do binômio paciente-acesso, otimizando as vantagens de cada acesso em cada situação do paciente. Dessa forma, visualizando-se os dois extremos, teríamos duas situações indiscutíveis: (1) FAV nativa como primeira opção para paciente jovem, com baixa probabilidade de não maturação (masculino, não diabético, acesso preventivo e mapeamento ultrassonográfico adequado); e (2) fístula protética para paciente idoso não frágil, com baixa expectativa de vida e alta probabilidade de não maturação (sexo feminino, diabética, falha de maturação prévia de FAV nativa)^97,105-112^.

Uma última análise importante seria sobre os CSTs. Um grupo que tem sido alvo de bastante análise e, consequentemente, publicações é dos idosos. Nada mais racional do que tentar particularizar esse grupo tão heterogêneo. De um lado, pacientes idosos não frágeis, não diabéticos e com boa expectativa de vida, e no outro extremo, idoso frágeis, com múltiplas comorbidades cardiovasculares e com baixa expectativa de vida. Dentro desse contexto é fundamental uma análise mais detalhada sobre as publicações e dados inerentes aos cateteres de diálise. Em 2020, De Clerck et al.^111^ publicaram um artigo retrospectivo correlacionando tipo de acesso e mortalidade. Diferentes de seus pares, realizaram análise longitudinal da amostra ao longo de 11 anos e puderam obter dados não apenas sobre o acesso vascular incidente, mas em relação ao impacto da mudança do tipo de acesso vascular no decorrer do tempo. Quando foi analisado apenas o grupo de pacientes que mantiveram o mesmo tipo de acesso ao longo do tempo, não houve diferença estatisticamente significativa entre paciente com fístula ou cateter. Quando realizada comparação de mortalidade baseada no acesso vascular como variável ao longo do tempo, houve redução de 39% no grupo de fístula (OR = 0.61, p = 0.005, 95% CI 0.44-0.87). Todavia, em análise multivariável entre o grupo de acesso e cateter não houve diferença estatisticamente significativa (OR = 0.92, p = 0.722, 95% CI 0.58-1.46), sendo apenas significativos os parâmetros idade, antecedente de insuficiência cardíaca e câncer. Em 2017, Ravani et al.^112^ analisaram dados do DOPPS no período compreendido entre 1996 e 2011, referentes a tipo de acesso vascular, complicações dos acesso e mortalidade, e concluíram que as diferentes taxas de mortalidade não poderiam ser explicadas apenas pelas complicações inerentes aos tipos de acesso vascular. Ko et al.^113^, em 2020, demonstraram que em octogenários que iniciaram terapia dialítica com cateter e mudaram para FAV nativa dentro do primeiro ano é comparável a pacientes que iniciam com FAV e com melhores resultados em relação a pacientes que mantém o cateter com acesso vascular definitivo^19,112-115^.

Em 2020, Lok et al.^19^ publicaram a atualização da KDOQI Vascular Access Guideline, destacando a importância da particularização do acesso vascular. Poderíamos resumir o paradigma atual do manejo do acesso de diálise em “fistula not so first and catheter not so last”^19^. O aprendizado das últimas décadas ensinou que cada tipo de acesso tem suas vantagens e benefícios, sendo o grande desafio da equipe multidisciplinar que conduz o paciente com doença renal crônica elaborar um plano de gestão que possa otimizar as vantagens de cada tipo de acesso em cada realidade, destacando-se diferenças étnicas, sociais, econômicas e culturais entre os continentes. Podemos resumir a filosofia atual como o acesso ideal para um paciente ideal na circunstância ideal.

#### Pergunta 5 – É possível recuperar um cateter de longa permanência com disfunção?

Sim. Se houver acotovelamentos no trajeto, estes devem ser desfeitos com auxílio de fio guia rígido ou revisão ou reconfecção cirúrgica do túnel, com ou sem a troca do cateter (nível de evidência 1B).

Se a ponta do cateter estiver mal posicionada, reposicionar ou trocar o cateter com auxílio de fio guia rígido. A troca deve ser feita por um cateter de maior comprimento (nível de evidência 1B).

Posicionar o paciente em Trendenlenburg e ou infundir solução salina pode reestabelecer o funcionamento do cateter em alguns casos. Essa manobra pode ser tentada, principalmente quando não se identifica causa mecânica óbvia para a disfunção (nível de evidência 1C).

Quando ocorrer disfunção tardia do cateter, a trombose extrínseca deve ser excluída (nível de evidência 1B).

A trombose intrínseca deve ser tratada com a infusão de trombolítico em ambas as vias. O procedimento deve ser tentado 1 ou 2 vezes. Alteplase, 2 mg em cada via, por 30 a 60 min é a terapia de escolha (nível de evidência 1A).

Na suspeita de bainha de fibrina, a troca do cateter por fio guia rígido é necessária (nível de evidência 1A).

Não há dados que justifiquem ou contraindiquem a ruptura da bainha de fibrina com cateter balão antes do reposicionamento de um novo cateter. Assim, essa decisão deve ser individual (nível de evidência 2B).

A recidiva precoce da disfunção após sucesso inicial com a infusão de trombolítico muito frequentemente se deve à presença da banha de fibrina (nível de evidência 1B).

Trombose aguda da veia cava superior/átrio direito/veias centrais relacionada ao cateter – trombose extrínseca – deve ser tratada com anticoagulação plena. A droga inicial deve ser a heparina não fracionada em infusão contínua (nível de evidência 1A).

##### Justificativa

A disfunção do cateter de hemodiálise é encontrada quando este não permite a realização da hemodiálise nos primeiros 60 min da sessão, após pelo menos uma tentativa de melhorar o fluxo. Suspeita-se da disfunção quando o fluxo pelo cateter é menor que 300 mL/min^116^.

Outros achados suspeitos incluem a redução do Kt/V, a presença de pressão arterial menor que 250 mmHg e/ou pressão venosa maior que 250 mmHg. A condutância do acesso – Qb/Pa – é a relação entre o fluxo na bomba (Qb) e a pressão negativa da máquina (Pa). O seu valor normal é de 2 mL/min/mmHg. A necessidade de pressões negativas maiores para manter o mesmo fluxo é outro sinal de alerta^117^.

Apesar dessas definições, o KDOQI salienta que vários pacientes, principalmente os menores de 70 kg ou que fazem hemodiálise de longa duração, dialisam com fluxos menores que 300 mL/min sem que isso seja um problema. Além disso, muitos cateteres apresentam disfunções temporárias ou intermitentes, funcionando normalmente nas próximas sessões. Dessa forma, critérios mais objetivos que definam a disfunção do acesso são necessários^19^.

As disfunções são classificadas em imediatas ou tardias. As imediatas ocorrem já no primeiro uso após o implante. Usualmente são causadas por mal posicionamento da ponta do cateter ou acotovelamentos no trajeto. A ponta do cateter deve, idealmente, ser posicionada no átrio direito. Quando na veia cava superior, pode impedir o fluxo adequado. Isto é mais frequente nos pacientes obesos – pois a ponta do cateter pode se mover pelo subcutâneo quando o paciente fica em posição ortostática – e nas punções das veias centrais esquerdas. A radiografia simples permite descartar acotovelamentos e avaliar a posição da ponta, e, nos casos duvidosos, a injeção de contraste através da via venosa do cateter, sob fluoroscopia, permite determinar o local da ponta do cateter e do átrio direito. A injeção de solução salina e a aspiração de sangue são manobras que comprovam a perviedade^19^.

As disfunções tardias acontecem pela formação da bainha de fibrina, possivelmente a causa mais frequente, ou formação de trombos, sejam estes na ponta do cateter, na veia onde foi implantado ou no seu lúmen.

As tromboses foram classificadas como extrínsecas quando o trombo se situa na parede do vaso e no exterior do cateter, e intrínsecas, quando o trombo ocupa a luz ou está aderido à sua superfície^118^.

A trombose extrínseca representa, portanto, a trombose venosa profunda da veia cava superior, do átrio direito, da(s) veia(s) inominadas – nos cateteres curtos e mal posicionados – e ou da veia cava inferior. A apresentação clínica é variável, podendo gerar sintomas da síndrome de veia cava superior e mesmo embolia pulmonar, com as consequências já conhecidas. Não há dados específicos sobre o tratamento da trombose aguda da veia cava superior ou do átrio direito, sendo estas relacionadas ou não à cateter. De forma geral, são tratadas como as demais tromboses venosas profundas, estando a anticoagulação plena indicada. A heparina não fracionada em infusão contínua é a droga de escolha por se tratar de pacientes com disfunção renal grave, o que limita o uso de heparinas de baixo peso molecular, e em planejamento de intervenção cirúrgica breve. A remoção do cateter não é obrigatória, mas muitas vezes necessária, e deve ser aventada na presença de infecção relacionada ao cateter ou evolução clínica insatisfatória a despeito da anticoagulação adequada. A troca deve ser por fio guia rígido, de forma a preservar o trajeto para um novo cateter. Na presença de infecção, antibiótico deve ser administrado de forma parenteral^119-121^.

A trombose intrínseca pode ser causada pela formação da bainha de fibrina, que é a causa mais frequente, de trombo na ponta do cateter e ou trombo em seus lúmens. Cursa com redução ou ausência do fluxo por uma ou por ambas as vias do cateter.

A formação da bainha de fibrina se inicia já nas primeiras 24 h após o implante do cateter, em resposta à agressão sofrida pelo vaso. Após alguns dias, cobre toda a sua extensão, sendo aqui a principal causa de disfunção tardia. Manifesta-se em geral após vários dias da inserção, apesar de poder ocorrer já no primeiro dia.

O uso de trombolíticos tem alta taxa de sucesso na recanalização dos cateteres ocluídos, sendo maior que 80%. Além disso, aumenta o número de dias de uso do cateter até que seja necessária sua troca. No nosso meio, a alteplase é a droga mais utilizada, na dose de 1 a 2 mg por via. Usualmente, se injeta o trombolítico em ambas as vias. Este permanece no cateter por 30 a 60 min antes que seja aspirado e se teste o reestabelecimento do fluxo. Caso a primeira infusão não seja eficaz, uma segunda tentativa é feita, deixando o trombolítico por mais 30 a 90 min, ou mesmo até a próxima sessão de hemodiálise. Não há dados que mostrem superioridade entre o tipo de trombolítico, dose ou forma de administração^122^.

Os cateteres com bainha de fibrina usualmente não se recanalizam com a infusão de trombolítico ou apresentam recidiva precoce da disfunção após sucesso inicial. O diagnóstico da bainha de fibrina é feito através da angiografia, realizada pelo próprio cateter com disfunção, após a tração deste por alguns centímetros para fora da pele. Nesses pacientes, a ruptura/desorganização da bainha de fibrina com cateter balão de angioplastia é uma alternativa, e, para isso, posiciona-se um fio guia rígido através do cateter, como em uma troca por fio guia padrão. Após a retirada do cateter, realiza-se angioplastia com cateter balão de todo o seu trajeto. A seguir, um novo cateter é reposicionado de forma usual. Faltam, entretanto, dados que atestem a superioridade da ruptura da bainha em relação à troca isolada do cateter por fio guia.

Em relação à prevenção da disfunção do acesso, não há dados na literatura até o momento que justifiquem o uso de outras substâncias – trombolítico, citrato, entre outros – além da heparina, com o objetivo de reduzir a disfunção do cateter^123^.

#### Pergunta 6 – É mandatória a retirada do cateter de longa permanência na presença de infecção?

Não. Para o manejo da infecção relacionada ao cateter de longa permanência existem as seguintes opções: troca do cateter por fio guia, troca do cateter com confecção de novo túnel, retirada do cateter e preservação do cateter com antibioticoterapia sistêmica. A decisão entre essas quatro opções dependerá da estabilidade hemodinâmica do paciente, presença de sepse, bacteremia persistente, do germe isolado nas culturas, sinais de infecção no túnel e falência de acesso vascular.

##### Recomendações

1. Diante da suspeita de BRC e antes da administração de antibioticoterapia empírica, recomenda-se a coleta de duas amostras de sangue para hemoculturas periféricas e, caso se decida pela manutenção do cateter, realizar uma coleta simultânea de amostra de sangue dos lúmens do cateter venoso tunelizado (CVT) e de uma veia periférica. Para o diagnóstico, eles devem ser cultivados usando-se uma técnica quantitativa, ou a diferença de tempo de positividade entre os dois deverá ser calculada (nível de evidência 1B).
2. A remoção do CVT é recomendada quando houver uma infecção local complicada (tunelite), uma infecção sistêmica complicada (choque séptico, persistência de febre ou hemoculturas positivas 72 h após o início do tratamento antibiótico adequado, embolizações sépticas como endocardite, tromboflebite ou espondilodiscite) ou o paciente possui outro material protético intravascular (marca-passos, próteses endovasculares, válvulas etc.) (nível de evidência 2B).
3. Nos casos de suspeita de BRC, recomenda-se iniciar antibioticoterapia empírica sistêmica de amplo espectro, aguardando os resultados microbiológicos (nível de evidência 1A).
4. Recomenda-se inicialmente realizar tratamento simultâneo com antibioticoterapia sistêmica e vedação dos lumens do CVT com antibiótico em bacteremia não complicada relacionada ao cateter (nível de evidência 2B).
5. Após a remoção de um CVT infectado, sugere-se o implante de um novo uma vez estabelecido o tratamento antibiótico adequado e após a obtenção de hemoculturas de controle negativo. Se possível, deve ser colocado em local anatômico diferente do cateter prévio (nível de evidência 2A).
6. A retirada do CVT é recomendada em bacteremia relacionada ao cateter por microrganismos virulentos como *Staphylococcus aureus*, *Pseudomonas spp*., *Candida spp*. ou microrganismos multirresistentes (nível de evidência1B).

##### Justificativa

A infecção relacionada ao cateter de diálise (IRCD) é a complicação mais frequente e grave dos CVTs, associada a alta morbidade e mortalidade^72^. A incidência de BRC é de 2,5 a 5 episódios por 100 dias de uso do cateter, o que corresponde a uma incidência de 0,9 a 2 episódios de BRC por ano^60,62,66,124^. Em pacientes com CVTs, o risco de apresentar bacteremia é 10 vezes maior do que em pacientes com FAV nativa^125-127^. No trabalho de Shingarev et al.^68^, publicado em 2013, avaliando a história natural de 472 CVTs, o tempo médio de bacteremia relacionada aos CVTs foi de 163 dias, com 35% dos pacientes infectados em 3 meses, 54% em 6 meses e 79% em 12 meses.

As características clínicas comuns da infecção por CVT incluem febre ou calafrios, instabilidade hemodinâmica, disfunção do cateter, hipotermia, náuseas e vômitos e mal-estar generalizado^63,128,129^. A IRCD pode resultar em graves complicações, como osteomielite, endocardite, tromboflebite e morte em 5 a 10% dos pacientes^60,130,131^. Embolizações sépticas graves ocorrem mais frequentemente em infecções causadas por *S. aureus*, que é um dos microrganismos mais frequentemente isolados (10-40%)^132^.

Existem três tipos de infecção relacionada ao cateter^133,134^:

- Infecção local não complicada. Definida como a existência de sinais inflamatórios limitados a 2 cm ao redor do orifício de saída na pele, sem extensão superior ao cuff do cateter. Pode ou não estar associada à febre e bacteremia, e pode ser acompanhada de exsudato purulento pelo orifício cutâneo de saída.
- Infecção local complicada. Definida como o aparecimento de sinais flogísticos que se estendem além de 2 cm do orifício de saída na pele e no trajeto subcutâneo do cateter (tunelite). Pode ou não estar associada à febre e bacteremia, e é acompanhada de exsudato purulento.
- Infecção sistêmica ou bacteremia relacionada ao cateter. Definida como o isolamento do mesmo microrganismo no sangue e no CVT na ausência de outra fonte de infecção. Considera-se infecção sistêmica complicada quando há choque séptico, persistência da febre e/ou hemoculturas positivas 48-72 h após o início da antibioticoterapia adequada ou complicações embólicas (endocardite, tromboflebite ou espondilodiscite).

##### Diagnóstico de BRC

As manifestações clínicas mais sensíveis, embora pouco específicas para o diagnóstico de BRC, são febre e/ou calafrios^63,65,135^, enquanto a presença de exsudato ou sinais flogísticos locais no orifício de saída do CVT são mais específicas, mas bem menos sensíveis. Na maioria dos casos de BRC, não há evidência de infecção do orifício de entrada^136^. Outras manifestações clínicas menos frequentes são instabilidade hemodinâmica, alteração do nível de consciência, disfunção do cateter e sinais ou sintomas relacionados à sepse. Algumas vezes, as complicações da bacteremia (endocardite, artrite séptica, osteomielite ou abscessos) podem ser a primeira manifestação do BRC.

Deve-se suspeitar clinicamente de BRC quando um paciente com CVT apresentar sintomas de febre ou calafrios e/ou qualquer alteração clínica ou hemodinâmica sugestiva. Essa suspeita é reforçada se esse episódio estiver associado à manipulação ou sinais inflamatórios locais no ponto de inserção ou no túnel subcutâneo do cateter. A avaliação do episódio será então realizada por meio de história clínica e exame físico básico, a fim de excluir outras possíveis fontes de infecção além do CVT.

Critérios clínicos isolados são insuficientes para estabelecer o diagnóstico de BRC, que envolve avaliação clínica e confirmação microbiológica por hemoculturas e/ou culturas do cateter. As técnicas diagnósticas de referência baseiam-se na cultura da ponta do cateter após sua retirada^137-141^; assim, o diagnóstico de BRC é estabelecido com a positividade da referida cultura e o isolamento deste microrganismo na hemocultura. É importante um diagnóstico preciso para evitar a remoção injustificada do CVT e o risco potencial associado à colocação de um novo em outro sítio. Da mesma forma, é importante considerar que a remoção do CVT nem sempre é necessária para um diagnóstico e tratamento adequados^63,65,142-145^.

A técnica de hemoculturas quantitativas obtidas simultaneamente pelo cateter e a coleta direta de uma veia periférica (razão do número de unidades formadoras de colônias por mililitro [UFC/mL] de 3:1 a 10:1) é considerada indicativa de BRC, com uma sensibilidade de 79-94% e uma especificidade de 94-100%^146-151^.

Apesar de sua alta especificidade, essa técnica não é um método rotineiro na maioria dos laboratórios de microbiologia, devido à sua complexidade e custo. Como muitos hospitais possuem dispositivos automáticos para detecção de crescimento microbiano em amostras de sangue, foi proposto um método alternativo às hemoculturas quantitativas, que mede a diferença de tempo na positividade das hemoculturas obtidas simultaneamente pelo CVT e por punção venosa direta. A base dessa técnica está no fato de que o tempo de positividade das amostras de sangue está diretamente relacionado ao número de microrganismos inicialmente presentes na amostra^152^, de modo que, quando a positividade das hemoculturas extraídas pelo CVT ocorre pelo menos 2 h antes daquelas obtidas após punção de veia periférica, considera-se que há um diferencial de tempo positivo. O cálculo do tempo diferencial apresenta sensibilidade de 94% e especificidade de 91% para o diagnóstico de BRC em pacientes com CVT^152,153^.

Em caso de suspeita de BRC e antes da administração de antibióticos, devem ser extraídas duas hemoculturas por punção venosa obtidas de locais diferentes ou separadas entre si por 10 a 15 min. Após a retirada do CVT, proceder à cultura da ponta. Nos casos em que não há indicação de retirada imediata do CVT, será realizada uma coleta simultânea de sangue através de todos os lúmens do cateter e da veia periférica.

De acordo com a *guideline* do KDOQI 2019^19^, o diagnóstico de BRC é definido como:

- manifestações clínicas sugestivas com pelo menos 1 hemocultura positiva coletada do circuito de diálise ou periférica e nenhuma outra fonte aparente, com cultura positiva semiquantitativa (> 15 UFC/segmento de cateter, que pode ser do hub ou a ponta) ou quantitativa (> 10^2^ UFC/segmento de cateter, que pode ser do hub ou a ponta), onde o mesmo organismo (espécie e antibiograma) é isolado do segmento do cateter e de uma amostra de sangue periférica (circuito de diálise ou veia). Reforçariam o diagnóstico: culturas quantitativas simultâneas de amostras de sangue com uma razão ≥3:1hub/ponta do cateter x periférico [circuito de diálise/veia]); diferença de até 2 h na positivação da hemocultura do cateter versus hemocultura periférica.
  Em resumo, a confirmação microbiológica do BRC será estabelecida quando:
- o mesmo microrganismo é isolado na ponta do CVT e em hemocultura obtida por coleta venosa periférica;
- o mesmo microrganismo é isolado em pelo menos duas hemoculturas (uma dos lúmens do CVT e a outra por coleta de veia periférica) e os critérios diagnósticos para hemoculturas quantitativas são atendidos ou um tempo diferencial positivo é calculado.

Se o microrganismo isolado em uma única hemocultura for um estafilococo coagulase-negativo, será necessário obter novas amostras de sangue para verificar se não é uma contaminação. Nos casos em que o CVT é removido por suspeita de BRC, a ponta do cateter deve ser cultivada usando técnicas quantitativas ou semiquantitativas. A colonização será considerada quando mais de 15 UFC/mL (técnica de Maki) ou mais de 10^2^ UFC/mL (técnica de Cleri) forem quantificados durante o crescimento^139-141,154^.

##### Tratamento de infecção relacionada a cateter

Os microrganismos mais frequentemente isolados no BRC são gram-positivos. Estafilococos coagulase-negativos juntamente com *S. aureus* constituem 40-80% dos casos, portanto, o tratamento inicial deve ser eficaz contra esses microrganismos enquanto se aguarda a confirmação microbiológica^60,64,142,155^. A infecção por *S. aureus* tem sido associada à alta morbidade e mortalidade^156-158^.

A BRC não estafilocócica é predominantemente devido a enterococos, corinebactérias e bacilos gram-negativos. A infecção por microrganismos gram-negativos vem aumentando nos últimos anos, podendo representar até 30-40% em alguns centros^60,64^.

O tratamento da BRC será, por um lado, o início da antibioticoterapia sistêmica e, por outro, o manejo do CVT quanto à sua retirada ou preservação. Portanto, uma vez iniciado o tratamento antibiótico, teremos que decidir entre as seguintes opções^134^:

- Retirada imediata
  - Infecção local complicada.
  - Presença de choque séptico.
  - Persistência de febre ou bacteremia 48-72 h após o início de um antibiótico adequado à sensibilidade dos microrganismos.
  - Evidência de embolização séptica (endocardite, trombos supurativos, flebite, espondilodiscite etc.).
  - Isolamento de microrganismos altamente virulentos: *S. aureus*, *Pseudomonas spp*., *Candida spp*. ou microrganismos multirresistentes.

Uma vez removido o CVT infectado, a melhor alternativa será a colocação de um novo cateter, se possível em local anatômico diferente. Embora atualmente não tenhamos evidências suficientes, recomendamos que o implante de um novo CVT deve ser realizado uma vez que o tratamento antibiótico apropriado tenha sido estabelecido e após a obtenção de hemoculturas de controle negativo.

- Vedação dos lúmens do cateter com solução antibiótica

Na BRC não complicada, o tratamento conservador pode ser tentado, mantendo o CVT em função. Experiências anteriores, em que o cateter foi mantido e o tratamento antibiótico sistêmico foi feito por via venosa (eventualmente através do próprio cateter colonizado), mostraram taxas de cura que variaram entre 32 e 74%, juntamente com um alto risco de recorrência ao interromper os antibióticos^159-161^.

Frequentemente, na BRC ocorre a formação de biofilmes que podem ocupar tanto a superfície externa quanto a superfície intraluminal do CVT^162^. Os microrganismos causadores da infecção estão localizados no interior da biocamada na superfície interna do cateter, o que os torna resistentes à ação dos antibióticos e explicaria a dificuldade de erradicar a infecção dos CVTs tratados apenas com antibióticos endovenosos^163^.

A vedação dos lumens com antibióticos associados à terapia antibiótica sistêmica concomitante pode ser uma estratégia de tratamento alternativa para preservar o cateter. Embora não haja ensaios clínicos randomizados avaliando o papel da vedação do cateter com antibióticos no tratamento de BRC, existem alguns estudos observacionais demonstrando a erradicação da bacteremia com bloqueios de antibióticos em conjunto com antibióticos sistêmicos, em comparação com a troca ou remoção do cateter em conjunto com antibióticos sistêmicos^60,128,134^.

O tratamento deve ser realizado simultaneamente, e preferencialmente o mesmo antimicrobiano deverá ser utilizado sistêmico e local. A duração do tratamento será a mesma dos antibióticos sistêmicos (geralmente 2-3 semanas, dependendo da etiologia). O acompanhamento rigoroso dos pacientes é necessário para detectar a persistência da febre, hemoculturas positivas 48-72 h após o início do tratamento antibiótico adequado à sensibilidade microbiana, aparecimento de complicações sépticas ou recorrência de BRC. Nesses casos, está indicada a remoção do CVT.

O tratamento do BRC apenas com antibióticos sistêmicos, mantendo o cateter e sem realizar a vedação, é insuficiente para a erradicação dos microrganismos do biofilme e resolução da maioria dos casos de BRC, com elevada taxa de recorrência^163^.

- Troca do CVC infectado por fio-guia

A retirada tardia de um CVT infectado (quando não há indicação de retirada imediata ou não foi possível no momento) e a troca por um novo cateter por fio-guia é considerada uma alternativa aceitável.

A troca por fio-guia tem obtido resultados semelhantes, quando comparada com a retirada imediata em diferentes estudos não randomizados^64,164-166^. A estratégia só deve ser considerada se os sintomas desaparecerem rapidamente. Portanto, recomendamos a realização da troca do cateter pelo menos 48 a 72 h após o início da antibioticoterapia, quando o paciente permanecer clinicamente estável e não houver evidência de infecção no túnel subcutâneo.

Nos casos em que, após melhora clínica com o início da antibioticoterapia, é realizada a troca por fio-guia, e posteriormente confirmada a positividade das hemoculturas, parece prudente a realização de novas hemoculturas para confirmar a resolução da bacteremia. Caso isso não tenha ocorrido, o novo cateter também deverá ser retirado.

Nos pacientes que se enquadram como falência de acesso vascular, a estratégia de troca por fio-guia também parece ser bastante aceitável, visto que a obtenção de um novo acesso pode ser tarefa complicada.

A IRCD é uma condição frequentemente encontrada na história natural dos CVTs e sua retirada deve ser individualizada, baseado na presença de sepse, bacteremia persistente, da virulência do germe isolado nas culturas, sinais de infecção no túnel e falência de acesso vascular. A retirada imediata do CVT, sem critérios rigorosos para tal, pode resultar em perda permanente do respectivo sítio de acesso e favorecer a evolução do paciente para falência de acesso vascular.

#### Pergunta 7– Existe um diâmetro mínimo ideal de artéria e veia para a indicação do acesso vascular para hemodiálise?

Não. Apesar dos inúmeros relatos na tentativa de identificar o diâmetro mínimo aceito para criação de um acesso, não há consenso entre os estudos existentes. A evidência atual não permite fazer uma recomendação sobre o diâmetro mínimo dos vasos utilizados para a FAV (nível de evidência 2C).

##### Justificativa

As FAVs autógenas para hemodiálise são preferenciais em relação a outras modalidades, pois estão associadas a melhores taxas de perviedade primária em longo prazo e reduzidos índices de infecção em relação aos enxertos sintéticos arteriovenosas ou cateteres venosos centrais^1,167^. FAVs têm menor incidência de morbidade e mortalidade porque requerem menos intervenção em relação a outros tipos de acessos. Já em situações na qual há incapacidade de prever a adequação do fluxo venoso apenas no exame clínico, o uso de veias e artérias abaixo do recomendado pode ter contribuído para maiores taxas de falha precoce de acessos autógenos^167,168^. Cada extremidade superior tem ao menos quatro locais potenciais para criação de acessos convencionais, utilizando as veias cefálica ou basílica no antebraço ou braço em tempo único ou estagiado^168^. Quando realizadas baseadas apenas no exame físico, muitas fístulas (28%–53%) nunca amadurecem adequadamente para serem usadas em sessões de hemodiálise^169^.

A combinação do exame físico detalhado associado a um ultrassom duplex pré-operatório fornece informações valiosas para os cirurgiões escolherem a melhor combinação de fluxo arterial e venoso para obter uma FAV com sucesso^167^. Traz vantagens adicionais de ser prontamente disponível, não invasivo, de baixo custo e de alta sensibilidade para avaliação da qualidade dos componentes envolvidos na criação dos acessos. Na avaliação do sistema arterial, são observadas pressões segmentares e formas de onda ao Doppler, enquanto a avaliação dos condutos venosos requer as medidas exatas dos diâmetros e se há alguma esclerose/estenose nas veias superficiais das extremidades superiores. As artérias devem ter pelo menos 2 mm de diâmetro e um fluxo de sangue superior a 500 mL/min para fornecer diálise adequada. As veias devem ter pelo menos 2,5 mm de tamanho para uma fístula, e 4 mm para enxerto sintético^1,167-169^.

Assim, ao longo dos anos, o diâmetro dos vasos envolvidos na criação dos acessos autógenos vem sendo discutido. Entende-se como razoável que, antes de se realizar o procedimento de acesso vascular, o diâmetro e a qualidade dos vasos envolvidos devem ser considerados. Não há consenso mesmo entre as diferentes diretrizes.

O KDOQI, por exemplo, admite que, embora não haja um limite mínimo de diâmetro para criar uma FAV, artérias e veias com diâmetro < 2 mm devem ser submetidas a uma avaliação cuidadosa quanto à viabilidade e qualidade para criar uma FAV funcional^19^. Da mesma forma, sugere avaliar várias características da qualidade do vaso para criação de uma FAV (tamanho, distensibilidade, fluxo e espessura da parede)^19^. Já o consenso do grupo interdisciplinar da Sociedade Espanhola de Cirurgia Vascular recomenda que artérias com diâmetro menor que 1,5 mm e veias de diâmetro inferior a 1,6 mm sejam consideradas de viabilidade duvidosa para a criação de um acesso^39^. Por fim, o consenso do grupo europeu enfatiza a necessidade de pesquisar um local alternativo para criação da FAV sempre que a medição ultrassonográfica da artéria radial for inferior a 2 mm e/ou o diâmetro venoso cefálico for inferior a 2 mm^1^.

O diâmetro venoso previamente sugerido de 2,5 mm e o arterial de 2 mm não foram validados por estudos consistentes ao longo dos anos^19,39^. Assim, o limiar incluído na diretriz de prática clínica KDOQI para acesso vascular leva em consideração poucos estudos e relatos, limitados a estudos retrospectivos de um único centro e que avaliam o diâmetro dos vasos e traz questionamentos consistentes sobre o momento, se imediato antes da cirurgia, distensibilidade com torniquete, habilidade do operador (técnico *versus* cirurgião) e localização (radiocefálica *versus* braquiocefálica)^19^. Essa variabilidade nos parâmetros relatados limita a evidência clínica necessária para fazer recomendações sobre o tamanho mínimo do lúmen venoso e arterial^19^.

Alguns estudos trazem evidências clínicas que, embora limitadas, atendem aos critérios das diretrizes para revisão e podem fornecer informações relevantes assim resumidas:

Allon et al.^170^ em 2001 conduziram em um estudo por período de 17 meses, a avaliação ultrassonográfica pré-operatória das artérias e veias dos membros superiores de forma rotineira para o planejamento de fístulas arteriovenosas. Os tipos de acesso confeccionados e seus resultados a longo prazo foram comparados com os controles históricos institucionais, que levaram em consideração apenas o exame físico. O diâmetro mínimo da veia de > 2,5 mm e diâmetro arterial de > 2,0 mm para criação de FAV e diâmetro da veia > 4,0 mm para criação de uma fístula protética foram marcos no protocolo do estudo. No geral, em comparação com controles históricos, o estudo relatou um aumento na taxa de confecção de FAV de 34 para 64%, com maior melhora em mulheres e pacientes diabéticos. O incremento geral na usabilidade da FAV para apoiar a diálise da coorte histórica foi estatisticamente insignificante (46 a 54%; p = 0,34). No entanto, houve aumento substancial na usabilidade das FAVs do antebraço, embora não tenha sido estatisticamente significativo (34 a 54%; p = 0,06).^170^

Parmar et al.^171^ em 2007 avaliaram o impacto do duplex arterial radial de rotina para mapeamento da artéria radial antes da criação da FAV^171^. O propósito foi investigar a relação entre o diâmetro da artéria radial e a patência da FAV. Realizaram dúplex antes da criação da FAV, 1 dia, 1, 4 e 12 semanas após a confecção do acesso. Distribuíram os pacientes em 2 grupos: grupo 1, 11 pacientes com artéria radial < 1,5 mm; e grupo 2, 10 pacientes com diâmetro interno da artéria radial > 1,5 mm. Verificaram que no grupo 1, 5 pacientes (45%) apresentaram trombose imediata da FAV. Todos os pacientes do grupo 2 apresentavam FAV patente em 12 semanas. Houve uma alta taxa de falha de FAV com artéria radial < 1,5 mm. Concluíram que na presença de pequenas artérias radiais, o acesso por FAV já no braço deva ser considerado.^171^

Nica et al.^172^ em 2013 relataram em artigo que o diâmetro das veias do paciente é um fator importante a ser considerado ao decidir a elegibilidade para uma fístula. Realizaram um estudo com cirurgiões internacionais, usando cenários hipotéticos de pacientes, para avaliar possíveis barreiras percebidas e contraindicações absolutas para a criação do acesso. No total, 134 cirurgiões completaram a pesquisa. O aumento de comorbidades e falha de acesso anterior foram impedimentos para a criação de FAV, assim como o diâmetro do vaso. De modo geral, 70% dos cirurgiões relataram a necessidade de uma veia com diâmetro mínimo entre 2 mm e 3 mm para a criação de um acesso vascular. Detectaram que os cirurgiões norte-americanos e europeus eram mais propensos do que os cirurgiões canadenses a permitir a criação de uma FAV com uma veia cefálica com diâmetro de apenas 1,5 a 1,9 mm. Da mesma forma, os cirurgiões europeus eram mais propensos do que os norte-americanos e canadenses a usar uma veia basílica com um diâmetro de apenas 2 a 2,5 mm. Assim, concluíram que existe uma variabilidade significativa na avaliação cirúrgica pré-operatória dos pacientes e nos critérios de elegibilidade usados ​​para a criação da fístula, e compreender as preferências dos cirurgiões pode ajudar a estabelecer uma padronização mais clara de elegibilidade de acesso^172^.

Mais recentemente, Dageforde et al.^173^ em 2015 realizaram estudo de coorte distribuindo acessos por FAV braquiocefálica ou braquiobasílica em quartis levando em consideração o diâmetro da veia: diâmetro da veia no grupo mais baixo < 2,7 mm, e no grupo mais alto > 4,1 mm^173^. Pacientes com diâmetro mínimo da veia ≥ 3,3 mm tiveram uma taxa de maturação mais alta em comparação com aqueles com diâmetro da veia < 2,7 mm (90% *versus* 63%) e < 3,2 mm (90% *versus* 79%). Os pacientes com veias com diâmetro < 2,7 mm apresentaram uma taxa de não maturação de cerca de 40%. A análise multivariada pelo modelo de regressão de Cox evidenciou que para cada 1 mm de aumento no diâmetro da veia houve uma redução de 45% no risco de não maturação do acesso, e 36% no risco de perda de perviedade primária. As taxas de patência primária de acordo com diâmetro da veia em 6, 12 e 24 meses foram de 67%, 63% e 29% para as veias < 2,7 mm, e de 90%, 67% e 58% para as veias ≥ 3,3mm (Figura 3). Os autores concluíram que as veias de diâmetros menores estão associadas à maior probabilidade de não maturação e de perda de perviedade primária. Entretanto, não foi possível fazer qualquer recomendação em relação ao mínimo diâmetro venoso para a confecção de uma fístula, não havendo evidência clínica necessária para torná-la uma diretriz^173^.

**Figura 3:** Taxas de maturação e de perviedade primária de acordo com o diâmetro da veia^173^.

Assim, ao longo dos anos e com base em opiniões de especialistas, o diâmetro mínimo da veia de > 2,5 mm e diâmetro arterial de > 2,0 mm para criação de uma FAV e diâmetro de veia de > 4,0 mm para criação de enxerto AV foram implementados e considerados seguros parta garantir a maturação de uma FAV^1,167-169,172-174^.

#### Pergunta 8 – Existe um período ideal para maturação de FAVs?

Sim. O período ideal varia entre 4 a 12 semanas, com a maioria das FAVs nativas maduras podendo ser puncionadas entre 6 a 8 semanas (nível de evidência 2C).

##### Justificativa

A maturação de uma FAV depende da obtenção do fluxo de sangue adequado para prevenir trombose e viabilizar a hemodiálise. Há necessidade de aumento de fluxo sanguíneo tanto na artéria quanto na veia. O fluxo basal da artéria braquial é em média 31 mL/min, e deve aumentar de 10 a 20 vezes^175^, juntamente com dilatação venosa progressiva. Os estudos mostram que essas adaptações já começam a acontecer imediatamente após a confecção da anastomose: em apenas 10 min, o fluxo da artéria radial pode aumentar de 20.9 mL/min para 174 mL/min^176^. A veia dilata mais rapidamente, tornando-se clinicamente evidente^84^. O diâmetro pré-operatório da veia e da artéria e o índice de pulsatilidade também influenciam as taxas e tempo de maturação. Fístulas braquiocefálicas maturam mais do que radiocefálicas^177^.

Mas qual o significado exato de uma fístula com maturação adequada? Não existe uma definição universal^178^. Na literatura, existem pelo menos três definições distintas de maturação de FAV:

1. Maturação ecográfica: são critérios estabelecidos pelo eco-Doppler. São amplamente conhecidos como a “regra dos 6”: fluxo de 600 mL/min, a veia com 6 mm de diâmetro, a uma profundidade inferior a 6 mm da pele^84^.
2. Maturação clínica: empiricamente, é a capacidade de canulação da FAV com 2 agulhas, com fluxo de sangue adequado para realizar a hemodiálise, por um período consecutivo de 30 dias^179^.
3. Maturação anatomopatológica: é o processo de *arterialização* da veia eferente. Essa veia se torna progressivamente mais calibrosa, com maior espessura da parede, em decorrência do aumento progressivo do fluxo^180^.

A definição de maturação é extremamente importante, sobretudo quando estamos tentando responder à pergunta em questão, sobre o tempo ideal para atingir maturação. Por exemplo, uma fístula pode estar “madura” ecograficamente, mas só ser utilizada meses depois. Esse viés de definição dificulta a padronização do tempo correto de maturação.

Tradicionalmente, o sexo feminino apresenta taxas de maturação inferiores ao sexo masculino. No entanto, alguns estudos apontam apenas uma necessidade maior de tempo de maturação: a mesma fístula pode demorar 22 dias a mais para maturar em mulheres do que em homens, sem nenhuma causa específica^181^. O tempo de canulação também difere muito entre países estudados. O estudo DOPPS mostrou que 74% das FAVs eram puncionadas em 30 dias no Japão, 50% na Europa e apenas 2% nos EUA. Com 2 meses, esses números aumentavam para 98% (Japão), 79% (Europa) e 36% (EUA)^91^. Se a canulação com sucesso for o principal indício de maturação adequada, podemos cometer equívocos sobre o tempo de maturação.

As diretrizes internacionais de confecção de acesso para hemodiálise orientam^84^:

1. avaliação pré-operatória com exame físico e ecografia Doppler para avaliar calibre e perviedade dos vasos;
2. técnica cirúrgica otimizada;
3. avaliação em 1-2 semanas no pós-operatório, para afastar complicações como infecção e avaliar perviedade da FAV (frêmito palpável);
4. nova avaliação em 6 semanas para possível liberação para punção;
5. tentativas de punção de 8 a 12 semanas;
6. insucesso na canulação após 12 semanas – solicitação de ecografia Doppler para avaliar possíveis causas de falha da maturação e programar intervenção.

A maioria das adaptações vasculares que acontece após a confecção da anastomose, necessárias para promover a maturação, ocorrem em 4 semanas^182^. Tentativas precoces de canulação, com cerca de 14 dias, duplicam a chance de falha desse acesso^90,178^. Em um estudo publicado em 2004 por Ravani et al.^28^, a canulação do acesso com menos de 30 dias após a sua confecção está associada a um risco significativamente estatístico de diminuição da sobrevida da fístula (HR = 1.94, 95% CI 1.34-2.82) (Figura 4)^28^. Cerca de 60% das FAVs nativas não maturam^95^. O advento de técnicas minimamente invasivas aumenta as taxas de maturação: cerca de um terço dos pacientes podem ser submetidos a algum procedimento para facilitar a maturação^183^.

**Figura 4:** Análise de Kaplan-Meier demonstrando a relação entre a canulação precoce e a diminuição da sobrevida do acesso^28^.

A falha precoce da fístula é frequentemente secundária a lesões anatômicas que podem existir em qualquer local do circuito. Lesões arteriais do *inflow* (4-8%), com a doença aterosclerótica oclusiva, podem ser causas de falha, sobretudo em idosos e diabéticos. Problemas na anastomose (variável de 4-64%) e no *swing point* venoso (bem frequente, 25-64%) são consideradas problemas adquiridos. Lesões venosas de *outflow* (frequência alta de 33-59%) ou estenoses venosas centrais (3-9%) são geralmente decorrentes de punções prévias para acessos temporários. As fístulas que não maturam podem ter mais de uma lesão associada^184^. A avaliação das fístulas com dificuldade de maturação deve ser complementada com ecografia Doppler. Esse exame não invasivo é muito superior ao exame físico simples para selecionar os pacientes que necessitam de procedimentos adicionais para promover a canulação^185^.

Fatores relacionados ao paciente também podem dificultar a maturação, como acessos prévios, níveis pressóricos basais e, durante a diálise, etnia negra ou hispânica^186^. Estudos são conflitantes em pacientes diabéticos, evidenciando uma relação complexa entre essa doença e os índices de maturação. Níveis elevados de hemoglobina glicosilada são associados à falha da FAV^187^. Uma menor biodisponibilidade de óxido nítrico e a maior prevalência de aterosclerose grave podem funcionar como preditores de não maturação^188^. A obesidade, com IMC acima de 29,5, é um fator de risco independente de insucesso^189^. Pacientes idosos, mulheres e portadores de doença arterial coronariana associada também possuem índices superiores de não maturação^190^.

Uma extensa revisão bibliográfica da Cochrane não mostrou benefício de qualquer tipo de exercício físico nos índices de maturação das FAVs^191^. No entanto, a realização de alguns exercícios é uma medida relativamente inócua e aumenta a conscientização e participação do paciente no processo de confecção do acesso. O uso de medicação antiagregante pode diminuir as taxas de trombose, mas não aumenta as taxas de maturação. Atualmente, a orientação é manter essa medicação, caso o paciente já a venha usando por outros motivos^192^.

Em resumo, apesar da magnitude do problema de acesso vascular nos pacientes renais crônicos, há uma carência de marcadores clínicos, demográficos e biológicos eficazes para predizer com exatidão sobre o tempo de maturação de uma FAV nativa.

#### Pergunta 9 – O exame clínico de rotina é recomendado para a vigilância do acesso?

Sim. Até o presente, existem fortes recomendações para que haja o monitoramento através do exame clínico de rotina. O auxílio de outros métodos deve ser secundário^84,193^ (nível de recomendação 1B).

##### Justificativa

O princípio fundamental para a vigilância rotineira do acesso vascular para a hemodiálise é a identificação e correção de possíveis estenoses com o objetivo de otimizar a qualidade da diálise e minimizar o risco de perda do acesso. O monitoramento de rotina através do exame físico deve ser realizado preferencialmente por um profissional com experiência e conhecimento, a fim de detectar indicadores clínicos da disfunção do fluxo da fístula, sendo pela literatura uma forte recomendação, porém com moderada qualidade de evidência^194-199^. A vigilância clínica pode ser complementada com a realização de exames laboratoriais obtidos regularmente nas clínicas de diálise, análise do Kt/V, dificuldade de canulação, tempo de sangramento elevado, sinais de recirculação, mensuração de fluxo, entre outros. Embora todas essas opções possam auxiliar na identificação da disfunção do acesso, não existem evidências científicas que comprovem que esses métodos são superiores ao exame clínico.

O exame clínico realizado por um profissional com experiência em acesso para hemodiálise tem altas taxas de sensibilidade e especificidade na identificação das estenoses das FAVs nativas e com enxerto de PTFE^197-199^. Um estudo prospectivo realizado por Asif et al.^197^ em 2007 avaliou a eficácia do exame físico na detecção de estenoses em comparação com a fistulografia, considerada o exame padrão-ouro na detecção das estenoses. As taxas de sensibilidade e especificidade do exame físico foram de 92% e 86% respectivamente para as lesões de *outflow*, e de 85% e 71% para as obstruções nos segmentos de *inflow*.^197^ Uma outra análise prospectiva publicada em 2008 por Campos et al.^200^ para avaliar a eficácia do exame físico na detecção de estenoses em comparação com USG Doppler encontrou taxas de sensibilidade, especificidade, valor preditivo positivo e valor preditivo negativo respectivamente de 96%, 76%, 86% e 93%. Os resultados encontrados nesses estudos demostraram que o exame clínico, quando realizado por um profissional com experiência em acesso vascular, pode ser utilizado com segurança na identificação das disfunções dos acessos vasculares para hemodiálise, suportando o seu uso de forma rotineira.

Não há um período determinado para o exame clínico de maneira uniformizada^201^. Alguns autores recomendam que a vigilância do acesso vascular seja realizada pela mensuração do fluxo mensalmente em se tratando de fístulas com próteses de PTFE, e a cada 3 meses nas FAVs nativas^196,198^. Entretanto, existe até o momento inadequada evidência para recomendarmos a vigilância de rotina do acesso através da aferição do fluxo e monitoramento da pressão, sendo esses métodos adicionais ao exame clínico, não conferindo melhora nas taxas de perviedade do acesso quando realizados de maneira isolada^198,202^.

A utilização rotineira da USG Doppler associada ao monitoramento clínico não demonstrou benefício em relação ao exame clínico isolado na diminuição nas taxas de perda do acesso1^194,203-209^. Os resultados encontrados por Tonelli et al.^210^ em uma revisão sistemática publicada em 2008 evidenciaram que a utilização do Doppler nos programas de vigilância não diminuiu as taxas de trombose e de perda dos acessos com enxerto de PTFE. Nas FAVs nativas, pode haver um benefício na diminuição das taxas de trombose; entretanto, não houve redução no risco de perda desse tipo de acesso.^210^ Baseado nesses dados, não é possível recomendar a utilização da USG Doppler na vigilância ativa das FAVs para hemodiálise, devendo o exame de imagem ser considerado na presença de algum sinal clínico de disfunção^194,199,205^

#### Pergunta 10 – Existe um tratamento padronizado para a isquemia induzida pelo acesso vascular?

Não. O tratamento dependerá de uma minuciosa avaliação clínica e de exames de imagem para determinar a gravidade da isquemia, as condições clínicas do paciente, o tipo de FAV, a funcionalidade e qualidade da FAV, a presença e localização de possíveis obstruções arteriais, a anatomia vascular do paciente e o volume de fluxo da FAV (nível de evidência – opinião de *expert*).

##### Justificativa

Após a confecção de uma FAV, ocorrem importantes alterações hemodinâmicas locais e sistêmicas devido à conexão de dois leitos vasculares distais com resistências distintas (leito arterial distal e leito venoso) ao mesmo influxo arterial^211,212^. Como consequência, grande parte do fluxo arterial é desviado para o leito venoso^213^, com menor resistência, originando *shunt* patológico que pode levar a hipofluxo ou até mesmo inversão do fluxo nas artérias distais à anastomose^211,212,214-217^. O conjunto de sinais e sintomas isquêmicos decorrentes dessas alterações é denominado síndrome do roubo da FAV (SRFAV), sendo os principais: dor no membro em repouso ou durante as sessões de hemodiálise; alterações neurológias (paresia e parestesia); e ulcerações e gangrenas digitais^213,215,217,218^. Essa situação é dramática, pois geralmente acomete os membros superiores, de grande versatilidade funcional e fundamentais para a realização de inúmeras atividades cotidianas, podendo inclusive levar a amputações^211^.

As alterações são mais significativas em fístulas realizadas com a artéria braquial ao nível do cotovelo^213-215,217-222^, podendo manifestar SRFAV clinicamente significativa em 1 a 10% das FAVs^211-215,217-220,222,223^.

O diagnóstico dessa condição é baseado na história clínica e exame físico^213,215,222^. A realização de exames complementares auxilia mais no planejamento terapêutico do que na definição diagnóstica, tendo em vista a elevada incidência de alterações assintomáticas^215,217^, como, por exemplo, a inversão do fluxo distal à anastomose, presente em 73,3% das FAVs radiocefálicas em 90,9% das FAV com enxerto de PTFE no braço em configuração reta^216^. Os exames mais utilizados são: eco-Doppler colorido, arteriografia, oximetria digital, fotopletismografia digital, avaliação da pressão digital e aferições invasivas de pressão arterial.

A melhor estratégia para tratamento sempre é a identificação dos pacientes com elevado risco para desenvolvimento da SRFAV e escolha adequada do tipo de acesso^212,222^. Os principais fatores de risco encontrados na literatura são: idade > 60 anos, sexo feminino, presença de doença arterial coronariana ou periférica, diabetes melito, uso de clopidogrel, FAV nativa com artéria braquial, FAV com enxerto de PTFE no braço em configuração reta (utilizando artéria braquial)^212,215,220-222^.

A primeira etapa no manejo terapêutico dessa doença é a classificação da sua gravidade, conforme a Tabela 3.

**Tabela 3:** Classificação clínica da gravidade da síndrome do roubo de fístula arteriovenosa.

Estágio	Sinais e sintomas	Manejo
I	Palidez / frialdade / cianose sem dor	Clínico
IIa	Dor tolerável durante exercício ou hemodiálise	Clínico
IIb	Dor intolerável durante exercício ou hemodiálise	Cirúrgico
III	Dor em repouso ou *déficit* motor	Cirúrgico
IVa	Perda tecidual limitada	Cirúrgico
IVb	Perda tecidual extensa	Cirúrgico (amputação)

Nos estágios I e IIa, o tratamento deve ser clínico, com aquecimento do membro, estímulo de exercícios para melhora da circulação colateral, analgesia e evitar lesões no membro.

No estágio IVb, o tratamento deve ser a amputação do membro, devido à sua inviabilidade.

Nos estágios IIb, III e IVa, o tratamento pode variar entre ligadura da FAV ou revascularização do membro, lembrando que a prioridade é a manutenção da vida do paciente, seguido da manutenção do membro e, por último, do acesso vascular. A ligadura do acesso é o padrão-ouro para resolução do SRFAV, porém não preserva o acesso^212,220,221^, reiniciando o árduo trabalho de obtenção de novo acesso vascular definitivo para hemodiálise. Em pacientes com elevado risco cirúrgico, com baixa expectativa de vida ou com fístulas disfuncionais e de qualidade ruim, a melhor conduta é a ligadura da FAV e obtenção de novo acesso (FAV ou cateter). A mesma estratégia é válida em pacientes com idade avançada e viabilidade de obtenção de novo acesso não complexo, onde a ligadura e confecção de nova FAV não irá prejudicar sua sobrevida.

Após confirmação da indicação de revascularização cirúrgica do membro, o próximo passo é a realização de exames de imagem para avaliação da anatomia e hemodinâmica do membro e da FAV, possibilitando o correto planejamento cirúrgico. Os exames comumente indicados são o eco-Doppler colorido e a angiografia.

A próxima etapa no planejamento terapêutico da SRFAV é a exclusão de estenoses hemodinamicamente significativas proximais ou distais à anastomose, pois são de mais fácil tratamento, podendo ser realizada pela técnica endovascular e com bons resultados na resolução dos sintomas^211,213^. Após exclusão das lesões, o tratamento é baseado na realização de intervenções cirúrgicas, sendo as principais opções: ligadura da FAV, bandagem, cirurgia de revascularização distal com ligadura de intervalo (DRIL, do inglês d*istal revascularization with interval ligation*, como demonstrado nas Figuras 5A e 5B)^217^, cirurgia de revisão utilizando influxo distal (RUDI, do inglês *revision using distal inflow,* conforme as Figuras 5A e 5C)^219^, cirurgia de proximalização do influxo arterial (PAI, do inglês *proximalization of arterial inflow*, Figura 6)^220^ e ligadura arterial distal à anastomose.

**Figura 5:** (A) Representação esquemática de fístula arteriovenosa braquiocefálica. Adaptada de Minion et al.^219^. (B) Representação esquemática da cirurgia de revascularização distal com ligadura de intervalo, que consiste na realização de ligadura da artéria braquial distal à anastomose, com interrupção do fluxo reverso, e revascularização distal com ponte, sendo a anastomose proximal 5 cm acima da anastomose arteriovenosa, evitando assim zona de baixa pressão arterial. Adaptada de Minion et al.^219^. (C) Representação esquemática da cirurgia de revisão utilizando influxo distal, que consiste na realização de ponte da artéria radial proximal (cerca de 2 a 3 cm após a sua origem) para a veia da fístula arteriovenosa, com ligadura da mesma justa anastomose arteriovenosa. Adaptada de Minion et al.^219^.

**Figura 6:** Representação esquemática da cirurgia de proximalização do influxo arterial, que consiste na conversão do suprimento arterial da fístula arteriovenosa para artéria proximal com maior diâmetro e fluxo (axilar), promovendo assim menor queda da pressão arterial distal à anastomose, associado a maior restrição do fluxo com a utilização de politetrafluoroetileno expandido n°4 ou 5. Adaptado de Zanow et al.^220^.

Caso se opte pela preservação do acesso, a escolha da técnica dependerá se a fístula apresentar alto débito ou não, utilizando-se como limite fluxo > 800 mL/min em FAVs nativas ou > 1000 mL/min em FAVs com prótese^212,213,220^.

Nas FAVs com alto débito, as técnicas mais indicadas são aquelas associadas à restrição do fluxo, como a bandagem e a cirurgia de RUDI^213,219^. A bandagem foi o primeiro tratamento descrito com preservação do acesso e consiste na redução do calibre da veia ou enxerto, com aumento da resistência venosa e consequente diminuição do fluxo na FAV. Apresenta como inconveniente resultados pouco previsíveis e elevada taxa de perda do acesso^213,217,220,221^, extremamente dependentes do grau de restrição provocado no fluxo^222^. Essa modalidade de tratamento atualmente é reservada para as FAVs com fluxo > 2.000 mL/min^2^. A cirurgia de RUDI possui menor casuística publicada na literatura, com bons resultados na resolução da SRFAV, principalmente nas FAVs com fluxo > 1.500 mL/min^212^, porém também está associada a algum percentual de perda do acesso em consequência da utilização de artéria doadora com menor calibre (artéria radial proximal)^218,219^.

Já nas FAVs com débito normal, as técnicas mais indicadas são aquelas que utilizam o redirecionamento do fluxo, como a cirurgia de DRIL e PAI^213^. A cirurgia de DRIL apresenta excelentes resultados tanto na resolução da SRFAV quanto na manutenção do acesso; entretanto, possui como inconveniente a necessidade de ligadura de artéria pérvia e com bom fluxo, deixando a perfusão distal do membro dependente de ponte^219,220,222^. A cirurgia de PAI apresenta resultados discretamente inferiores à cirurgia de DRIL na resolução do SRFAV, porém apresenta elevada taxa de manutenção do acesso, sem o risco de comprometimento do eixo arterial axial do membro em caso de oclusão do enxerto^212^.

A ligadura arterial distal à anastomose apresenta bons resultados tanto na resolução da SRFAV quanto na manutenção do acesso, porém só está indicada em FAV radiocefálica^212^.

É fundamental a exclusão do diagnóstico diferencial da neuropatia monomélica isquêmica, caracterizada pelo surgimento no pós-operatório imediato de dor com forte intensidade associado à paralisia, porém com pulsos distais presentes. O tratamento dessa condição consiste na ligadura cirúrgica imediata do acesso arteriovenoso.

#### **Pergunta 11** – Devemos tratar as estenoses assintomáticas relacionadas às FAVs?

Não. O tratamento das estenoses relacionadas às FAVs deverá ser realizado somente na presença de alguma disfunção clínica ou quando houver documentação de diálise inadequada/queda do KtV. Não há até o momento evidências científicas que comprovem a melhora nas taxas de perviedade e a diminuição dos índices de trombose após a realização de uma angioplastia preemptiva de uma FAV assintomática. Essa recomendação é válida para as FAVs nativas, fístulas com enxerto e para o sistema venoso central (nível de recomendação 2B).

##### Justificativa

Na maioria dos programas de hemodiálise, diferentes métodos de triagem são rotineiramente usados para a identificação precoce de uma estenose hemodinamicamente significativa do acesso^7^. Esses métodos de triagem consistem em exame físico (monitoramento) e estratégias baseadas em vigilância. O monitoramento clínico inclui a avaliação do frêmito, sopro, tempo de hemostasia após remoção da agulha e avaliação do membro. Parâmetros de hemodiálise, como velocidade da bomba e pressão transmembrana, e índices de adequação da diálise (Kt/V ou razão de redução de ureia) também fazem parte do monitoramento. A vigilância inclui medições sequenciais com análise de tendência do fluxo intra-acesso, análise de recirculação, pressão venosa dinâmica ou estática, pressão arterial ou ultrassom duplex. Segundo as recomendações do KDOQI 2006, as angioplastias preemptivas das estenoses relacionadas aos acessos vasculares deveriam ser realizadas para melhorar as taxas de perviedade do acesso e para diminuir os índices de trombose^7^. Entretanto, evidências mais recentes têm demonstrado resultados conflitantes quando consideramos como desfecho a melhora da sobrevida do acesso após uma angioplastia preemptiva. Atualmente, existem poucos trabalhos com qualidade de evidência alta que avaliaram os resultados do tratamento das FAVs com estenoses assintomáticas^1^.

Uma análise observacional publicada por Chan et al.^224^ em 2011, comparando os resultados das angioplastias preemptivas *versus* acompanhamento clínico, não evidenciou resultados significativamente estatísticos quando foram analisados como desfecho a sobrevida primária do acesso, taxas de perviedade secundária e taxas de trombose. As taxas de sobrevida primária em 12 meses foram de 53,7 por 100 acessos anos no grupo submetido à intervenção preemptiva, e de 49,6 por 100 acessos anos no grupo controle (HR = 1.02, 95% CI 0.96-1.08). A subanálise de acordo com o tipo de FAV (nativa ou PTFE) também não encontrou diferença estatística entre os grupos^224^.

Em um outro estudo prospectivo publicado em 2004, foram randomizados 64 pacientes para a análise de sobrevida das FAVs com enxerto de PTFE submetidas à angioplastia preemptiva ou acompanhamento clínico. As taxas de sobrevida e tempo de abandono do acesso foram similares entre os grupos. Os autores relataram taxas de trombose do enxerto inferiores no grupo submetido à intervenção preemptiva (72% *versus* 43%) (p = 0.04)^225^.

Por outro lado, um ensaio clínico realizado por Tessitore et al.^226^, em 2004, comparando as taxas de perviedade primária e secundárias em pacientes submetidos à angioplastia preemptiva *versus* o tratamento da estenose somente em casos de disfunção da FAV nativa, encontrou taxas de perviedade primária superiores no grupo submetido à intervenção profilática (p = 0.021). Não houve diferença estatística entre os grupos em relação às taxas de perviedade secundária (p = 0.059).

Uma revisão sistemática publicada em 2016 por Ravani et al.^227^, incluindo 14 ensaios clínicos (n = 1.390) evidenciou que a correção preemptiva das estenoses relacionadas às FAVs no geral não prolongam a sobrevida do acesso. A análise evidenciou que as intervenções preemptivas nas FAVs nativas parecem melhorar as taxas de perviedade primária (RR = 0.50, 95% CI 0.29-0.86) e diminuir a probabilidade de eventos trombóticos (RR = 0.50, 95% CI 0.35 a 0.71). Entretanto, a metanálise não demonstrou aumento nas taxas de perviedade primária após as intervenções profiláticas nas fístulas arteriovenosas com enxerto de PTFE (RR = 0.87, 95% CI 0.69-1.11), bem como na diminuição de eventos trombóticos (RR = 0.95, 95% CI 0.80-1.12). Existe evidência de moderada qualidade de que situações potencialmente evitáveis de falência do acesso provavelmente não serão reduzidas significativamente pela intervenção preemptiva, independentemente do seu tipo.

Nas FAVs, a vigilância técnica e correção preemptiva parecem ter um efeito mais significativo, mas é necessária alguma precaução na interpretação dos efeitos relativos e absolutos obtidos nesta revisão. É importante ressaltar que esses resultados foram fortemente influenciados por três pequenos estudos realizados num mesmo centro^226,228,229^. Além disso, estima-se que em média a correção preemptiva de 100 estenoses pode evitar a perda de 5 fístulas e prevenir a trombose de 20 acessos – entretanto, essa virtual melhora está associada a um aumento adicional de 23,4 procedimentos, que podem aumentar o risco de eventos adversos, custos para o sistema de saúde e mortalidade. No geral, a qualidade das evidências foi baixa, a maioria dos estudos apresentou alto risco de viés, o número de estudos foi pequeno, com poucos participantes e com alta probabilidade de resultados falso-positivos.

Em relação ao território venoso central (veias subclávia, jugular interna, braquiocefálica e cava superior), as manifestações clínicas variam desde quadros assintomáticos e com pouca repercussão clínicas até apresentações com hipertensão venosa grave com aparecimento de lesões cutâneas, úlceras e diálise inadequada. Aproximadamente 15 a 20% dos pacientes em hemodiálise apresentarão algum sinal ou sintoma de estenose venosa central, e na maioria das vezes existe a associação com o uso de CVC prévio^230-233^.

As evidências disponíveis na literatura atualmente recomendam que a realização de uma angioplastia venosa central deve ser evitada em pacientes assintomáticos, devido ao risco de piora da estenose e rápida progressão para uma obstrução sintomática^1,12,19,234-236^. Uma análise retrospectiva de pacientes em hemodiálise com estenose venosa central assintomática, realizada por Levit et al.^236^ em 2006, demonstrou que os pacientes submetidos à angioplastia apresentaram maior probabilidade de progressão da estenose e piora dos sintomas. No grupo acompanhado clinicamente, nenhum paciente apresentou progressão para doença sintomática, enquanto 8% dos pacientes no grupo submetido à intervenção evoluíram com piora da sintomatologia.^236^ Em 2012, Renaud et al.^237^ avaliaram retrospectivamente 103 pacientes com estenoses venosas assintomáticas (n = 53) e com sintomas (n = 50). Os pacientes que não apresentavam sintomas foram acompanhados clinicamente, enquanto o grupo de pacientes sintomáticos foi submetido à angioplastia com balões ou stents. As taxas de perviedade primária aos 12 meses (avaliadas através do aparecimento/retorno dos sintomas) foram significativamente superiores no grupo onde não houve intervenção (77% *versus* 55%) (p = 0.002)^237^. Ehrie et al.^238^, em 2017, e Chang et al.^239^, em 2004, descrevem achados semelhantes e sugerem que a evolução clínica dos pacientes submetidos à angioplastia parece ser mais agressiva do que nos pacientes acompanhados sem intervenção. Muito provavelmente a lesão endotelial induzida pela angioplastia leva à formação de hiperplasia intimal e reestenoses mais graves que as lesões originais, justificando a piora dos sintomas.

Assim sendo, com as evidências disponíveis na literatura até o presente momento, não recomendamos a realização de intervenção preemptiva nas FAVs com enxerto de PTFE sem sinais de disfunção e nas estenoses venosas centrais assintomáticas com o objetivo de elevar o tempo de sobrevida do acesso. Nas FAVs nativas, parece haver algum benefício na angioplastia preemptiva. Entretanto, essa possível melhora na sobrevida do acesso pode estar associada ao aumento das taxas de complicações, infecções e mortalidade. Além disso, os dados são provenientes de estudos com alto risco de viés, baixa qualidade de evidências e com a maioria dos pacientes provenientes do mesmo centro, fazendo com que o grupo recomende a realização de intervenções somente nas FAVs que apresentem sinais clínicos de disfunção.

#### Pergunta 12 – Existe uma modalidade preferencial de anestesia na criação das FAVs?

Sim. O bloqueio do plexo braquial apresenta vantagens quando comparado à anestesia local. Existem ensaios clínicos randomizados e metanálise demonstrando maior perviedade a curto prazo quando os pacientes são submetidos ao bloqueio do plexo braquial comparados à anestesia local^240-244^. Esse benefício é maior para as fístulas abaixo do cotovelo^245,246^ (nível de recomendação 1A).

##### Justificativa

A maioria das fístulas e próteses arteriovenosas pode ser facilmente confeccionada utilizando-se anestesia local com lidocaína ou bupivacaína sem vasoconstritor. A ropivacaína apresenta atividade vasoconstritora intrínseca, podendo causar vasoconstrição e dificuldade no aceso de vasos com calibres reduzidos. O bloqueio do plexo braquial causa a vasodilatação e pode ajudar no manejo dos vasos no caso dos acessos para hemodiálise. Observou-se aumento dos diâmetros venosos e arteriais e do fluxo arterial em membros submetidos ao bloqueio do plexo braquial^247-249^. Entretanto, essa modalidade anestésica exige equipe anestesiológica treinada e não está globalmente disponível. Alguns trabalhos demonstraram maior taxa de fístulas distais e menor uso de prótese quando o bloqueio regional é utilizado^245,247,250^.

Em um trabalho prospectivo e randomizado com 50 pacientes, publicado por Yildirim em 2006^251^, 25 receberam bloqueio do gânglio estrelado e 25 foram controle. No grupo com bloqueio do gânglio estrelado, houve melhores taxas de maturação. Não houve diferença estatística em relação à perviedade^251^. Em metanálise sobre o tema, publicada em 2017, por Cerneviciute et al.^244^, preencheram os critérios de inclusão 4 ensaios clínicos prospectivos e randomizados. Meena et al.^241^ em 2011, em estudo com 60 pacientes, 30 em cada grupo, encontraram maior perviedade e maior fluxo venoso em pacientes submetidos a bloqueio de plexo braquial quando comparados à anestesia local. Em 2011, Sahin et al.^242^, em estudo com 60 pacientes, encontrou maior fluxo pela fístula e maiores perviedades no grupo submetido ao bloqueio de plexo braquial. Lo Monte et al.^240^, em 2011, em estudo com 40 pacientes, encontrou maior diâmetro venoso e menor resistência vascular nos pacientes submetidos ao bloqueio do plexo braquial. Aitken et al.^243^, em 2016, observou em estudo randomizado, comparando anestesia local ao bloqueio de plexo braquial, com 63 pacientes, em cada braço, maior taxa de perviedade em 3 meses (84% *versus* 62%) no grupo com bloqueio de plexo. Observou-se também maior número de pacientes com fístula radiocefálica no grupo com bloqueio de plexo (77% *versus* 48%)^243^.

Entretanto, desfechos importantes, como perviedade a longo prazo, e efeitos vasculares dos anestésicos locais no plexo braquial não foram amplamente estudados. Não existem evidências robustas para as fístulas acima do cotovelo e para as próteses arteriovenosas.

#### Pergunta 13 – Na presença de infecção de FAV está indicada a desativação do acesso?

Não. Se o paciente estiver estável hemodinamicamente, sem acometimento da anastomose pela infecção, sem sangramento ativo ameaçador da vida e respondendo ao tratamento conservador com antibioticoterapia e a medidas cirúrgicas adjuvantes, é possível tentar preservar o acesso infectado (nível de evidência – opinião de *expert*).

##### Justificativa

A taxa de infecção das FAVs nativas é tipicamente menor (entre 2 e 4%) se comparada às fistulas com prótese^102,190^. A incidência das infecções em fístulas com prótese varia de 1.6 até 35%, sendo responsável por até 35% das perdas desse tipo de acesso. O PTFE, material mais comum das fistulas com prótese, é poroso, facilitando a formação de biofilme, o que permite a proliferação de germes resistentes às defesas inatas e aos antibióticos^252-259^. Em termos de temporalidade, há um pico de infecção até 4 semanas da confecção do acesso, e, posteriormente, uma curva ascendente com o tempo^260^.

Os microrganismos mais encontrados são *estafilococos aureus* e *estreptococos epidermides* nas fístulas em membros superiores e germes gram-negativos em membros inferiores. Também é possível o surgimento de uma flora polimicrobiana e infecções fúngicas^253,261-266^.

Após eventos de infecção de fístula, medidas de prevenção e educação em saúde relacionadas à higiene pessoal do paciente e cuidados de antissepsia dos profissionais são fundamentais, uma vez que ambos, quebra na técnica de antissepsia e má higiene pessoal, são fatores de risco para infecção^263^. Outros fatores de risco conhecidos são: diabetes *mellitus*, hipoalbuminemia, idade avançada, dificuldade de canulação, formação de hematomas após punção, aumento de sangramento após retirada de agulhas, infecção por HIV, infecções em outros sítios, número aumentado de revisões cirúrgicas, obesidade e próteses trombosadas e abandonadas previamente e *bottonhole* ^267-270^.

Em 2012, Kim et al.^271^, avaliando o uso de stents revestidos para tratamento de pseudoaneurisma em próteses, encontraram uma incidência maior de infecção relacionada aos stents revestidos quando comparada a stents convencionais e revestidos utilizados neste território, por outros motivos (42% *versus* 18%). O sítio de liberação também parece interferir na taxa de infecção, havendo uma taxa maior de infecção nos stents liberados no corpo da prótese quando comparado a outros sítios, como *outflow* venoso e anastomose arteriovenosa (26.6% *versus* 6.9%)^271^.

O diagnóstico de infecção é clínico, baseado em achados do exame físico e anamnese. O paciente pode apresentar dor, hiperemia, endurecimento local, semelhante a uma celulite, pode progredir para saída de secreção purulenta com ou sem a formação de abscesso, pseudoaneurismas, ulcerações e ainda pode ocorrer síndrome hemorrágica, com erosão e sangramento de grande volume. Em muitos episódios, um sangramento sentinela ocorre anteriormente à ruptura franca. Em casos extremos, há ainda a possibilidade de evolução para sepse e morte^254-256,265,272-274^.

Há tentativas de categorização do grau de infecção e tratamento associado. Uma uniformização facilitaria a produção de ciência no tema, com possibilidade de comparação entre os dados de diversas equipes no mundo^260^.

Neste caso, a história natural do paciente seria resumida com a união de letras e números da classificação. Por exemplo, um paciente que apresentou celulite localizada, sem cultura, no qual utilizado apenas antibiótico, seria descrito como G1S0M1 (Figura 7)^260^.

**Figura 7:** Categorização de infecção de fístula arteriovenosa com enxerto. Adaptado de Kingsmore et al.^260^.

Exames de imagem, como ultrassom de partes moles com Doppler associado, podem ajudar no diagnóstico de integridade venosa ou do enxerto, descartando a degeneração para pseudoaneurismas, ou no diagnóstico de abcessos já bem delimitados. O aspecto de infiltração dos tecidos ao redor da fístula, muitas vezes visto no modo B, é uma alternativa para avaliação da extensão da infecção, excluindo, por exemplo, o acometimento da anastomose. Exames como cintilografia com leucócitos marcados ou tomografia por emissão de pósitrons podem ser utilizados no diagnóstico de infecção de prótese já abandonadas, ou em pacientes com sintomas leves e inespecíficos de infecção e sem sinais locais exuberantes^254-256,265,272-274^.

O tratamento deve ser instituído precocemente, utilizando antibióticos de largo espectro após a coleta de culturas, usualmente por 6 semanas. A conduta deverá ser individualizada de acordo com o quadro clínico, considerando a gravidade da infecção e a possibilidade de confecção de novo acesso após a resolução do processo infeccioso. O uso de antibióticos isoladamente pode ser efetivo no tratamento de infecções limitadas e localizadas (nem sempre possível nas infecções de enxertos protéticos), permitindo a manutenção do acesso. Nesses casos, a utilização da fístula deve ser evitada até a resolução completa da infecção, podendo em casos de acometimento restrito a pequenos segmentos da fístula ser permitida a utilização do acesso. Procedimentos cirúrgicos adjuvantes com drenagem e desbridamento são importantes no intuito de manutenção do acesso, principalmente em FAVs nativas. Nos acessos autólogos, quando não há uma boa resposta ao tratamento adjuvante, pode-se realizar ressecção do segmento venoso infectado, e posterior reconstrução com interposição de enxerto em trajeto tunelizado não infectado, no mesmo tempo ou em dois tempos. Nos enxertos, se o paciente está estável hemodinamicamente, com boa resposta sistêmica e local ao início dos antibióticos, e há apenas um segmento de prótese afetado pela infecção, é possível realizar explante segmentar do PTFE, com reconstrução *in situ* com crioenxerto ou reconstrução extra-anatômica com nova prótese, no mesmo tempo ou de forma seriada. Nas mesmas condições clínicas, mas com acometimento maior do corpo da prótese, com preservação da anastomose, é mandatório o explante subtotal da prótese e posterior reconstrução com interposição de enxerto em trajeto tunelizado não infectado, no mesmo tempo ou em dois tempos. Por outro lado, se o paciente apresenta síndrome hemorrágica grave, sinais de gravidade infecciosa ou acometimento da anastomose pela infecção deve-se realizar o explante completo da prótese, com reconstrução vascular arterial. Em algumas situações, em pacientes com indicação de explante completo do enxerto, mas sem acometimento da anastomose, estando esse segmento bem incorporado, é possível manter um segmento de prótese como “*patch*”, evitando reconstruções complexas arteriais, riscos de lesão neurológica e até ligadura arterial^255,256,261,275-280^. É importante ressaltar que nos casos de infecção da prótese com acometimento da anastomose e da artéria braquial, a dissecção deste segmento está associada a alto risco de lesão do nervo mediano. Nesses casos, para evitar uma perda tecidual extensa com risco de lesão neurológica, pode se optar pela ligadura da artéria braquial. A excisão do enxerto associada à ligadura desse vaso ao nível da prega cubital costuma ser efetiva e bem tolerada pelos pacientes, não resultando em isquemia crítica do membro e evitando reconstruções arteriais complexas em regiões infectadas^278^.

Com o advento de stents revestidos, em paciente com síndrome hemorrágica grave, instáveis hemodinamicamente, é possível utilizar a liberação desse tipo de dispositivo como procedimento ponte, realizando uma abordagem definitiva num segundo tempo, quando o paciente apresentar melhora clínica. É necessário ainda fazer uma individualização das abordagens segmentares e subtotais dos enxertos infectados, uma vez que as taxas de recorrência das infecções são 1,6% para os explantes totais, 19% para os subtotais e 29% para os explantes parciais^266,281,282^.

#### Pergunta 14 – Na presença de aneurisma assintomático, está indicado o tratamento cirúrgico?

Não. Os aneurismas assintomáticos relacionados com as FAVs nativas ou com prótese podem ser manejados de forma conservadora através de vigilância clínica regular, cuidados locais, orientações para que não se realize as canulações nos segmentos aneurismáticos e orientações ao paciente sobre possíveis complicações (nível de evidência – opinião de *expert*).

##### Justificativa

A dilatação do vaso após a confecção de uma FAV é uma consequência natural das mudanças hemodinâmicas e estruturais que ocorrem na circulação arterial e venosa devido ao aumento de fluxo e ao remodelamento vascular. A formação de dilatações aneurismáticas verdadeiras e pseudoaneurismas são complicações potencialmente graves que podem ocorrer na presença de uma FAV nativa ou com prótese. Os aneurismas verdadeiros são aqueles que, por definição, a dilatação envolve todas as camadas do vaso, enquanto nos pseudoaneurismas existe a perda da continuidade da parede do vaso e a estrutura de revestimento do mesmo deve-se à formação de uma parede extraluminal^283^. Os aneurismas verdadeiros geralmente estão associados ao hiperfluxo ou presença de estenoses, enquanto os pseudoaneurismas geralmente estão localizados em sítios de punção ou nas zonas anastomóticas. A definição clássica de um aneurisma é quando um vaso aumenta em pelo menos 50% o seu tamanho normal. Entretanto, não existe um valor absoluto para definir quando uma FAV é aneurismática. Devemos ressaltar que se formos considerar a definição de aneurisma à risca, uma FAV maturada deveria ser considerada uma veia aneurismática. Na tentativa de uniformizar a definição de uma FAV com dilatação aneurismática, Balaz & Bjorck^284^ em 2015 sugeriram que uma FAV deve ser considerada com aneurisma quando o diâmetro for superior a 18 mm ou aproximadamente 3 vezes o diâmetro da veia maturada. Existem outras classificações baseadas no valor absoluto do diâmetro do vaso (> 20-30 mm), no aumento do calibre em relação ao segmento adjacente (dilatação de 2-3 vezes o diâmetro proximal ou distal), na soma dos diâmetros longitudinais e transversais da dilatação ou ainda no cálculo do volume do vaso^285-289^. Por fim, outros autores recomendam um sentido amplo do termo e o definem como uma dilatação “anormal” do vaso^290^. Devido à grande variedade de definições, as taxas de incidência encontradas podem variar de 5 até 60% dos pacientes em diálise^284,291^. A história natural dos aneurismas relacionados aos acessos vasculares é pouco conhecida devido principalmente às diversas classificações existentes, à grande taxa de mortalidade e às altas taxas de perda do acesso que estão associadas à rotina do paciente dialítico. Existem diversas explicações para a formação de um aneurisma de uma FAV. A dilatação dos vasos após a confecção de uma FAV é uma resposta fisiológica e necessária para o funcionamento adequado das FAVs. Em alguns casos, essas dilatações podem se tornar patológicas, levando a formações de aneurismas sem uma justificativa plausível. O aumento da pressão no interior do circuito devido à presença de uma estenose, predisposição genética, hiperfluxo e repetidas canulações, é fator associado ao desenvolvimento dos aneurismas^287,288,290,292^. A maioria dos aneurismas é assintomático (Figura 8). Entretanto, essas dilatações podem estar acompanhadas de dor, lesões cutâneas, ulcerações, incômodos estéticos, formação de trombos, dificuldade de canulação, diálise inadequada, insuficiência cardíaca congestiva (nos casos associados ao hiperfluxo) e sangramento, que podem atentar contra a vida do paciente.

**Figura 8:** Aneurisma verdadeiro assintomático.

O diagnóstico é basicamente clínico, e o Doppler deve ser utilizado para a mensuração do diâmetro do aneurisma, análise de fluxo, presença de estenoses ou tromboses associadas. Atualmente, a qualidade das evidências existentes acerca da conduta diante dos aneurismas das FAVs é baixa, não sendo possível orientar um tratamento padronizado. Entretanto, o comportamento dessas dilatações costuma ser benigno, mantendo-se estável e sem manifestações sintomáticas durante longo período de tempo^290^. Sendo assim, o tratamento dos aneurismas não deve ser indicado nos pacientes assintomáticos, havendo a recomendação de evitar a canulação nos segmentos dilatados. Os pacientes devem ser orientados sobre a importância de se evitar a canulação nos segmentos aneurismáticos, da importância do exame físico regular para a vigilância dos aneurismas, das possíveis complicações relacionadas, do risco de sangramento em caso de ulcerações e como agir nos casos de ruptura^1,19,39,293^. O tratamento cirúrgico deve ser indicado na presença de sintomas clínicos como sangramento, ulcerações, lesões cutâneas, dor, dificuldade de canulação, inaceitável aparência estética, trombose ou hiperfluxo (Figura 8). O diâmetro isolado do aneurisma não é indicativo de abordagem cirúrgica. Existe uma preocupação maior em relação aos pseudoaneurismas em zona de canulação, pois sabemos que nesses casos há uma perda de continuidade da parede do vaso que pode estar associada com um risco mais elevado de ruptura. Como não existem evidências clínicas que comprovem um maior risco de ruptura dos pseudoaneurismas nas zonas de canulação das FAVs, segue-se a orientação de tratamento somente em pacientes sintomáticos ou nos casos de rápido crescimento dos mesmos. Lazarides et al.^294^, em 2014, recomendaram a abordagem cirúrgica das FAVs com PTFE que apresentem pseudoaneurismas com o diâmetro que exceda 2 vezes o diâmetro da prótese. Os pseudoaneurismas localizados nos segmentos de anastomose em geral são tratados com correção cirúrgica, pois na maioria das vezes estão associados à infecção. Existem diversas opções de tratamento para a correção dos aneurismas relacionados às FAVs, e, como citado anteriormente, não há trabalhos que comparem os resultados das técnicas existentes. Podemos citar como opções de tratamento: a ressecção do aneurisma com interposição de enxerto ou anastomose término-terminal, ressecção parcial, aneurismorrafia, implante de stent revestido ou ligadura do acesso^284,285,290,295-302^ (Figuras 9A e 9B). É importante ressaltar que, sempre que possível, devemos considerar a preservação do acesso, levando em consideração a possibilidade de outros sítios de confecção de FAV e expectativa de vida dos pacientes. Durante a abordagem cirúrgica, eventuais estenoses ou hiperfluxo associadas ao desenvolvimento do aneurisma devem ser corrigidas (Figuras 10A, 10B e 10C).

**Figura 9:** (A) Volumoso pseudoaneurisma em sítio de punção de enxerto de politetrafluoroetileno expandido; (B) Aspecto após correção com stent revestido.

**Figura 10:** (A) Aneurisma verdadeiro ulcerado em uma fístula braquiocefálica associado a hiperfluxo e à estenose do arco da veia cefálica. (B) Aspecto intraoperatório durante a aneurismorrafia e redução do hiperfluxo. (C) Aspecto pós-operatório.
